# α-Hydrazino Acid Insertion Governs Peptide Organization in Solution by Local Structure Ordering

**DOI:** 10.1021/acsomega.4c00804

**Published:** 2024-05-08

**Authors:** Luka Kavčič, Gregor Ilc, Baifan Wang, Kristina Vlahoviček-Kahlina, Ivanka Jerić, Janez Plavec

**Affiliations:** † Slovenian NMR Centre, National Institute of Chemistry, Ljubljana 1000, Slovenia; ‡ EN-FIST Centre of Excellence, Ljubljana 1000, Slovenia; § Division of Organic Chemistry and Biochemistry, Rudjer Bošković Institute, Zagreb 10000, Croatia; ∥ Faculty of Chemistry and Chemical Technology, University of Ljubljana, Ljubljana 1000, Slovenia

## Abstract

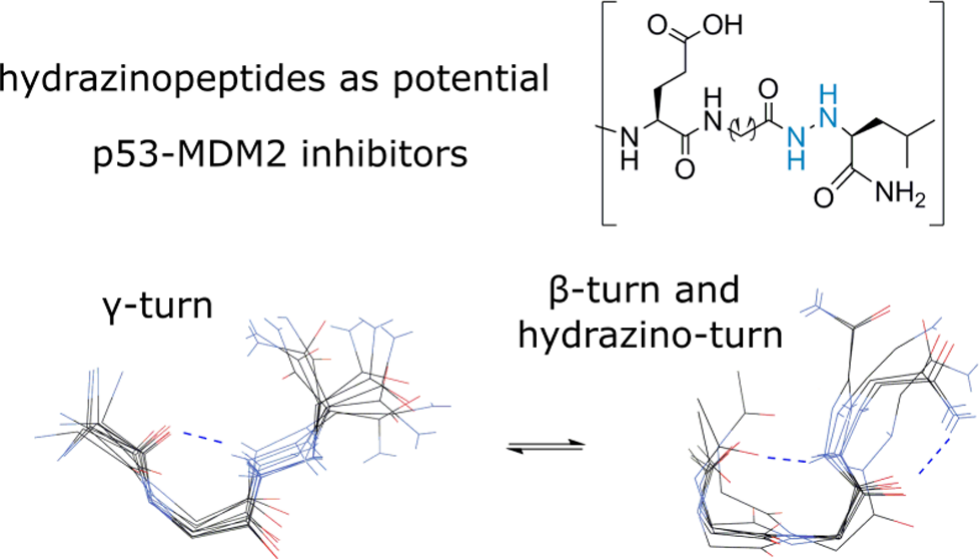

In this work, we have applied the concept of α-hydrazino acid insertion in a peptide sequence as a means of structurally organizing a potential protein–protein interactions (PPI) inhibitor. Hydrazino peptides characterized by the incorporation of an α-hydrazino acid at specific positions introduce an additional nitrogen atom into their backbone. This modification leads to a change in the electrostatic properties of the peptide and induces the restructuring of its hydrogen bonding network, resulting in conformational changes toward more stable structural motifs. Despite the successful use of synthetic hydrazino oligomers in binding to nucleic acids, the structural changes due to the incorporation of α-hydrazino acid into short natural peptides in solution are still poorly understood. Based on NMR data, we report structural models of p53-derived hydrazino peptides with elements of localized peptide structuring in the form of an α-, β-, or γ-turn as a result of hydrazino modification in the peptide backbone. The modifications could potentially lead to the preorganization of a helical secondary peptide structure in a solution that is favorable for binding to a biological receptor. Spectroscopically, we observed that the ensemble averaged rapidly interconverting conformations, including isomerization of the E–Z hydrazide bond. This further increases the adaptability by expanding the conformational space of hydrazine peptides as potential protein–protein interaction antagonists.

## Introduction

Many fundamental processes are performed by individual proteins; however, their real effectiveness relies on a complex network of interactions with other biomolecules.^1^ Central cellular events, such as cell growth, survival, and differentiation, are shaped by the network of highly selective protein–protein interactions (PPIs).^2^ Therefore, they are of utmost importance for the regulation of biological systems and are implicated in development of diseases. It has been estimated that this network or interactome contains up to 130,000 binary interactions, most of which still remain to be mapped.^3^ Modulation of PPIs has, therefore, huge therapeutic potential as PPIs are nowadays regarded as the most promising biological targets.^4−6^ Contrary to enzymes and receptors, targeting the majority of PPIs with conventional small-molecule drugs is a highly challenging task.^7^ The contact area between two proteins is usually large, flat, and exposed to solvent, lacking pockets and grooves for the binding of small molecules.

Contrary to that, peptides or peptide secondary structure mimetics can explore larger surfaces and adapt to them, therefore representing ideal candidates for PPI inhibitors.^7,8^ The binding affinity between two proteins comes from a relatively small number of key residues, known as “hot spots,” while the secondary structure acts as a pillar supporting the three-dimensional (3D) arrangement of key side-chains. Therefore, structure-based design of PPI inhibitors aims to identify hot spots by means of alanine scanning, *in silico* screening or fragment-based approach.^9^ Once validated, hot spots need to be embedded into the molecule framework designed to ensure the spatial arrangement of key residues comparable with that of the native sequence.

The importance of conformational control and proper positioning of key residues by peptide backbone modification can easily be recognized in biological systems, e.g., natural enzymatically catalyzed backbone modifications, such as the switch from l- to d-α-amino acids^9,10^ and amide nitrogen alkylation,^11,12^ or more substantial alterations, such as the formation of azole heterocycle in jellyfish green fluorescent protein.^13^ Their wide distribution in various organisms suggests their unique and important role in fine-tuning the functional properties of proteins by reducing the favorable φ and ψ torsion angle space of the peptide,^14^ affecting the *cis–trans* equilibrium of amide bonds,^15^ or providing access to unusual torsion angles normally allowed only for Gly.^16,17^ Locally, this can reshape and stabilize the peptide structure by inducing turns or other secondary structure elements, beneficial for PPI.^18^ However, to thoroughly understand and appreciate this poorly examined class of modifications, either undiscovered or already reported in nature, there is a growing interest in studying the physicochemical as well as structural properties of their synthetic analogues.

**Figure 1:**
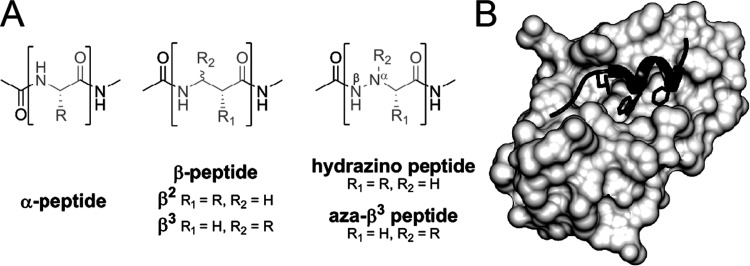
(A) Comparison between natural α-peptides, β-peptides, and their aza-analogs. The onomeric building block of different types of peptides is highlighted in brackets with *R*, *R*_1_, and *R*_2_ designating an alkyl or aryl group. In α-hydrazino peptides **h(1)**, **h(4)**, **h(8)**, and **h(1,4,8)**, *R*_2_ is H. The nitrogen atom closest to the C^α^ in α-hydrazino and aza-β^3^ peptides is labeled as N^α^ and is with N^β^ involved in a hydrazide bond. (B) Crystal structure of MDM2 oncoprotein (gray) bound to the helical segment of p53 (black). Key amino acids, Leu, Phe, and Trp, which are part of the octapeptide binding epitope, are explicitly shown with their side chains (PDB id: 1YCR).^37^

Chemical cyclization of linear peptides is the simplest strategy for introducing conformational constrains and, at the same time, increasing plasma stability.^19,20^ For example, pharmacological properties of α-helical peptides can be significantly improved by stapling, either by lactam, hydrocarbon, thioether, aryl, or by triazole-based bridges.^8,21^ A different approach based on the replacement of natural with modified amino acids or amide bonds with isosters is applied for mimicking peptide turn structures,^7^ essential binding motifs for cellular receptors, such as G-protein coupled receptors^22^ or integrins.^23^ There is a large number of backbone-modified amino acids that can noncovalently induce new structural preferences in peptidomimetics.^24^ Most prominent are the β-amino acids comprising an additional methylene group in the main chain (Figure 1A), allowing β-peptides to form distinct β-sheet-like, turn-like, and helical structures.^25,26^ Consequently, β-peptides and oligomers composed of α- and β-amino acids are often used as secondary structure mimics for targeting PPIs.^7^

Non-natural amino acids with a high propensity to initiate turn-like conformations are α-hydrazino acids and aza derivatives of β^2^-amino acids (Figure 1A).^24^ The presence of the additional nitrogen atom (N^α^) with its hydrogen bonding potential leads to the reorganization of intramolecular hydrogen bond network in oligomers, resulting in the formation of the hydrazino turn,^27,28^ a bifurcated 8-membered hydrogen-bonded ring. The incorporation of only one α-hydrazino derivative in *trans*-2-aminocyclobutanecarboxylic acid (*t*ACBC) oligomers caused structure reorganization and stabilization of thermodynamically favored helical conformation.^29^ Also, heterochiral cyclic oligomers composed of 1:1 mixtures of α-amino acids and α-hydrazino acids self-assemble into nanotubular structures in solution and solid phase.^30^ Peptides containing this type of sequence modification, known as α-hydrazino peptides, can adopt a variety of secondary structures and conformers depending on the peptide length and N^α^ and C^α^ substituents.^31^ As opposed to the more studied aza-β^3^ peptides, there is limited data available on conformational properties and pharmacological applications of α-hydrazino peptides^31−34^ due to their structural flexibility and difficulty in obtaining enantiomerically pure samples.^35^

We have recently designed a small series of α-hydrazino peptides and showed that their interaction with DNA and RNA can be finely modulated with the number and relative position of α-hydrazino acid residues within the peptide chain.^36^ All of these prompted us to consider α-hydrazino peptides as promising candidates for modulating PPIs. In this work, we applied the concept of α-hydrazino acid insertion in a peptide sequence as a means to induce structural organization of a potential PPI inhibitor. On the basis of NMR data, we report structural models of the p53-derived α-hydrazino peptides with the presence of local peptide structuring in the form of α-, β-, or γ-turn as a result of the α-hydrazino modification in the peptide backbone. The combination of all three modifications could potentially lead to the preorganization of a helical secondary peptide structure in solution, entropically favorable for binding to biological receptor (Figure 1B).

One of the widely studied and described PPI systems is the interaction between the tumor suppressor protein p53 and its natural antagonist MDM2. p53 is a transcription factor that regulates the cellular response to stress, while MDM2 downregulates its activity through a negative feedback loop by binding to the α-helical transactivation domain near the N-terminus of p53.^38−40^ Suppressed p53 cannot regulate growth arrest and cell death in the presence of DNA damage and thus directly contributes to tumor development, malignant disease progression, resistance to treatment, and poor prognosis. Therefore, inhibiting this interaction is an attractive and feasible approach for cancer therapy. Structurally, the binding interface between the α-helical segment of p53 and the MDM2 protein surface is an example of a primary peptide epitope with three key amino acids (Leu, Phe, and Trp) located within a p53 octapeptide segment (Figure 1B).^37,41^ The described PPI system serves as a model system for new concepts in this field and enables the exploration of different approaches. Therefore, a wide range of compounds, including small molecules, natural products, α- and β-peptides, β-hairpin peptoids, p-oligobenzamides, and mini-proteins, have been explored as inhibitors of this interaction.^5,42,43^

## Results and Discussion

We chose the minimal p53-derived sequence (^19^FMDYWEGL^26^) retaining micromolar affinity for MDM2 to assess the potential of α-hydrazino acids to induce conformational changes and trigger preorganization when incorporated into the peptide sequence.^44^ Even single-residue substitution of natural amino acid with its α-hydrazino counterpart causes peptide structural perturbation, which, due to its unique conformational preferences, leads to alteration in the peptide ability to interact with other biomolecules.^29,36^ This encouraged us to prepare α-hydrazino peptides that harbor modifications at three different sites in the sequence. Our motivation was to replace the “hot-spot” amino acids Leu, Phe, and Trp with their α-hydrazino derivatives. While the synthesis of α-hydrazino leucine and α-hydrazino phenylalanine proceeded smoothly, the synthesis of the α-hydrazino derivative of tryptophan proved to be difficult. Therefore, we attempted to prepare α-hydrazino derivatives of two neighboring amino acids, tyrosine and glutamic acid, but in all three cases, we experienced difficulties with the isolation and purification of the α-hydrazino acids. We succeeded in purifying only hTyr, and we decided to use it as the amino acid preceding hot-spot amino acid Trp. Thus, we substituted either the N-terminal phenylalanine (**h(1)**), the C-terminal leucine (**h(8)**) or the middle tyrosine (**h(4)**) with their α-hydrazino analogues (Table 1) at the substitution sites, which were carefully selected based on the known p53-MDM2 structural interface.^37^ To enlarge the library of α-hydrazino peptides and, more importantly, the variability of their conformational space, α-hydrazino peptide **h(1,4,8)** with all three α-hydrazino amino acid substitutions was also prepared and analyzed.

**Table 1:** Studied α-Hydrazino Peptides with their Amino Acid Sequences

	sequence
parent	F1-M2-D3–Y4-W5-E6-G7-L8
**h(1)**	**hF1**-M2-D3–Y4-W5-E6-G7-L8
**h(4)**	F1-M2-D3-**hY4**-W5-E6-G7-L8
**h(8)**	F1-M2-D3–Y4-W5-E6-G7-**hL8**
**h(1,4,8)**	**hF1**-M2-D3-**hY4**-W5-E6-G7-**hL8**

### Synthesis of α-Hydrazino Peptides

**Scheme 1:**
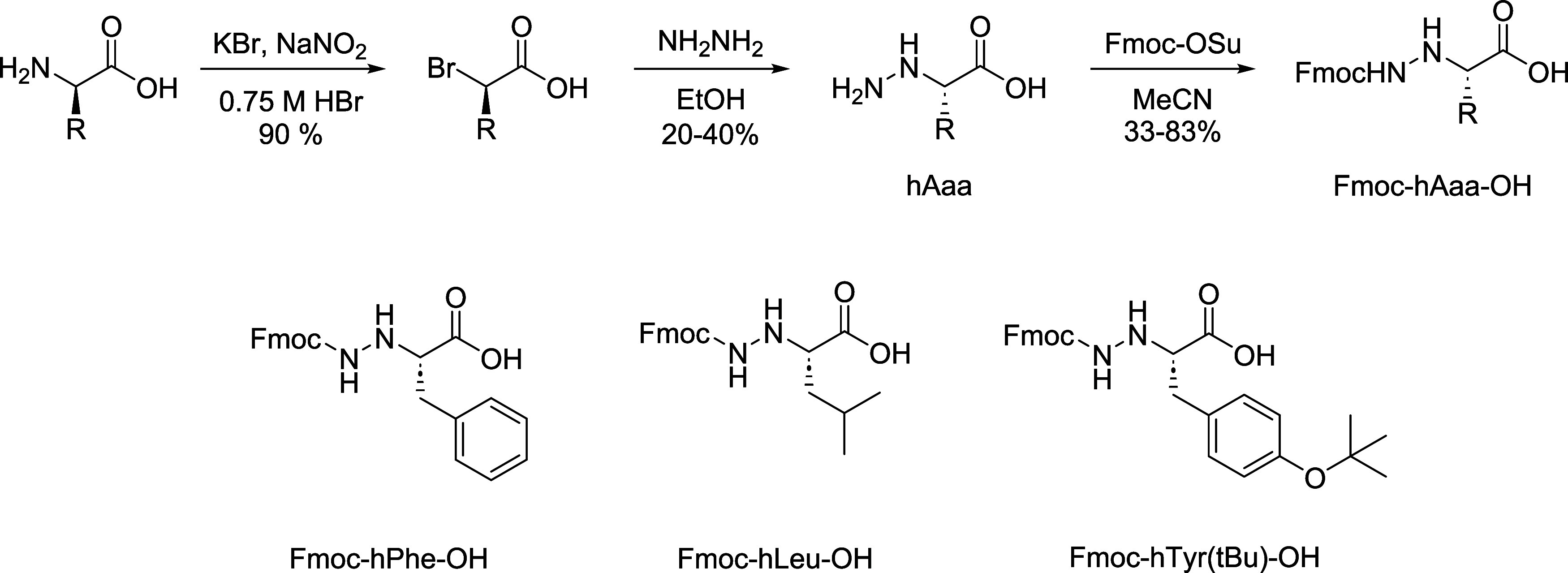
Three-Step Synthesis of α-Hydrazino Acid Analogues

Hydrazino derivatives of phenylalanine, leucine, and tyrosine were prepared by nucleophilic substitution of d-amino acid-derived α-bromo acid with hydrazine hydrate (Scheme 1, with the details in the Supporting Information).^35,45^ In the final step, the N^β^ atom was masked with an Fmoc protecting group. Fmoc derivatives of α-hydrazino acids were recrystallized and used in a solid-phase peptide synthesis (SPPS). Rink amide resin was used as the solid support and HBTU/HOBt as the coupling reagent for α-amino acids and HATU/HOBt for α-hydrazino acids (double coupling). After cleavage and deprotection, the peptides were purified by preparative high-pressure liquid chromatography (HPLC) and characterized by nuclear magnetic resonance (NMR) spectroscopy, high-resolution mass spectrometry (HRMS), and circular dichroism (CD) (Figures S1–S5 for the parent peptide, Figures S6–S10 for α-hydrazino peptide **h(1)**, Figures S11–S15 for α-hydrazino peptide **h(4)**, Figures S16–S20 for α-hydrazino peptide **h(8)**, Figures S21–S25 for α-hydrazino peptide **h(1,4,8)**, and Figure S26 for comparative analysis of CD spectra).

### Parent Peptide in the Absence of Biological Receptor Mainly Adopts an Extended Conformation in Solution

**Figure 2:**
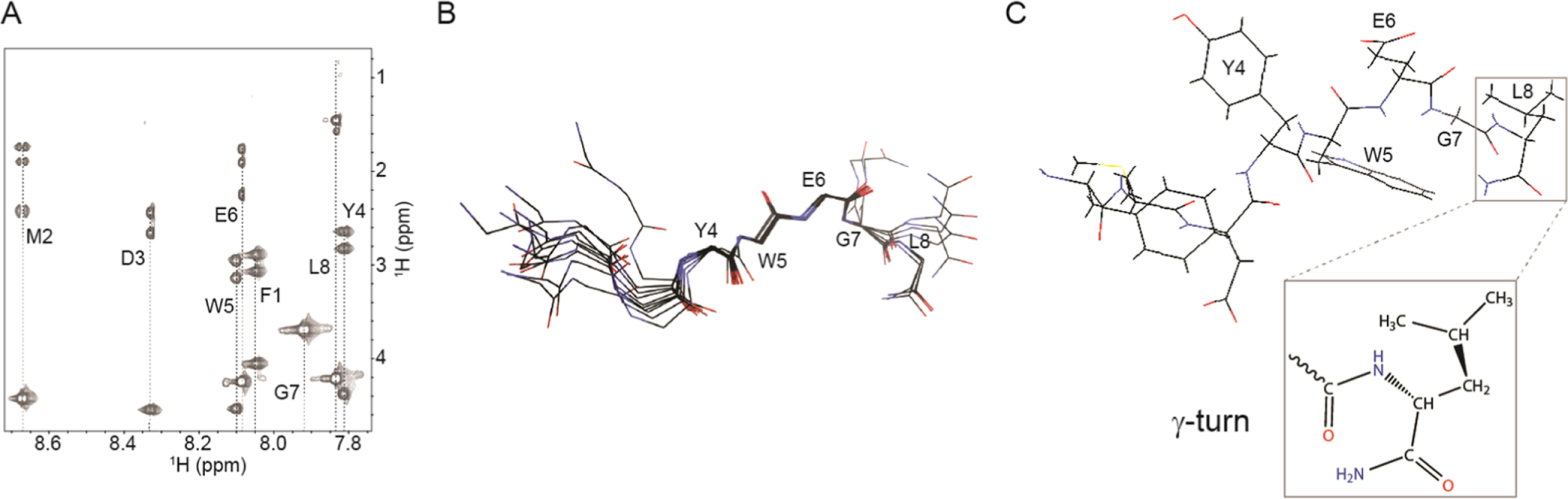
Spectroscopic properties of the parent peptide in DMSO-*d*_6_. (A) Amide-aliphatic region of the TOCSY spectrum (τ_m_ = 80 ms, 2 mM peptide conc., 25 °C, 600 MHz) showing intraresidual correlations. (B) The ensemble of the 10 lowest energy structures was calculated using NOE distance restraints. The amino acid side chains in the ensemble of structures are omitted for the sake of clarity. (C) Stick model representation for one of the calculated parent peptide conformations (in B), highlighting the formation of the γ-turn at L8 in an enlarged model.

First, we analyzed the parent octapeptide in DMSO-*d*_6_ using NMR spectroscopy. Clearly resolved signals of amide (H_N_) protons and respective cross-correlations in two-dimensional (2D) spectra enabled us to complete the peptide backbone and side-chain resonance assignments (Figure 2A). Based on the collected NOE distance restraints from the 2D ^1^H–^1^H NOESY spectrum, we calculated a family of structure models of the parent peptide in solution (statistics of structural calculation are summarized in Table S11). The absence of long-range NOE contacts indicates a lack of a well-defined secondary structure (Figure 2B). Indeed, the final NMR structural ensemble revealed that the parent peptide adopts an extended conformation with local structuring in the form of a γ-turn (Figure 2C), which could be attributed to the influence of the C-terminal amide (−C(O)NH_2_) group on the local organization.^45^ Similar features were reported by Garcia-Echeverria et al. for a dodecapeptide, containing the full sequence of the parent peptide, with the formation of an extended or random coil backbone conformation in aqueous solution.^44^

### α-Hydrazino Peptides Adopt Multiple Conformations due to E/Z Hydrazide Bond Isomerization

**Figure 3:**
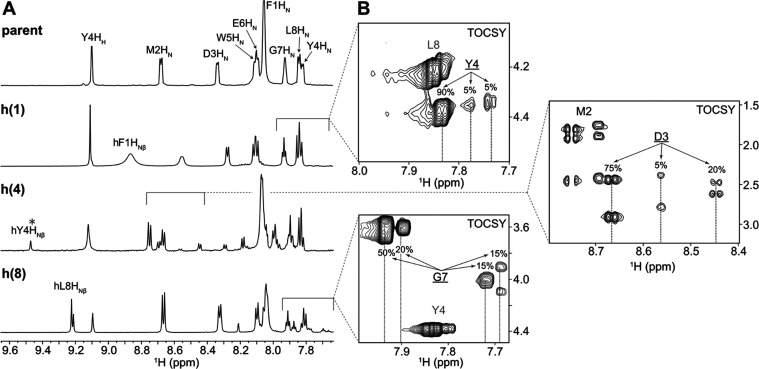
NMR spectra of single-site modified α-hydrazino peptides in DMSO-*d*_6_. (A) Amide regions of the ^1^H NMR spectra of α-hydrazino peptides **h(1)**, **h(4)**, and **h(8)** and the parent peptide with labeled H_Nβ_ signals. In the 1D NMR spectrum of the α-hydrazino peptide **h(4)**, Y4H_Nβ_* represents the signal for the minor form; we could not observe the corresponding peak for the major form. The regions in the ^1^H NMR spectra with multiple H_N_ signals for the same type of α-amino acid residues are highlighted and expanded. (B) Amide-aliphatic region of the TOCSY spectra (τ_m_ = 80 ms) showing correlations of H_N_ and H_α_ protons at 25 °C. Multiple H_N_ signals corresponding to identical protons in the α-hydrazino peptide structure are presented with arrows. The intensity ratios for different sets of intraresidual cross-peaks for a given residue (underlined) provide population estimates of the different structural forms in solution with an accuracy of ±5%. The NMR spectra were acquired at 2 mM peptide concentration and 25 °C by using a 600 MHz spectrometer.

^1^H NMR spectra of the α-hydrazino peptides **h(1)**, **h(4)**, and **h(8)** with one α-amino acid substitution showed good signal dispersion (Figure 3A). However, multiple H_N_ and H_α_ signals were observed for a particular residue, which suggested their involvement in equilibria between different peptide conformations. They were discriminated with the use of their distinct correlations in the ^1^H–^1^H TOCSY spectra (Figure 3B). We excluded the possibility of intermolecular association and oligomer formation since there were no differences in proton signal line widths.

Resonances for the atypical H_Nβ_ (termed hydrazidic) and H_Nα_ protons were observed downfield from the standard amide and H_α_ regions (Figures 3 and S27). The signal of the H_Nα_ proton is very broad and has no correlation with other peptide proton resonances in 2D NMR spectra. This points to the high tendency of the sp^3^-hybridized N^α^ atom in the α-hydrazino peptide backbone to exchange its loosely bound proton with residual water. In addition, a comparison of the chemical shifts of the main chain atoms of α-hydrazino peptides **h(1)**, **h(4)**, and **h(8)** with the parent peptide showed the largest differences for the residues adjacent to the α-hydrazino moiety (Figure S28), indicating effects of the hydrazino group with its distinct stereoelectronic properties on the chemical environment of the nuclei in close proximity.

**Figure 4:**
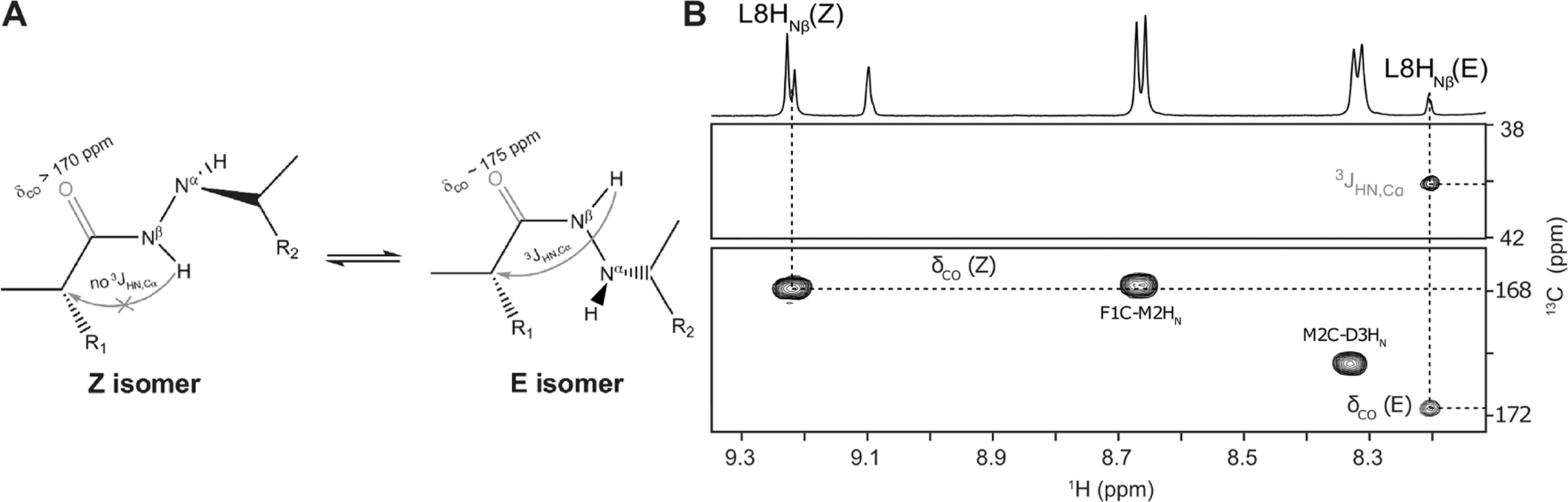
(A) E/Z conformational isomerism as an internal source of α-hydrazino peptides’ conformational equilibrium with ^13^C chemical shift of the hydrazidic carbonyl group and the presence of H_N_–C_α_ coupling (designated with arrow) as criteria for assigning both isomers from NMR spectra.^46^ (B) C_α_ (top) and carbonyl (bottom) regions of ^1^H–^13^C HMBC spectrum of α-hydrazino peptide **h(8)** showing informative correlations of L8 H_Nβ_ protons corresponding to E- and Z-α-hydrazino peptide isomer. The NMR spectra were acquired at 2 mM α-hydrazino peptide concentration and 25 °C using a 600 MHz spectrometer.

Multiple H_N_ proton resonances and the corresponding amino acid fingerprints in the TOCSY spectra indicated the presence of multiple forms for α-hydrazino peptides **h(1)**, **h(4)**, and **h(8)** in the DMSO-*d*_6_ solution. For instance, a comparison of the one-dimensional (1D) NMR spectra identifies α-hydrazino **h(4)** as the peptide with the greatest heterogeneity as it has the most signals in the amide spectral region (Figure 3A) as well as three sets of cross-peaks for D3 H_N_ in the TOCSY spectrum (Figure 4B) with D3 being the neighboring residue that was most altered by the proximity of the hydrazino group (Figure S27). Based on the presence of apparent intraresidual H_N_–H_α_ cross-peaks in 2D ^1^H–^1^H ROESY spectra indicating chemical exchange processes between different forms (Figures S29–31), we proposed that different peptide conformations are involved in a slow conformational exchange on the NMR chemical shift time scale.

Previous conformational studies of (α-amino/α-hydrazino)mers^31^ and aza-β^3^-peptides^46^ reported E/Z hydrazide bond isomerism as an intrinsic source of their conformational heterogeneity as well as proposed the criteria to discriminate between the isomers (presented in Figure 4A).^46^ The H_N_ proton resonances corresponding to the two most populated peptide forms (Figure 3) were investigated using ^1^H–^13^C HMBC spectra, where carbonyl resonances together with the absence of H_N_–C_α_ cross peak unambiguously determined the conformer (as shown in Figure 4B for α-hydrazino peptide **h(8)**). Our experimentally determined values for the ^13^C chemical shift of carbonyl groups involved in hydrazidic bonds in the studied peptides differ somewhat from the limits set by Le Grel et al.^46^ However, this discrepancy could be due to the use of DMSO-*d*_6_ as opposed to the less polar CDCl_3_. We observed differences in the carbonyl δ_CO_ values for E- and Z-isomers of around 4 ppm (for Δδ values, see Table 2), which enable us to discriminate carbonyl groups in distinct chemical environments of peptides involved in E/Z equilibrium and altogether confirm this type of isomerism as one source of structural heterogeneity of α-hydrazino peptides **h(4)** and **h(8)**.

**Table 2:** ^13^C NMR Chemical Shifts^a^ of the Hydrazidic Carbonyl Group for α-Hydrazino Peptides **h(4)** and **h(8)**

	Z-isomer	E-isomer	Δδ
α-hydrazino peptide **h(4)**	169.5	174.1	4.6
α-hydrazino peptide **h(8)**	167.9	171.8	3.9

a Reported in ppm. The NMR spectra were acquired at 2 mM α-hydrazino peptide concentration in DMSO-*d*_6_ and 25 °C using a 600 MHz spectrometer.

After assigning the isomer type based on the correlations in the ^1^H–^13^C HMBC, the subsequent NMR spectroscopic analysis was performed in the TOCSY spectrum. Based on the finely resolved proton cross-peaks, we were able to relate the estimated percentage of the population to either the E- or Z-isomer type for each set of signals in Figure 3.

For α-hydrazino peptide **h(4)**, we were able to determine the isomer nature for the two most populated peptide forms with the third conformation unassigned due to the lack of distinctive cross-peaks in NMR spectra. We have established major E- (75%) and minor Z-isomer (20%) conformations of the α-hydrazino peptide **h(4)** in solution, which could be explained by several factors regarding the hydrazide link. First, alongside similar steric hindrance between peptide side chains in the E conformation with respect to standard peptide bond (Figure S33a), repulsion between the carbonyl oxygen lone pairs and the N^α^ lone pair in the hydrazidic linkage destabilizes the Z-isomer conformation (Figure S32).^47^ Second, quantum chemistry calculations together with vibrational spectroscopy revealed that the hydrazide linkage is essentially most stable in E anticlinal conformation due to the stabilizing effect of hyperconjugation between N^α^ lone pair and hydrazidic σ_NβH_* orbital (Figure S33).^48^ Therefore, the observed prevalence of the E-hydrazide bond conformation at position Tyr4 in α-hydrazino peptide **h(4)** can be adequately explained by considering the described stereoelectronic effects of N^α^ lone pair repulsion and hyperconjugation in this system.

Equivalent analysis was performed for α-hydrazino peptide **h(8)** and revealed that sets of signals with the highest population percentages (50 and 20% on Figure 4) both correspond to the Z-isomer nature. For instance, two resonances for G7 H_N_ proton are identified for α-hydrazino peptide **h(8)** Z-isomer and exhibit NOE correlations to the same E6 residue chemical shift values in the NOESY spectrum. Interestingly, two sets of signals were also observed for the E-isomer (both 15%). Since the predominance of α-hydrazino peptide E-isomer is not in line with the previous explanation, there have to be other factors influencing the overall equilibrium. Previous studies indicated that E/Z conformational equilibrium in α-hydrazino peptides could be additionally tuned by the formation of stable local structures, such as hydrazino-turn or N^α^ pseudospiranic conformation (Figure S34), which differ by a 180° rotation around the N–N^α^ bond, leading to the stabilization of the Z-isomer.^31,46^ The high population of the Z-isomer in α-hydrazino peptide **h(8)** must, therefore, be influenced by some form of structuring (*vide infra*) as well as by the terminal position of the hydrazino group, which makes α-hydrazino peptide **h(8)** less conformationally restrained than that in α-hydrazino peptide **h(4)**, which has a bulky Tyr-side-chain flanking the hydrazide linkage.

### Single-Site α-Hydrazino Acid Modification Leads to Local Organization in the Form of Peptide Turn Structures

**Figure 5:**
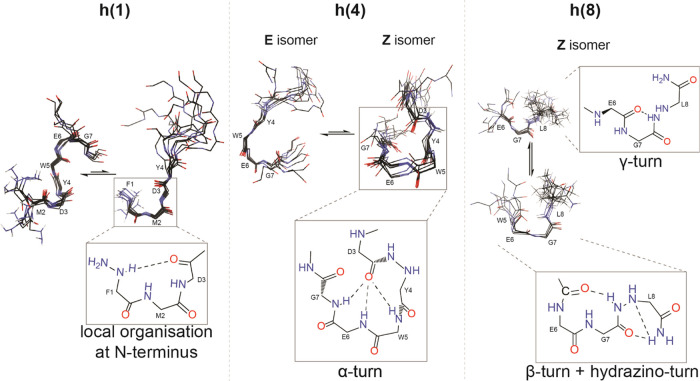
Examples of the structural organization induced by an α-hydrazino acid substitution at specific sites that were observed in proposed structural models for α-hydrazino peptides **h(1)** (left), **h(4)** (middle), and **h(8)** (right). All models were calculated using AMBER.^49^ The structural clustering for α-hydrazino peptide **h(8)** was only observed at the C-terminus of the peptide; therefore, only residues E6, G7, and hL8 are shown. The specific type of structural organization is enlarged in squares and colored by heteroatoms with the amino acid side chains omitted for clarity.

In-depth inspection of NMR spectra of α-hydrazino peptides revealed that there are other sources of conformational heterogeneity besides E/Z isomerism. Specifically, we observed two discrete sets of signals for each geometrical isomer type of α-hydrazino peptide **h(8)** (Figure S35) as well as several sets for α-hydrazino peptide **h(1)**, unaffected by E/Z isomerism (free hydrazino group at the N-terminus). By correlating separate sets of signals for each individual conformation into NOE sequential walks and extracting spatial restraints from their NOESY cross-peaks integrals, we were able to propose structural models for the two most prominent conformations of α-hydrazino peptides **h(1)**, **h(4)**, and **h(8)** in solution (Figure 5).

The calculated NMR ensembles of α-hydrazino peptide **h(1)** with the modification at the N-terminus revealed two distinct structural clusters in equilibrium with similar local structural organization (Figure 5 left side). They differ mainly in the backbone structure of the C-terminus with the formation of two successive β-turns in the first, in contrast to more terminal disorder in the second conformation. They are supported by different NOE contacts in the NOESY spectrum with the latter conformation visibly resulting from the absence of medium-range spatial restraints (Table S11). The presence of conformational equilibrium in the solution is further supported by specific antiphase correlations in the ROESY spectrum (Figure S29). Moreover, a comparison of the chemical shifts of the two observed α-hydrazino peptide **h(1)** forms and the parent peptide revealed that the minor conformation is more similar to the values obtained for the parent peptide than the major form with respect to the chemical environment of the backbone protons (Figure S36), further supporting the structural differences observed in our models.

The structural calculation for α-hydrazino peptide **h(4)** was performed separately for the major (E) and the minor (Z) forms with the conformation of the hydrazide bond predetermined. The results for the E conformation showed the local structure ordering in the peptide backbone, which forms an arc (Figure 5 middle), while the minor Z-isomer showed an α-turn conformation (Figure 5 middle). The differences in the proposed structural models are also supported by comparing the chemical shift of the backbone of α-hydrazino peptide **h(4)** with that of the parent conformation (Figure S36). The minor form shows the greatest chemical shift changes for the part of the molecule, carboxy from the α-hydrazino modification, due to the α-turn ordering, whereas the major form shows more similarities to the parent peptide in its backbone chemical environment at the same positions, translating to more parent-like conformation. We hypothesize that such structural preorganization in the form of α-turn in the Z-isomer of α-hydrazino peptide **h(4)** could enhance the binding to the MDM2 receptor by decreasing the unfavorable conformational entropy change upon binding. Additional ordering of the rest of the molecule could yield the α-helical structure, detected in the crystal structure of the p53 TAD sequence in a complex with the same receptor (Figure 1B).^37^ It may be proposed that the Z-isomer of α-hydrazino peptide **h(4)** could provide a midpoint in the folding of the α-helix, which is required for efficient MDM2 receptor binding. All in all, we propose that the Z-isomer of α-hydrazino peptide **h(4)** has a tendency to fold into an α-helix higher than that of the parent peptide in solution, as the latter forms a mainly extended conformation.

Due to the lack of distinct NOE contacts for the minor E-form of α-hydrazino peptide **h(8)**, a model calculation was possible only for its major conformer (Z). It revealed two clusters of distinct conformations (Figure 5 right), differing in the local order induced by the hydrazidic H_Nβ_ proton in the form of a parent peptide-like γ-turn (Figure 2C) or a novel β-turn structure formation. The latter is not surprising, as we observed the same type of order in the α-hydrazino peptide **h(1)** with 2 consecutive β-turns (Figure 5 left), and shows the natural tendency of this part of the sequence toward such structural organization. This pattern could not be observed by analyzing the parent peptide alone. This suggests that by perturbing the properties of the peptide backbone with the insertion of an additional nitrogen atom, we can increase the energy barriers between different favorable conformations, leading to multiple sets of signals on the NMR time scale.

Peptides with N^α^-substituted α-hydrazino acids form hydrazino-turns (Figure S34) and stable eight-membered hydrogen-bonded pseudocycles that have been observed in crystals and in CDCl_3_ solution.^27,28,32,50,51^ The position of α-hydrazino acid substitution in the α-hydrazino peptide **h(8)**- puts the N^α^ atom in a position to form a bifurcated hydrogen bond with the C-terminal amide-protecting group and the carbonyl group of the preceding residue.^46^ Therefore, based on our β-turn structural model of α-hydrazino peptide **h(8)** (Figure 5 right), we hypothesize that the formation of a hydrazino turn is also possible in DMSO-*d*_6_. Salaün et al. proposed the analysis of the chemical shift difference (Δδ) between geminal H_N_ protons (e.g., C-terminal amide-protecting group protons) as a sensor for their involvement in strong hydrogen bonding, as in the hydrazino turn conformation.^51^ However, due to its transient nature and susceptibility to solvent effects, the Δδ value was found to be dependent on the equilibrium between the solution state with and without hydrazino turn formation.^51^ The respective Δδ values of 0.36 and 0.65 ppm in pure DMSO-*d*_6_ or with 70% v/v water for the α-hydrazino peptide **h(8)** differ from 0.33 and 0.47 ppm for the parent peptide under the same conditions (Figure S37). From this, we can conclude that the proton of the C-terminal amide-protecting group of α-hydrazino peptide **h(8)** is involved in a stronger hydrogen bond in α-hydrazino peptide **h(8)** compared to the parent peptide. Therefore, we support the hypothesis that hydrazino-turns can also be formed in DMSO-*d*_6_.

Since we could not propose the structural model for the minor E-form of the α-hydrazino peptide **h(8)**, we could not confirm whether the structuring in the form of hydrazino-turn or γ-turn is the actual cause of the shift in E/Z equilibrium toward the Z-isomer, observed previously by Acherar et al.^31^ This is certainly not the case for α-hydrazino peptide **h(4)**, for which the E-isomer predominates despite the formation of a stable α-turn in the Z form. Interestingly enough, we observed structural ordering in the form of protein-like turns only in the Z-isomer conformation, indicating that this spatial arrangement is more favorable for peptide main-chain turning according to the ordering tendencies of the parent sequence.

### Multiple-Site Hydrazino Acid Modifications Show Greater Structural Heterogeneity

In order to increase the conformational space of α-hydrazino peptides as potential peptidomimetics, we prepared α-hydrazino peptide **h(1,4,8)** with a combination of modifications. Its in-depth NMR analysis was not possible due to severe signal overlap. However, a comparison of its proton spectra with those of all other samples points to the conclusion that the same hydrazidic and H_Nα_ nuclei are located in similar chemical environments when present at the same sequence sites in mono- and trisubstituted α-hydrazino peptides (Figure S27). In addition, we observed signal patterns in 2D NMR spectra similar to those of single-site modifications, possibly indicating a similar structural organization of α-hydrazino peptide **h(1,4,8)**. This allows us to suggest its properties based on the results of single-site substituted peptidomimetics.

It is hypothesized that structurally restricted peptides with ligand-binding conformations are preferred for binding to their receptors in solution due to their preorganization, which lowers the unfavorable entropic cost of binding. Furthermore, the addition of new hydrogen bond donors and acceptors leads to stronger binding interactions and thus increases their enthalpy contribution.^52,53^ Therefore, a combination of studied hydrazino modifications in α-hydrazino peptide **h(1,4,8)** could lead to helical backbone preorganization, which is required for more effective binding to the MDM2 surface in potential tumor treatment.

## Conclusions

Peptides with α-hydrazino acids and their N^α^-substituted analogues were shown to form specific higher-order structure motifs.^27,50,51^ Acherar et al. undertook in-depth secondary structure analysis of oligomers composed of alternating α-amino acids and α-hydrazino acids and reported that the major conformer is present in equilibrium between the pseudospiranic and hydrazino-turn conformation.^31^ Furthermore, the CD spectroscopy study of the terminally protected α-hydrazino acid hexamer composed of hLeu and hAla revealed similar structural characteristics compared to β-peptides, such as the presence of right-handed helical structure.^32^ However, detailed insight into the equilibrium of such α-hydrazino peptides’ conformational properties by NMR spectroscopy was hampered by severe resonance signal overlap in the amide proton region.

In this study, we applied the concept of α-hydrazino peptides inspired by the interaction between p53 and MDM2, which is essential for the development of numerous human tumors. The minimal p53-derived sequence with high affinity for binding to MDM2^44^ showed some local organization in DMSO-*d*_6_ in the form of a γ-turn at the C-terminus but no overall higher-order structure. This is consistent with the general observation that unmodified peptides with fewer than 20 residues rarely form higher-order structures due to their high flexibility and conformational freedom in solution. As a result, spectroscopically, we tend to observe the ensemble average of the population of rapidly changing conformations.^54^

By systematically substituting “hot-spot” amino acids with their α-hydrazino acid analogues, we were able to introduce stereoelectronic restraints on the backbone to increase the propensity for local structural organization and folding. 3D models of α-hydrazino peptides **h(1)**, **h(4)**, and **h(8)** with single-site modifications revealed the effect of an additional sp^3^-hybridized nitrogen atom, which causes rotation of the backbone by rearranging hydrogen bond network. Ultimately, this leads to peptide structuring in the form of a γ-, β-, hydrazino-, or α-turn, which could play an important role in various biological ligand–receptor interactions, such as binding to G-protein coupled receptors^22,55−58^ or integrin recognition.^23^ This observation confirms the use of this modification in the *de novo* design of various receptor inhibitors. We also observed an isomerization of the E/Z hydrazide bond in solution due to the introduction of the atypical hydrazine group into the backbone. This further increases the adaptability by expanding the conformational space of α-hydrazino peptides as potential protein–protein interaction antagonists.

Our work introduces α-hydrazino acid modification as a means to achieve local preorganization, which is often crucial for the efficacy of novel protein–protein inhibitors. Further research and ligand-binding studies are required to confirm this hypothesis and gain deeper insights into the kinetics and thermodynamics of α-hydrazino peptide folding studies, which together with *in silico* mutagenesis, and docking studies could provide us with additional tools to address the larger problem of protein folding.

## Data Sets and Methods

### Peptide Synthesis and Characterization

α-Hydrazino acids and their Fmoc derivatives were synthesized following published procedures with the details provided in the Supporting Information (Figures S1–S25 and Tables S1, S3, S5, S7, and S9). α-Hydrazino peptides were synthesized by solid-phase methods. The peptide chain assembly was made on an automated synthesizer using a Rink Amide MBHA resin (0.59 mmol/g; 0.06 mmol). Standard Fmoc-chemistry was used throughout with a 3-molar excess of the acylating amino acids (details provided in the Supporting Information). Couplings were performed in the presence of *N*,*N*,*N*′,*N*′-tetramethyl-*O-*(1*H*-benzotriazol-1-yl)uronium hexafluorophosphate, *O*-(benzotriazol-1-yl)-*N*,*N*,*N*′,*N*′-tetramethyluronium hexafluorophosphate (HBTU), or 1-[bis(dimethylamino)methylene]-1*H*-1,2,3-triazolo[4,5-*b*]pyridinium 3-oxide hexafluorophosphate (HATU, for α-hydrazino acids) 1-hydroxy-benzotriazole (HOBt) and *N*-methyl morpholine (NMM). The reaction was carried out in *N*,*N*-dimethylformamide (DMF). Fmoc group was cleaved with 20% piperidine in DMF, while peptides were cleaved from the solid support with trifluoroacetic acid (TFA, 92.5%) in the presence of triisopropylsilane (TIPS, 2.5%), ethane-1,2-dithiol (EDT, 2.5%), and H_2_O (2.5%) as scavengers. Peptides were precipitated in cold diisopropyl ether and purified on an RP-HPLC Varian 940 LC, with a photodiode array detector on preparative column Phenomenex Luna C18 (21.2 mm × 250 mm, flow 10 mL/min). Product purity was monitored on an analytical column Phenomenex Luna C18 (5 μm, 4.6 mm × 250 mm, flow 0.5 mL/min), at wavelengths 215 and 280 nm. High-resolution mass spectrometry (HRMS) analysis was performed by a nanoUPLC-ESI-qTOF on a nanoAcquity Ultra Performance LC spectrometer (Waters) operating in positive ionization mode. Fluorescence spectra were recorded on a Varian Cary Eclipse fluorimeter in quartz cuvettes (1 cm). CD spectra of parent peptide and α-hydrazino peptides were recorded from a 1 × 10^–5^ M solution in water on a JASCO J815 spectrophotometer at room temperature using 0.1 cm path quartz cuvettes with a scanning speed of 200 nm min^–1^. The water background was subtracted from each spectrum, while each spectrum was a result of three accumulations.

### NMR Spectroscopy

All NMR experiments were performed on Agilent-Varian NMR Systems 800 MHz spectrometers equipped with a triple ^1^H/^13^C/^15^N resonance cryogenic probe head with inverse detection at 298 K unless noted otherwise. Unlabeled α-hydrazino peptides were dissolved in DMSO-*d*_6_ (Armar Chemicals) at 2 mM concentration. For backbone and side-chain ^1^H as well as ^13^C atom assignments, standard double resonance NMR experiments,^59^ such as ^1^H–^1^H TOCSY, ^1^H–^1^H ROESY, ^1^H–^13^C HSQC, ^1^H–^15^N HSQC, and ^1^H–^13^C HMBC, were recorded (Figures S37–S47). Dbppste^60^ diffusion experiment was preformed using 20 different gradient strengths (2.4–60 G cm^–1^). The complete list of chemical shifts is available in Table S2 for the parent peptide, Table S4 for α-hydrazino peptide **h(1)**, Table S6 for α-hydrazino peptide **h(4)**, Table S8 for α-hydrazino peptide **h(8)**, and Table S10 for α-hydrazino peptide **h(1,4,8)**. Assignments were followed by extraction of NOE spatial restraints from the ^1^H–^1^H NOESY experiment with a mixing time of 150 ms. All spectra were processed by NMRPipe^61^ and analyzed with Sparky (UCSF).^62^

In order to gain a cumulative effect of the substitution on the local chemical environment, we performed chemical shift perturbation analysis, used in previous studies.^63,64^ The Δδ_*i*_ values were calculated as a difference of backbone atom chemical shift observed in the α-hydrazino peptide NMR spectra subtracted by the value for the parent peptide. The numerical values represent shift scaling factors, determined from the ratio of the average variance of particular backbone nuclei and amidic proton chemical shifts observed for the 20 amino acid residues in proteins deposited in the BMRB database,^65^ as previously described.^64^Chemical shift perturbation analysis:
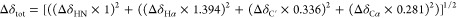
1

### Structure Calculation

The structures of α-hydrazino peptides were calculated by the simulated annealing (SA) simulations based on NOE-derived distance restraints. NOE cross-peaks were classified as strong (1.8–3.6 Å), medium (2.6–5.0 Å), and weak (3.5–6.5 Å). SA simulations were performed using the CUDA version of pmemd module of AMBER 14 program suites^49,66^ and ff14SB force field.^67^ The partial charges of α-hydrazino acid residue were generated by geometry optimization and electrostatic potential (ESP) calculations with Gaussian 09^68^ at the level of HF/6-31G*, followed by RESP fitting^69^ via antechamber^70^ module of AmberTool 15.^49^ Other missing force field parameters were adopted from the Generalized Amber force field.^71^ The initial extended single-stranded peptide structure was obtained using the leap module of AMBER 14. A total of 100 structures were calculated in 80 ps of NMR-restrained simulated annealing (SA) simulations using the generalized Born implicit model.^72,73^ The cutoff for nonbonded interactions was 999 Å, and the SHAKE algorithm^74^ for hydrogen atoms was used with the 0.4 fs time steps. For each SA simulation, a random velocity was used. The SA simulation was as follows: in 0–2 ps, the temperature was raised from 300 to 1000 K and held constant at 1000 K for 38 ps. Temperature was scaled down to 500 K in the next 24 ps, reduced to 100 K in the next 8 ps, and further reduced to 0 K in the last 8 ps. NOE-derived distance restraints (force constant 20 kcal mol^–1^ Å^–2^) were used in the calculation. Ten structures with the smallest restraints violations and lowest energy were further refined in explicit DMSO solvent at 300 K with NOE-derived distance restraints for 10 ns and were minimized with a maximum of 5000 steps of energy minimization. The energy of each structure was calculated in the generalized Born implicit model with a solvent dielectric constant of 46.45.

## Data Availability

The data sets generated and analyzed during the current study are available from the corresponding author on reasonable request considering that these results are unpublished data from another study being considered for a patent application.

## Supporting Information Available

Details of peptide synthesis and chemical characterization (mass spectrometry, fluorescence, and CD spectra), NMR assignment data, NMR restraints and structural statistics, and comparison of 1D NMR spectra of all α-hydrazino peptides with respect to the parent peptide, chemical shift perturbation, ROESY spectra of α-hydrazino peptides, DOSY spectrum of α-hydrazino peptide **h(4)**, factors affecting E/Z isomerism in α-hydrazino peptides, conformational equilibrium for the major Z conformer, NOESY spectrum of **h(8)**, chemical shift differences of major and minor forms of α-hydrazino peptides, and comparison of ^1^H NMR spectra of parent peptide in DMSO-*d*_6_/H_2_O mixtures (PDF)
