# Supplementary material for: α-Hydrazino Acid Insertion Governs Peptide Organization in Solution by Local Structure Ordering
Source: ACS Omega. 2024 May 8;9(20):22175–85. doi: 10.1021/acsomega.4c00804 (PMC11112695; doi:10.1021/acsomega.4c00804)
Supplement: Supplementary file 1 — ao4c00804_si_001.pdf [file ao4c00804_si_001.pdf]

Supporting information

α-Hydrazino acid insertion governs peptide organization in solution by local structure ordering

Luka Kavčič,[a] Gregor Ilc,[a,b,#] Baifan Wang,[a,#] Kristina Vlahoviček-Kahlina,[c,#] Ivanka Jerić,[c] Janez Plavec [a,b,d,*]

[a] Slovenian NMR Centre, National Institute of Chemistry, Ljubljana, Slovenia
[b] EN-FIST Centre of excellence, Ljubljana, Slovenia
[c] Division of Organic Chemistry and Biochemistry, Rudjer Bošković Institute, Zagreb, Croatia
[d] Faculty of Chemistry and Chemical Technology, University of Ljubljana, Ljubljana, Slovenia
* Corresponding author, email: janez.plavec@ki.si

[#] Current affiliations:
Gregor Ilc, Krka pharmaceuticals, Novo mesto, Slovenia
Baifan Wang, College of Chemistry, Nankai University, Tianjin, China
Kristina Vlahoviček-Kahlina, Faculty of Agriculture, University of Zagreb, Zagreb, Croatia

Materials and methods

Unless otherwise indicated, solvents were used as supplied (analytical or HPLC grade) without further purification. Reagents were used directly as supplied by major chemical suppliers. Synthesis of α-hydrazino acids was monitored by analytical thin-layer chromatography (TLC) performed on Merck Kieselgel 60 F254 0.25 mm precoated aluminum plates. After elution, the plate was visualized under UV illumination at 254 nm for UV- active materials. Further visualization was achieved by staining with ammonium molybdate or ninhydrin solution and charring on a hot plate. Melting points were determined using an Electrothermal 9100 apparatus in open capillaries and are uncorrected. The peptide chain assembly was made on an automated synthesizer using Rink Amide MBHA resin. Peptides were purified on RP-HPLC Varian 940 LC, with Photodiode Array detector on preparative column Phenomenex Luna C18 (21.2 × 250 mm, flow 10 mL/min). Product purity was monitored on analytical column Phenomenex Luna C18 (5 μm, 4.6 × 250 mm, flow 0.5 mL/min), at wavelengths 215 and 280 nm. High resolution mass spectrometry (HRMS) analysis was performed by a nanoUPLC-ESI-qTOF on nanoAcquity Ultra Performance LC spectrometer (Waters, USA) operating in in positive ionization mode. Fluorescence spectra were recorded on a Varian Cary Eclipse fluorimeter in quartz cuvettes (1 cm). CD spectra were on a JASCO J815 spectrophotometer at room temperature using 0.1 cm path quartz cuvettes, with a scanning speed of 200 nm min−1. The water background was subtracted from each spectrum, while each spectrum was a result of three accumulations. All NMR experiments were performed on Agilent-Varian NMR Systems 800 MHz spectrometers equipped with triple $^{1}H/^{13}C/^{15}N$ resonance cryogenic probe head with inverse detection at 298 K unless noted otherwise. Unlabelled α-hydrazino peptides were dissolved in DMSO-d_6 (Armar Chemicals) at 2mM concentration. All spectra were processed by NMRPipe[1] and analysed with Sparky (UCSF)[2].

Peptide synthesis

Synthesis of α-hydrazino acids

α-Hydrazino acids were prepared according the previously published procedure[3], with slight modifications.[4] Briefly, D-amino acid was dissolved in 2.5 M H_2SO_4 (1.3 mL/mmol) and KBr (3.5 equiv.) was added. The solution was cooled down to 0°C and then the solution of $NaNO_2$ (1.3 equiv.) was added dropwise. After 1 h at 0 °C, the reaction mixture was stirred at room temperature overnight. α-Bromo acid was extracted with EtOAc, washed with $NaHCO_3$ and dried over $MgSO_4$. After evaporation, product was obtained as yellow oil. Obtained α-bromo acid was dissolved in EtOH (2 mL) and added dropwise to a solution of hydrazine hydrate (3 equiv.) in EtOH (1 mL). The reaction mixture was irradiated under MW at 70 °C and 50 W for 1 h. A white suspension was obtained. Solvent was evaporated, and product recrystallized from EtOH/diethyl ether. Optical purity of prepared α-hydrazino acids has been checked by the chiral thin-layer chromatography and confirmed complete conversion.

[1] Delaglio, F.; Grzesiek, S.; Vuister, G.; Zhu, G.; Pfeifer, J.; Bax, A. NMRPipe: A Multidimensional Spectral Processing System Based on UNIX Pipes. *J. Biomol. NMR* **1995**, *6* (3), 277–293

[2] Goddard, Td.; Kneller, D. G. Sparky 3. *University of California, San Francisco*. University of California: San Francisco **2004**, p. 15

[3] Panda, S.S.; El-Nachef, C.; Bajaj, K.; Katritzky, A.R. Syntheses of hydrazino peptides and conjugates. *Eur. J. Org. Chem.* 2013, 19, 4156–4162. doi:10.1002/ ejoc.201201731

[4] Suć, J.; Jerić I. Synthesis of hybrid hydrazino peptides: protected vs unprotected chiral α-hydrazino acids. SpringerPlus (2015) 4:507

NH_2-NH-Leu-OH (yield 40%):[5] white powder, R_f = 0.21 (EtOAc:EtOH:AcOH:H_2O=7:1:1:1), m.p. = 215 °C. ^{1}H NMR (D_2O, 600 MHz): δ 3.64 (t, J = 6.8 Hz, 1H), 1.78–1.59 (m, 3H), 0.93 (d, J = 6.3 Hz, 6H). ^{13}C NMR (D_2O, 600 MHz): δ 176.4, 65.1, 25.9, 23.4, 23.3.

NH_2-NH-Phe-OH (yield 40%):[3] white powder, R_f = 0.23 (EtOAc:EtOH:AcOH:H_2O=7:1:1:1), m.p. = 195 °C. ^{1}H NMR (D_2O, 600 MHz): δ 7.42–7.18 (m, 5H), 3.88 (t, J = 6,3 Hz, 1H), 3.19 (m, 1H), 3.11 (m, 1H). ^{13}C NMR (D_2O, 600 MHz): δ 179.1, 135.7, 132.1, 131.5, 131.2, 64.3, 38.3.

NH_2-NH-Tyr(*t*-Bu)-OH (yield 25%): colourless oil, R_f = 0.33 (EtOAc:EtOH:AcOH:H_2O=7:1:1:1). ^{1}H NMR (D_2O, 600 MHz): δ 7.27 (br d, 2H), 7.05 (br d, 2H), 3.95 (m, 1H), 3.52 (m, 1H), 3.18 (m, 1H), 1.47 (s, 9H). ^{13}C NMR (D_2O, 600 MHz): δ 171.9, 155.6, 132.5, 128.1, 120.2, 77.5, 67.3, 31.6, 27.8.

Synthesis of Fmoc-α-hydrazino acids

Fmoc-group was introduced following a well-established procedure.[6] Briefly, α-hydrazino acid was dissolved in water, pH was adjusted to 9.5 and solution cooled down in an ice bath. Fmoc-OSu (1 equiv.) was dissolved in acetonitrile and added dropwise to the solution of α-hydrazino acid. pH was monitored and kept at 8.5-9 (1M NaOH). After 3 h solvent was evaporated, product extracted with EtOAc and crystallized from EtOAc/hexane.

Fmoc-hLeu-OH (yield 75%): white powder, R_f = 0.34 (DCM-MeOH=9:1), m.p. = 175-180 °C. ^{1}H NMR ($CDCl_3$, 600 MHz): δ 9.80 (s, 1H), 7.83 – 7.71 (m, 2H), 7.51 – 7.47 (m, 2H), 7.44 – 7.38 (m, 2H), 7.36 – 7.29 (m, 2H), 5.48 (br s, 1H), 4.70 (br d, 2H), 4.48 – 4.43 (m, 1H), 4.44 (br s, 1H), 3.80 – 3.77 (m, 1H), 2.03 – 1.97 (m, 1H), 1.73 (d, J = 13.8 Hz, 2H), 0.99 (d, J = 6.3 Hz, 6H). ^{13}C NMR ($CDCl_3$, 600 MHz) δ 175.1, 156.6, 144.8, 143.9, 139.2, 139.8, 128.6, 128.3, 127.4, 127.9, 124.7, 124.4, 122.8, 122.5, 66.7, 64.8, 48.2, 39.2, 25.4, 23.2.

Fmoc-hPhe-OH (yield 83%): white powder, R_f = 0.45 (DCM-MeOH=9:1), m.p. = 158-162 °C. ^{1}H NMR ($CDCl_3$, 600 MHz) δ 9.78 (s, 1H), 7.70 – 7.69 (m, 2H), 7.60 – 7.12 (m, 11H), 5.44 (br s, 1H), 4.76 – 4.64 (m, 2H), 4.46 (br s, 1H), 4.14 – 4.07 (m, 1H), 3.36 (s, 1H), 3.26 (br s, 1H), 2.98 – 2.90 (m, 1H). ^{13}C NMR ($CDCl_3$) δ 172.8, 156.6, 144.8, 144.7, 139.6, 139.3, 137.5, 129.4, 129.3, 129.1, 128.6, 127.4, 127.2, 127.1, 124.2, 122.8, 67.4, 66.9, 48.1, 32.2.

Fmoc-hTyr(*t*Bu)-OH (yield 33% after purification by column chromatography and crystallization): white powder, R_f = 0.32 (DCM-MeOH=9:1, m.p. = 145-153 °C. ^{1}H NMR ($CDCl_3$, 600 MHz) δ 9.40 (s, 1H), 7.81 – 7.69 (m, 2H), 7.49 – 7.28 (m, 6H), 7.19 – 7.10 (m, 2H), 6.93 – 6.83 (m, 2H), 5.64 (s, 1H), 4.72 – 4.67 (m, 2H), 4.51 – 4.45 (m, 1H), 4.12 – 4.04 (m, 1H), 3.65 (s, 1H), 3.20 – 3.14 (m, 1H), 3.05 – 3.92 (m, 1H), 1.63 – 1.30 (s, 9H). ^{13}C NMR ($CDCl_3$, 600 MHz) δ 172.6, 156.5, 156.1, 144.8, 144.6, 139.3, 132.8, 128.6, 128.4, 127.8, 127.4, 127.4, 124.4, 124.2, 122.6, 120.0, 119.8, 77.5, 67.1, 66.5, 47.8, 32.0, 27.7, 27.4, 27.3.

[5] All data consistent with published data (reference 1).
[6] Methods of organic chemistry – Synthesis of peptides and peptidomimetics (Huben Weyl, 2005, M. Goodman Ed.) Podlech, J.; Gurrath, M.; Müller, G. vol E22c, p39-425.

Synthesis of parent peptide and α-hydrazino peptides

General procedure: The synthesis was done on the basis of 0.06 mmol. N-terminally protected amino acid each (0.18 mmol) and the reagents *O*-benzotriazol-1-yl-1,1,3,3-tetramethylammonium hexafluorophosphate (HBTU, 0.18 mmol) and 1-hydroxybenzotriazole (HOBt, 0, 18 mmol) were weighed into the vials and each amino acid was placed on the appropriate slot on the instrument. Rink Amide MBHA resin (0.59 mmol/g; 100 mg) was used for the synthesis. The reaction was carried out in *N*,*N*-dimethylformamide (DMF). Each step of the automatic synthesis of peptides on a solid support is regulated by the appropriate program listed in Table. Deprotection of the Fmoc protecting group was carried out with a 20 % solution of piperidine in DMF, and activation with a 0.4 M solution of *N*-methylmorpholine (NMM) in DMF. Deprotection and cleavage of the resin were performed with a mixture composed of trifluoroacetic acid/triisopropylsilane/water/ethane-1,2-dithiol (TFA/TIS//H_2O/EDT: 92.5/2.5/2.5/2.5 (v/v, 3 mL, 3 h). The peptide was precipitated in cold diisopropyl ether, and purified on RP-HPLC Varian 940 LC, with Photodiode Array detector on preparative column Phenomenex Luna C18 (21.2 × 250 mm, flow 10 mL/min). Product purity was monitored on analytical column Phenomenex Luna C18 (5 µm, 4.6 × 250 mm, flow 0.5 mL/min), at wavelengths 215 and 280 nm. The following solvent mixtures were used for RP-HPLC chromatography:

0→5 min	10 % MeCN / 0.1 TFA → 55 % MeCN / 0.1 TFA
5→20 min	55 % MeCN / 0.1 TFA
20→25 min	55 % MeCN / 0.1 TFA → 100 % MeCN / 0.1 % TFA

Parent peptide FMDYWEGL

Yield: 77 % (49 mg). tR = 11.52 min. HRMS: calculated for C51H66N10O13S [M+H]+ 1059.4610; found 1059.4631

Table S1. Amino acids, reagents and applied programs for synthesis on a solid support, 1. – introduction of the first amino acid, 2. – introduction of others, and 3. – removal of Fmoc group

Program	Sample no.	Amino acids and reagents	Mass (mg)
1	1	Fmoc-Leu-OH<br>HBTU<br>HOBt	63.61<br>68.27<br>24.32
2	2	Fmoc-Gly-OH<br>HBTU<br>HOBt	53.52<br>68.27<br>24.32
2	3	Fmoc-Glu(OtBu)-OH<br>HBTU<br>HOBt	76.59<br>68.27<br>24.32
2	4	Fmoc-Trp(Boc)-OH<br>HBTU<br>HOBt	94.78<br>68.27<br>24.32
2	5	Fmoc-Tyr(tBu)-OH<br>HBTU<br>HOBt	82.72<br>68.27<br>24.32
2	6	Fmoc-Asp(OtBu)-OH<br>HBTU<br>HOBt	74.06<br>68.27<br>24.32
2	7	Fmoc-Met-OH<br>HBTU<br>HOBt	66.86<br>68.27<br>24.32
2	8	Fmoc-Phe-OH<br>HBTU<br>HOBt	69.73<br>68.27<br>24.32
3	9	-	-

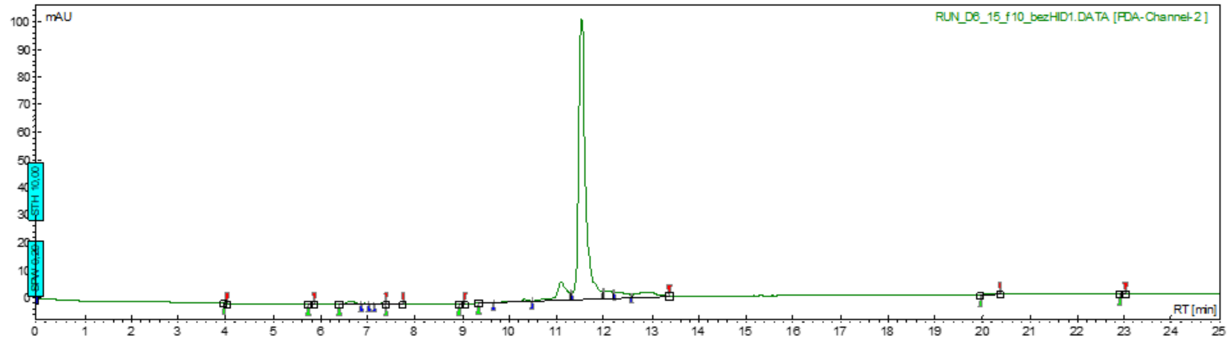

Figure S1. HPLC-DAD chromatogram of purified parent peptide. HPLC conditions given in General procedure.

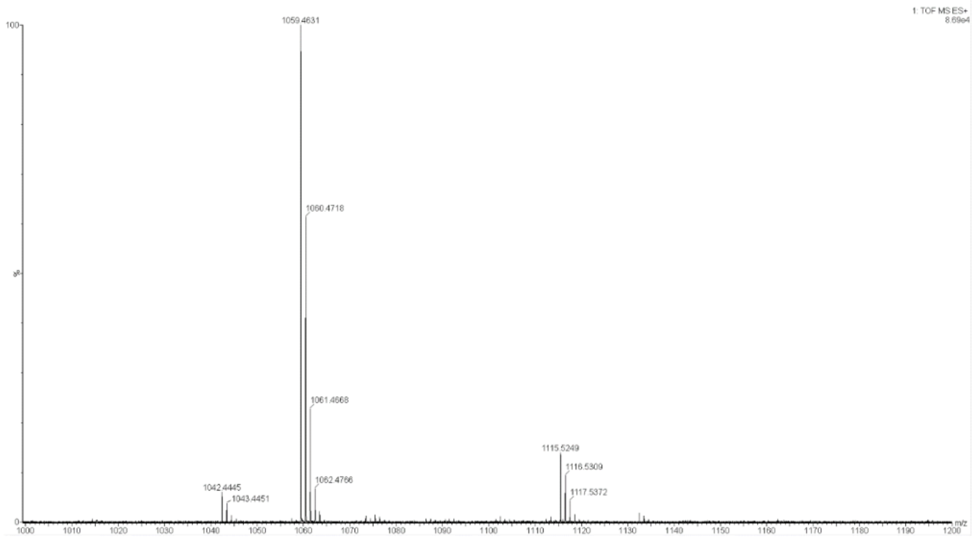

Figure S2. HRMS spectrum of the parent peptide.

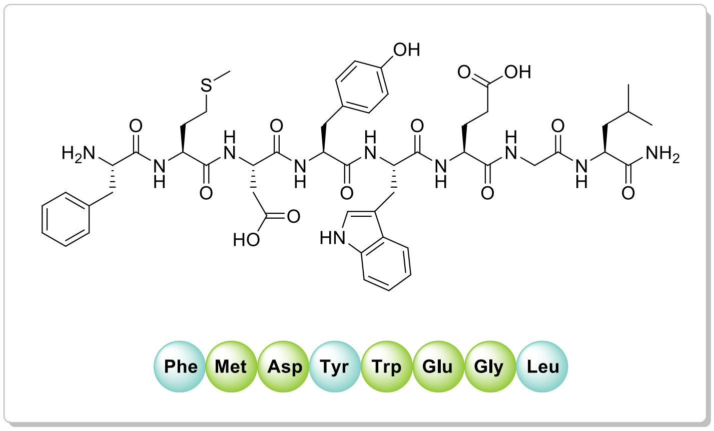

Figure S3. Chemical structure of the parent peptide.

Table S2. Chemical shift data for the parent peptide

Group	Atom	Nucleus	Chemical shift
Nt	HN1	1H	7.33
Nt	HN2	1H	6.99
F1	HA	1H	4.06
F1	HB2	1H	2.90
F1	HB3	1H	3.08
F1	HD	1H	7.23
F1	HE	1H	7.33
F1	HN	1H	8.06
F1	HZ	1H	7.27
M2	HA	1H	4.43
M2	HB2	1H	1.74
M2	HB3	1H	1.90
M2	HE	1H	2.00
M2	HG1	1H	2.42
M2	HG2	1H	2.45
M2	HN	1H	8.68
D3	HA	1H	4.55
D3	HB2	1H	2.45
D3	HB3	1H	2.67
D3	HN	1H	8.34
Y4	HA	1H	4.38
Y4	HB2	1H	2.65
Y4	HB3	1H	2.83
Y4	HD	1H	6.94
Y4	HE	1H	6.59
Y4	HH	1H	9.14
Y4	HN	1H	7.82
W5	HA	1H	4.54
W5	HB2	1H	2.96
W5	HB3	1H	3.15
W5	HD1	1H	7.13
W5	HE1	1H	10.75
W5	HE3	1H	7.58
W5	HH2	1H	7.04
W5	HN	1H	8.11
W5	HZ2	1H	7.31
W5	HZ3	1H	6.97
E6	HA	1H	4.25
E6	HB2	1H	1.76
E6	HB3	1H	1.91
E6	HG1	1H	2.25
E6	HN	1H	8.09
G7	HA	1H	3.70
G7	HN	1H	7.93
L8	HA	1H	4.22
L8	HB	1H	1.46
L8	HD1	1H	0.83
L8	HD2	1H	0.86
L8	HG	1H	1.58
L8	HN	1H	7.84
F1	C	13C	167.7
F1	CA	13C	53.2
F1	CB	13C	36.8
F1	CD	13C	129.3
F1	CE	13C	128.4
F1	CZ	13C	126.9
M2	C	13C	170.2
M2	CA	13C	51.6
M2	CB	13C	32.4
M2	CE	13C	14.4
M2	CG	13C	29.1
D3	C	13C	170.2
D3	CA	13C	49.4
D3	CB	13C	35.9
D3	CO	13C	171.7
Y4	C	13C	170.8
Y4	CA	13C	54.1
Y4	CB	13C	36.5
Y4	CD	13C	129.9
Y4	CE	13C	114.7
W5	C	13C	171.4
W5	CA	13C	53.4
W5	CB	13C	27.3
W5	CD1	13C	123.5
W5	CE3	13C	118.3
W5	CH2	13C	120.6
W5	CZ2	13C	111.1
W5	CZ3	13C	118.0
E6	C	13C	171.3
E6	CA	13C	52.2

E6	CB	13C	27.1	D3	HB3	1H	2.69
E6	CG	13C	29.9	D3	HN	1H	8.49
E6	CO	13C	174.0	E6	HA	1H	4.26
G7	C	13C	168.4	E6	HB2	1H	1.77
G7	CA	13C	41.9	E6	HB3	1H	1.92
L8	C	13C	174.1	E6	HG1	1H	2.27
L8	CA	13C	50.8	E6	HN	1H	8.13
L8	CB	13C	40.8	L8	HA	1H	4.22
L8	CD1	13C	21.5	L8	HN	1H	7.87
L8	CD2	13C	23.0	E6	CG	13C	33.3
L8	CG	13C	24.2				
Nt	N	15N	106.3				
M2	N	15N	121.8				
D3	N	15N	120.0				
Y4	N	15N	117.7				
W5	N	15N	119.9				
W5	NE1	15N	132.6				
E6	N	15N	119.9				
G7	N	15N	107.5				
L8	N	15N	119.8				
	**minor**						
D3	HB2	1H	2.44				

* The chemical shifts were extracted from the NMR spectra recorded at a peptide concentration of 2 mM in DMSO-d_6 and 25 °C using an 800 MHz spectrometer.

Fluorescence spectroscopy

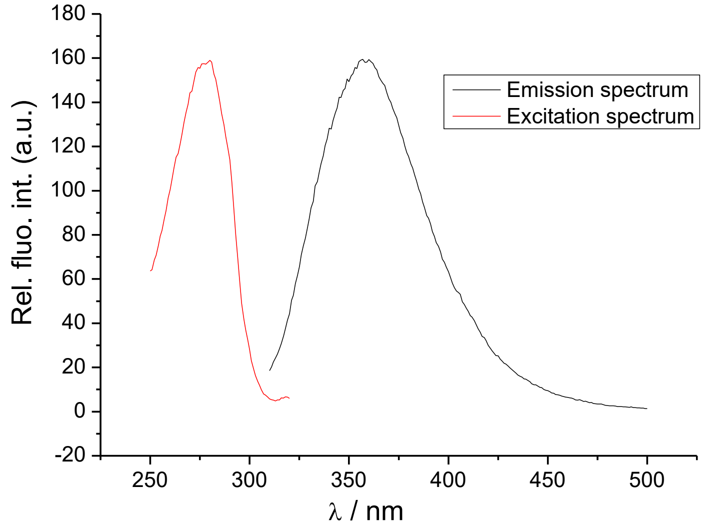

No	c (M)	Abs(max)
1	5,00E-6	159,5
2	1,00E-5	312,6
3	1,50E-5	462,1

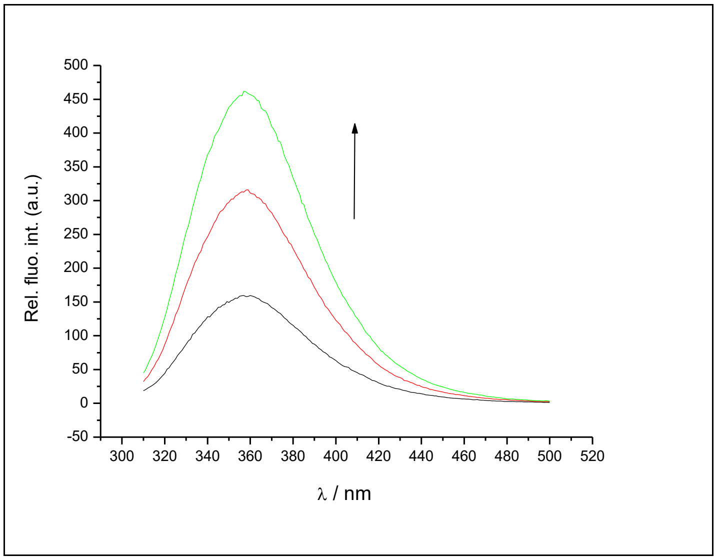

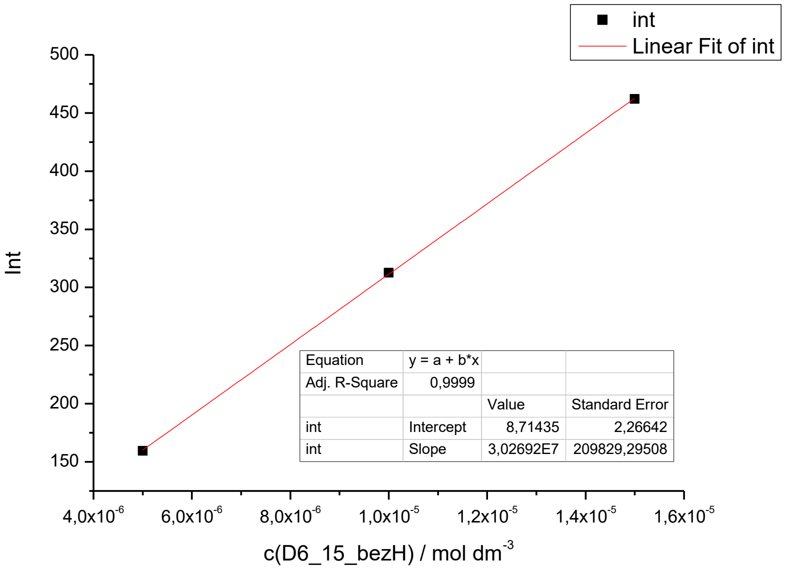

Figure S4. Fluorescence spectra of the parent peptide, λ_{exc} = 280 nm, (up); linear dependence (—) of the fluorescence intensity λ_{exc} = 280 nm, λ_{em} = 356 nm (■) on the parent peptide concentration (down), (Na-cacodylate buffer, I=0.05 M, pH = 7.0, 20 °C).

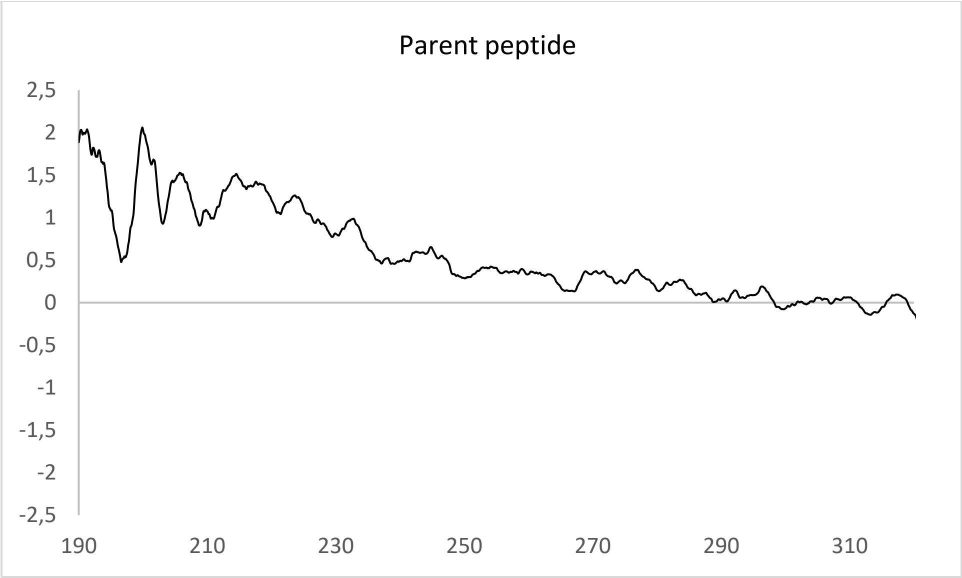

Figure S5. CD spectrum of the parent peptide (1×10^{-5} M solution in water at room temperature).

α-hydrazino peptide h(1):

Yield: 42 % (27 mg). t_R = 11.69 min. HRMS: calculated for $C_{51}H_{67}N_{11}O_{13}S$ $[M+H]^+$ 1074.4719; found 1074.4728.

Table S3. Amino acids, reagents and applied programs for synthesis on a solid support, 1. – introduction of the first amino acid, 2. – introduction of others and 3. – removal of Fmoc group

Program	Sample no.	Amino acids and reagents	Mass (mg)
1	1	Fmoc-Leu-OH<br>HBTU<br>HOBt	63.61<br>68.27<br>24.32
2	2	Fmoc-Gly-OH<br>HBTU<br>HOBt	53.52<br>68.27<br>24.32
2	3	Fmoc-Glu(OtBu)-OH<br>HBTU<br>HOBt	76.59<br>68.27<br>24.32
2	4	Fmoc-Trp(Boc)-OH<br>HBTU<br>HOBt	94.78<br>68.27<br>24.32
2	5	Fmoc-Tyr(tBu)-OH<br>HBTU<br>HOBt	82.72<br>68.27<br>24.32
2	6	Fmoc-Asp(OtBu)-OH<br>HBTU<br>HOBt	74.06<br>68.27<br>24.32
2	7	Fmoc-Met-OH<br>HBTU<br>HOBt	66.86<br>68.27<br>24.32
2	8	**Fmoc-hPhe-OH**<br>HATU<br>HOBt	72.39<br>68.44<br>24.32
4	9	**Fmoc-hPhe-OH**<br>HATU<br>HOBt	72.39<br>68.44<br>24.32
3	10	-	-

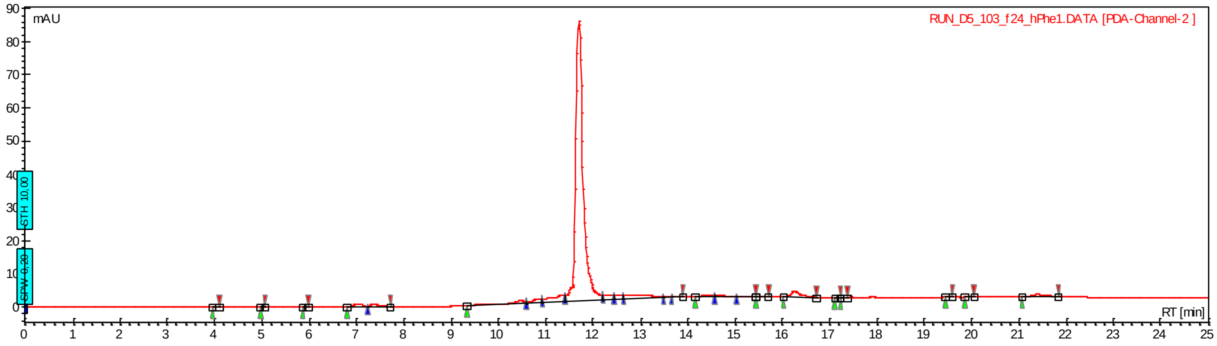

Figure S6. HPLC-DAD chromatogram of purified α-hydrazino peptide **h(1)**. HPLC conditions are given in General procedure.

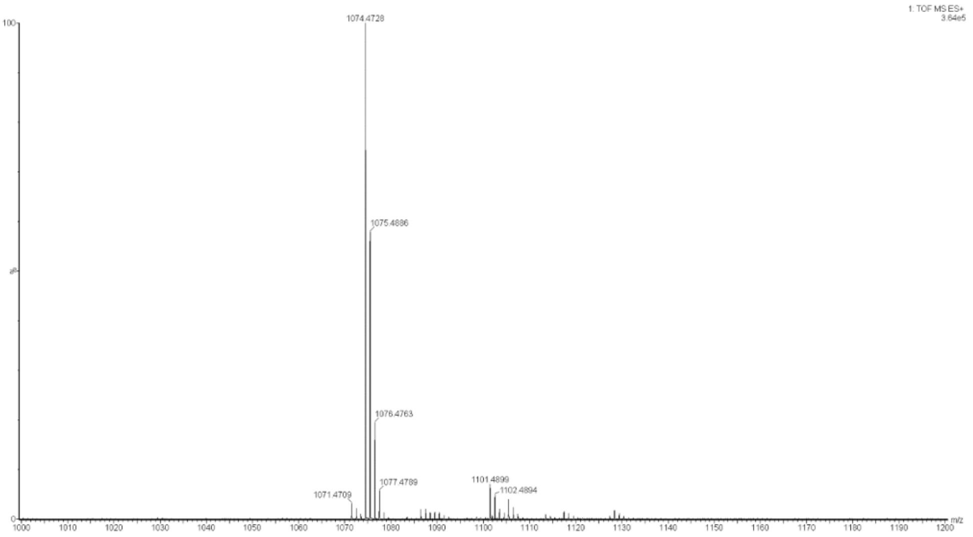

Figure S7. HRMS spectrum of α-hydrazino peptide **(h1)**.

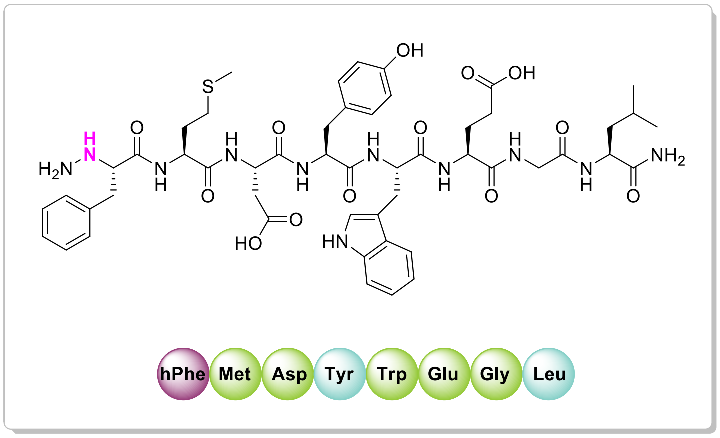

Figure S8. Chemical structure of α-hydrazino peptide **(h1)**.

Table S4. Chemical shift data of α-hydrazino peptide (h1)

Group	Atom	Nucleus	Chemical shift
Nt	HN1	1H	7.33
Nt	HN2	1H	7.00
F1	HNα	1H	5.32
F1	HNβ	1H	8.87
F1	HA	1H	3.78
F1	HB2	1H	2.99
F1	HB3	1H	2.70
F1	HD1	1H	7.24
F1	HE1	1H	7.28
M2	HA	1H	4.40
M2	HB2	1H	1.91
M2	HB3	1H	1.77
M2	HG	1H	2.43
M2	HN	1H	8.55
D3	HA	1H	4.55
D3	HB2	1H	2.67
D3	HB3	1H	2.46
D3	HN	1H	8.28
Y4	HA	1H	4.37
Y4	HB2	1H	2.82
Y4	HB3	1H	2.65
Y4	HD1	1H	6.94
Y4	HE1	1H	6.59
Y4	HH	1H	9.11
Y4	HN	1H	7.83
W5	HA	1H	4.53
W5	HB2	1H	3.14
W5	HB3	1H	2.96
W5	HD1	1H	7.14
W5	HE1	1H	10.76
W5	HE3	1H	7.58
W5	HH2	1H	7.04
W5	HN	1H	8.11
W5	HZ2	1H	7.30
W5	HZ3	1H	6.98
E6	HA	1H	4.24
E6	HB2	1H	1.91
E6	HB3	1H	1.76
E6	HG	1H	2.25
E6	HN	1H	8.10
G7	HA	1H	3.69
G7	HN	1H	7.93
L8	HA	1H	4.22
L8	HB3	1H	1.57
L8	HD1	1H	0.87
L8	HD2	1H	0.82
L8	HG	1H	1.46
L8	HN	1H	7.85
M2	CA	13C	51.6
D3	CA	13C	49.3
Y4	CA	13C	53.9
Y4	CD1	13C	129.8
W5	C	13C	170.5
W5	CA	13C	53.2
E6	CA	13C	52.0
G7	C	13C	169.3
G7	CA	13C	41.8
L8	C	13C	174.0
L8	CA	13C	50.7
Nt	N	15N	106.3
M2	N	15N	120.3
D3	N	15N	119.6
Y4	N	15N	117.7
W5	N	15N	119.9
W5	NE1	15N	133.7
E6	N	15N	119.9
G7	N	15N	107.5
L8	N	15N	119.7
	**minor**		
M2	HA	1H	4.30
M2	HB2	1H	1.82
M2	HB3	1H	1.68
M2	HG	1H	2.31
M2	HN	1H	8.07
Nt	HN1	1H	7.31
D3	HA	1H	4.49
D3	HB2	1H	2.63
D3	HB3	1H	2.45

D3	HN	1H	8.20
Y4	HA	1H	4.33
Y4	HB2	1H	2.81
Y4	HB3	1H	2.66
Y4	HD1	1H	6.91
Y4	HN	1H	7.74
W5	HA	1H	4.50
W5	HB2	1H	3.15
W5	HB3	1H	2.96
W5	HD1	1H	7.14
W5	HN	1H	8.04
E6	HA	1H	4.23
E6	HB2	1H	1.90
E6	HB3	1H	1.77
E6	HG	1H	2.26
E6	HN	1H	8.06
L8	HA	1H	4.20
L8	HN	1H	7.83
M2	CA	13C	51.6
D3	CA	13C	49.5
Y4	CA	13C	54.2
W5	C	13C	170.8
M2	N	15N	122.7
D3	N	15N	118.6
Y4	N	15N	117.4
W5	N	15N	119.6
E6	N	15N	119.8

unassigned or 2nd minor

Y4	HA	1H	4.35
Y4	HN	1H	7.77
W5	HA	1H	4.50
E6	HA	1H	4.24
?	?	1H	7.21

* The chemical shifts were extracted from the NMR spectra recorded at a peptide concentration of 2 mM in DMSO-d_6 and 25 °C using an 800 MHz spectrometer.

Fluorescence spectroscopy

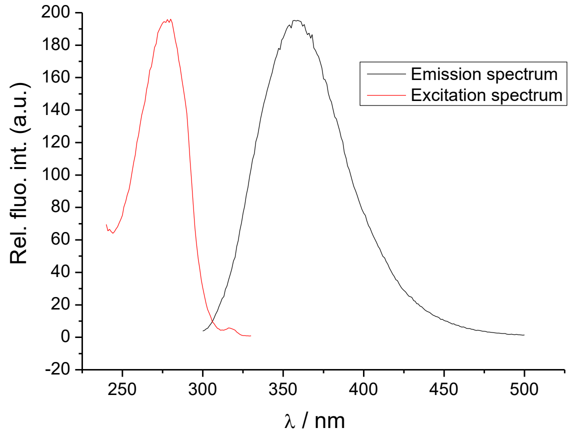

No	c (M)	Abs(max)
1	5×10^{-6}	195.3
2	1×10^{-5}	371.6
3	1.5×10^{-5}	528.0

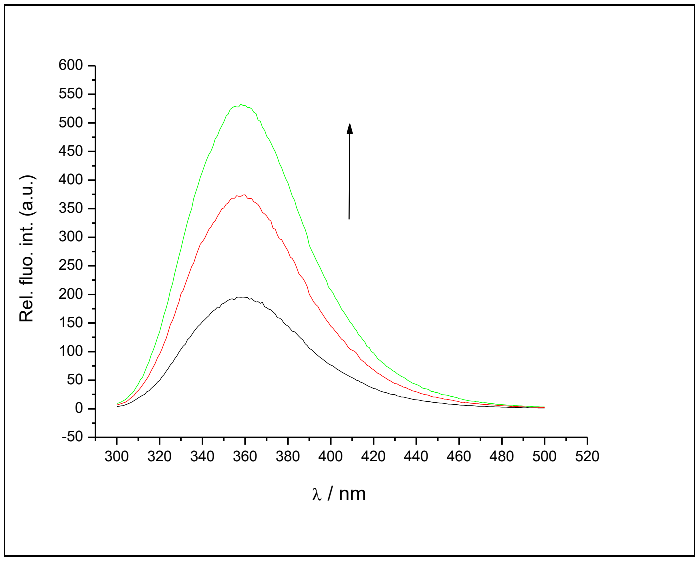

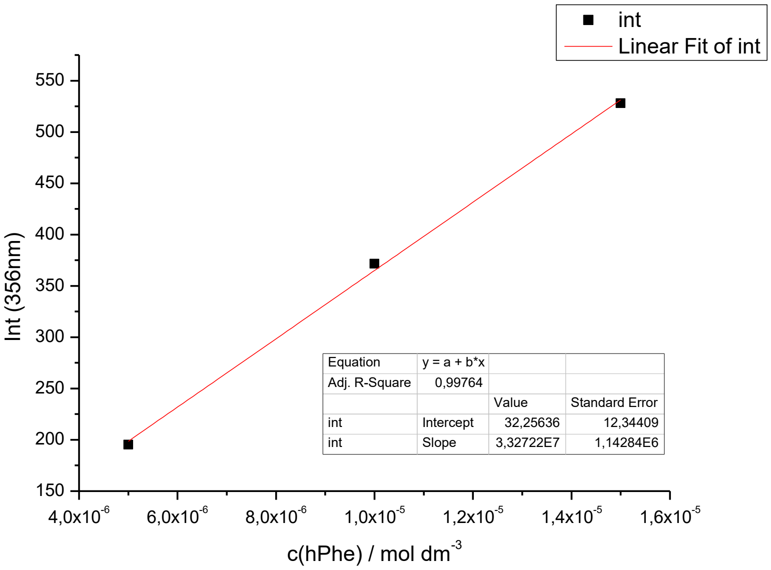

Figure S9. Fluorescence spectra of α-hydrazino peptide **h(1)**, λ_{exc} = 280 nm, (up); linear dependence (—) of the fluorescence intensity λ_{exc} = 280 nm, λ_{em} = 356 nm (■) on the α-hydrazino peptide **h(1)** concentration (middle and down), (Na-cacodylate buffer, I=0.05 M, pH = 7.0, 20 °C).

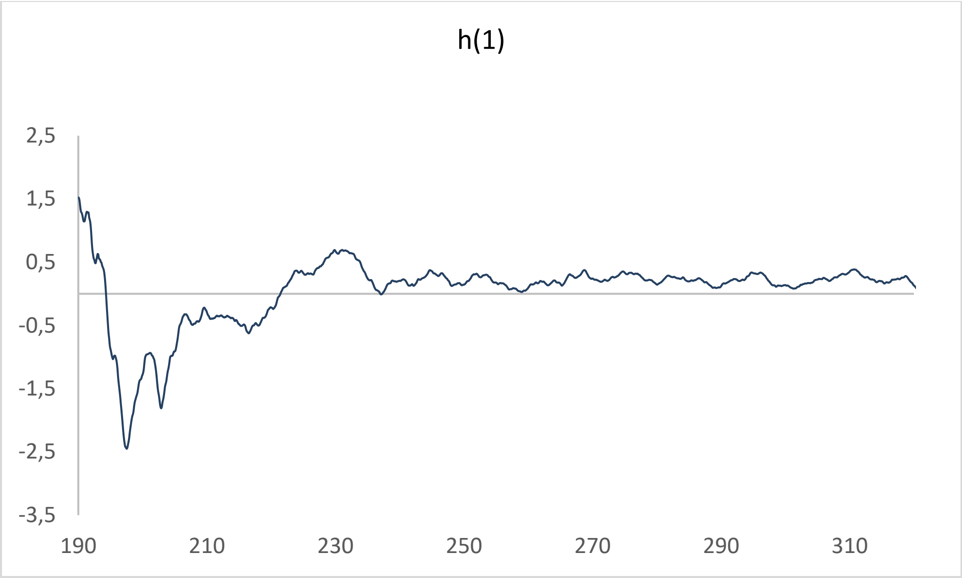

Figure S10. CD spectrum of α-hydrazino peptide **(h1)** (1×10-5 M solution in water at room temperature).

α-hydrazino peptide h(4):

Yield: 46 % (30 mg). t_R = 11.93 min. HRMS: calculated for $C_{51}H_{67}N_{11}O_{13}S$ $[M+H]^+$ 1074.4719; found 1074.4728.

Table S5. Amino acids, reagents and applied programs for synthesis on a solid support, 1. – introduction of the first amino acid, 2. – introduction of others and 3. – removal of Fmoc group

Program	Sample no.	Amino acids and reagents	Mass (mg)
1	1	Fmoc-Leu-OH<br>HBTU<br>HOBt	63.61<br>68.27<br>24.32
2	2	Fmoc-Gly-OH<br>HBTU<br>HOBt	53.52<br>68.27<br>24.32
2	3	Fmoc-Glu(OtBu)-OH<br>HBTU<br>HOBt	76.59<br>68.27<br>24.32
2	4	Fmoc-Trp(Boc)-OH<br>HBTU<br>HOBt	94.78<br>68.27<br>24.32
2	5	**Fmoc-hTyr(tBu)-OH**<br>HATU<br>HOBt	82.72<br>68.44<br>24.32
2	6	Fmoc-Asp(OtBu)-OH<br>HATU<br>HOBt	74.06<br>68.44<br>24.32
2	7	Fmoc-Met-OH<br>HBTU<br>HOBt	66.86<br>68.27<br>24.32
2	8	Fmoc-Phe-OH<br>HBTU<br>HOBt	69.73<br>68.27<br>24.32
3	9	prazna	-

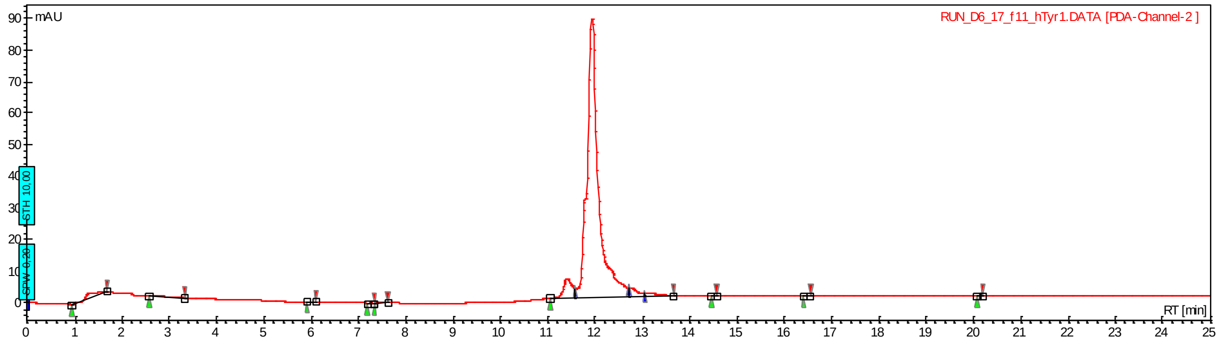

Figure S11. HPLC-DAD chromatogram of purified α-hydrazino peptide **(h4)**. HPLC conditions are given in General procedure.

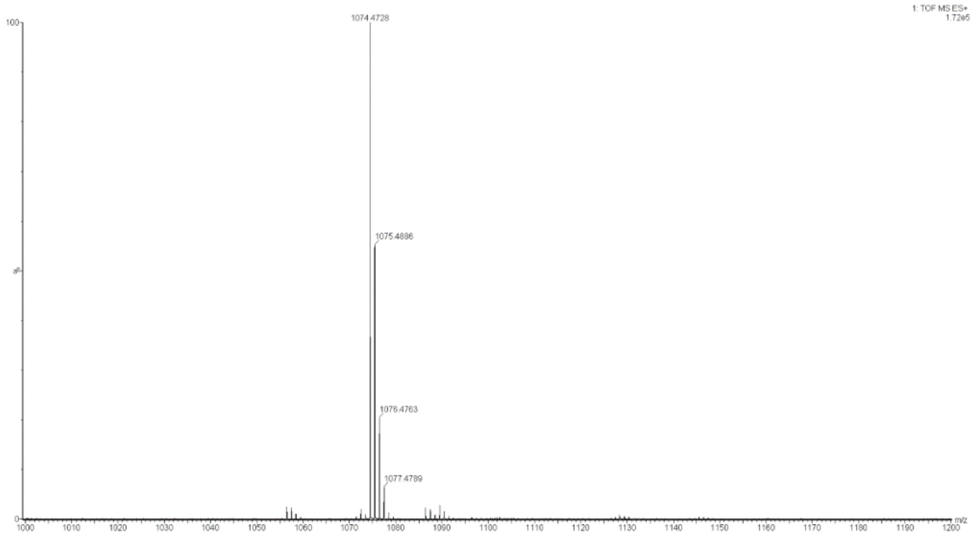

Figure S12. HRMS spectrum of α-hydrazino peptide **(h4)**.

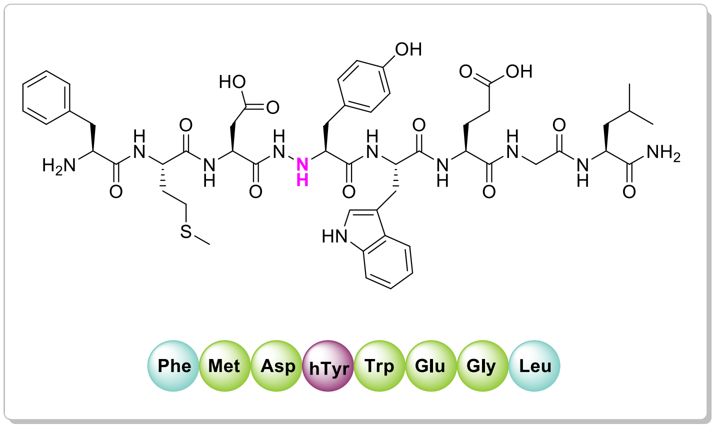

Figure S13. Chemical structure of α-hydrazino peptide **(h4)**.

Table S6. Chemical shift data of α-hydrazino peptide (h4)

Group	Atom	Nucleus	Chemical shift
Nt	HN1	1H	7.33
Nt	HN2	1H	7.01
F1	HA	1H	4.05
F1	HB2	1H	3.09
F1	HB3	1H	2.91
F1	HD1	1H	7.23
F1	HE1	1H	7.33
F1	HN	1H	8.09
F1	HZ	1H	7.27
M2	HA	1H	4.39
M2	HB2	1H	1.80
M2	HB3	1H	1.92
M2	HG	1H	2.44
M2	HN	1H	8.75
D3	HA	1H	4.34
D3	HB2	1H	2.90
D3	HB3	1H	2.43
D3	HN	1H	8.66
Y4	HA	1H	3.78
Y4	HB2	1H	2.63
Y4	HD1	1H	6.80
Y4	HE1	1H	6.53
Y4	HH	1H	9.18
Y4	HNα	1H	5.63
W5	HA	1H	4.47
W5	HB2	1H	3.10
W5	HB3	1H	2.90
W5	HD1	1H	7.06
W5	HE1	1H	10.79
W5	HE3	1H	7.59
W5	HH2	1H	7.05
W5	HN	1H	8.00
W5	HZ2	1H	7.32
W5	HZ3	1H	6.98
E6	HA	1H	4.20
E6	HB2	1H	1.90
E6	HB3	1H	1.74
E6	HG2	1H	2.23
E6	HN	1H	8.03
G7	HA	1H	3.69
G7	HN	1H	7.90
L8	HA	1H	4.21
L8	HB3	1H	1.46
L8	HD1	1H	0.87
L8	HD2	1H	0.83
L8	HG	1H	1.57
L8	HN	1H	7.83
F1	C	13C	168.0
F1	CA	13C	53.1
F1	CB	13C	36.7
F1	CD1	13C	129.3
F1	CE1	13C	128.5
F1	CZ	13C	127.0
M2	C	13C	170.9
M2	CA	13C	51.5
M2	CB	13C	31.8
M2	CG	13C	29.0
D3	C	13C	174.1
D3	CA	13C	46.4
D3	CB	13C	33.2
D3	CO	13C	173.3
Y4	C	13C	170.8
Y4	CA	13C	62.4
Y4	CB	13C	36.4
Y4	CD1	13C	129.9
Y4	CE1	13C	114.6
Y4	CZ	13C	155.8
W5	C	13C	171.5
W5	CA	13C	53.2
W5	CB	13C	27.2
W5	CD1	13C	123.4
W5	CE3	13C	118.3
W5	CH2	13C	120.8
W5	CZ2	13C	111.2
W5	CZ3	13C	118.1
E6	C	13C	171.4
E6	CA	13C	52.0
E6	CB	13C	27.0
E6	CG	13C	29.8
E6	CO	13C	174.1

G7	C	13C	168.5
G7	CA	13C	41.8
L8	C	13C	174.3
L8	CA	13C	50.8
L8	CB	13C	40.6
L8	CD1	13C	22.9
L8	CD2	13C	21.4
L8	CG	13C	24.1
Nt	N	15N	106.3
M2	N	15N	120.9
D3	N	15N	115.3
W5	N	15N	120.7
W5	NE1	15N	132.5
E6	N	15N	119.8
G7	N	15N	107.4
L8	N	15N	119.7

minor

F1	HB2	1H	3.06
F1	HB3	1H	2.91
F1	HN	1H	8.08
M2	HA	1H	4.47
M2	HB2	1H	1.76
M2	HB3	1H	1.89
M2	HG	1H	2.41
M2	HN	1H	8.69
Nt	HN1	1H	7.38
Nt	HN2	1H	7.02
D3	HA	1H	4.48
D3	HB2	1H	2.62
D3	HB3	1H	2.48
D3	HN	1H	8.44
Y4	HA	1H	3.42
Y4	HB2	1H	2.55
Y4	HB3	1H	2.49
Y4	HD1	1H	6.78
Y4	HNβ	1H	9.47
W5	HA	1H	4.52
W5	HB2	1H	3.10
W5	HB3	1H	2.97
W5	HD1	1H	7.11
W5	HE1	1H	10.78
W5	HE3	1H	7.60
W5	HN	1H	7.96
E6	HA	1H	4.30
E6	HB2	1H	1.91
E6	HB3	1H	1.78
E6	HG2	1H	2.25
E6	HN	1H	8.29
G7	HA	1H	3.74
G7	HN	1H	7.91
L8	HA	1H	4.21
L8	HN	1H	7.81
F1	C	13C	167.8
M2	C	13C	170.2
M2	CG	13C	33.2
D3	C	13C	169.4
Y4	CD1	13C	129.9
W5	C	13C	172.1
W5	CD1	13C	123.5
E6	C	13C	171.5
E6	CG	13C	33.3
G7	C	13C	168.5
L8	C	13C	174.3
M2	N	15N	122.4
Nt	N	15N	106.6
D3	N	15N	119.8
Y4	N	15N	142.9
W5	N	15N	119.2
E6	N	15N	120.3
G7	N	15N	107.6

unassigned, 2nd and 3rd minor

M2_2	HB3	1H	1.92
M2_2	HN	1H	8.83
D3_2	HA	1H	4.48
D3_2	HB2	1H	2.78
D3_2	HB3	1H	2.38
D3_2	HN	1H	8.56
D3_3	HN	1H	8.75
Nt_2	HN1	1H	7.46
Nt_2	HN2	1H	7.06
Y4_3	HA	1H	3.87
Y4_3	HB2	1H	2.67

Y4_3	HD1	1H	6.82	L8_2	HA	1H	4.21
W5_2	HA	1H	4.51	L8_2	HN	1H	7.87
W5_2	HB2	1H	3.11	L8_3	HA	1H	4.19
W5_2	HB3	1H	2.90	L8_3	HN	1H	7.75
W5_2	HD1	1H	7.13	Y4_2	C	13C	169.6
W5_2	HN	1H	8.05	W5_2	C	13C	172.2
W5_3	HA	1H	4.53	W5_2	CD1	13C	123.4
W5_3	HB2	1H	3.02	W5_3	CD1	13C	123.4
W5_3	HB3	1H	2.87	E6_2	C	13C	171.5
W5_3	HD1	1H	7.00	G7_2	C	13C	168.5
W5_3	HE1	1H	10.72	M2_2	N	15N	120.9
W5_3	HE3	1H	7.52	D3_3	N	15N	116.0
W5_3	HN	1H	8.16	W5_2	N	15N	125.6
E6_2	HA	1H	4.21	W5_3	N	15N	123.3
E6_2	HB2	1H	1.92	E6_2	N	15N	119.3
E6_2	HB3	1H	1.77	E6_3	N	15N	120.4
E6_2	HG2	1H	2.24	G7_2	N	15N	107.3
E6_2	HN	1H	8.18	L8_2	N	15N	120.0
E6_3	HA	1H	4.21	?	?	1H	3.77
E6_3	HB2	1H	1.89	?	?	1H	2.63
E6_3	HB3	1H	1.74	?	?	1H	3.89
E6_3	HG2	1H	2.23	?	?	1H	6.73
E6_3	HN	1H	8.14	?	?	1H	3.69
G7_2	HA	1H	3.70	?	?	1H	7.89
G7_2	HN	1H	7.99	?	?	1H	8.42

* The chemical shifts were extracted from the NMR spectra recorded at a peptide concentration of 2 mM in DMSO-d_6 and 25 °C using an 800 MHz spectrometer.

Fluorescence spectroscopy

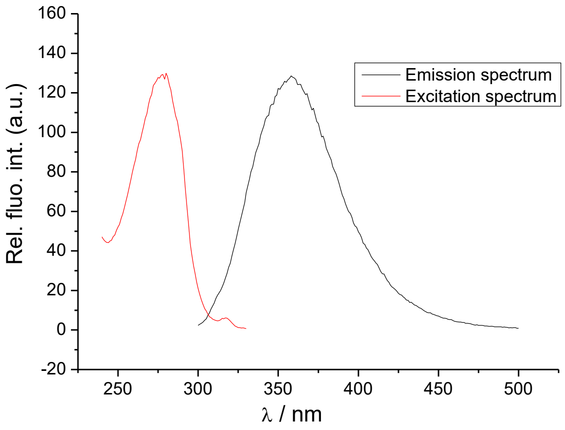

No	c (M)	Abs(max)
1	5×10^{-6}	127.3
2	1×10^{-5}	249.8
3	1.5×10^{-5}	358.6

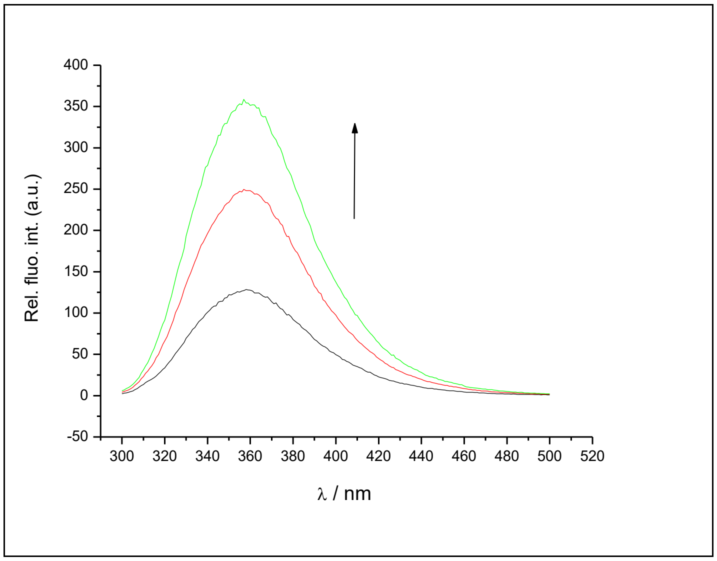

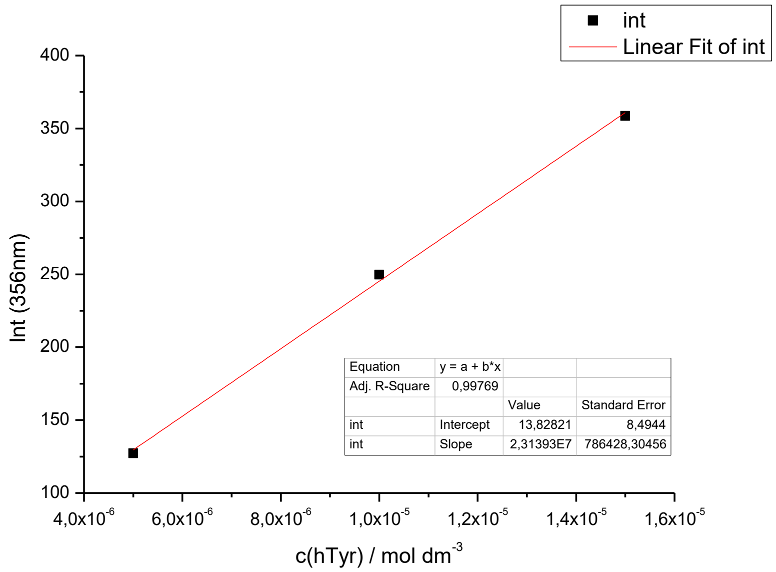

Figure S14. Fluorescence spectra of α-hydrazino peptide **h(4)**, λ_{exc} = 280 nm, (up); linear dependence (—) of the fluorescence intensity λ_{exc} = 280nm, λ_{em} = 356 nm (■) on the α-hydrazino peptide **h(4)** concentration (middle and down), (Na-cacodylate buffer, I=0.05 M, pH = 7.0, 20 °C).

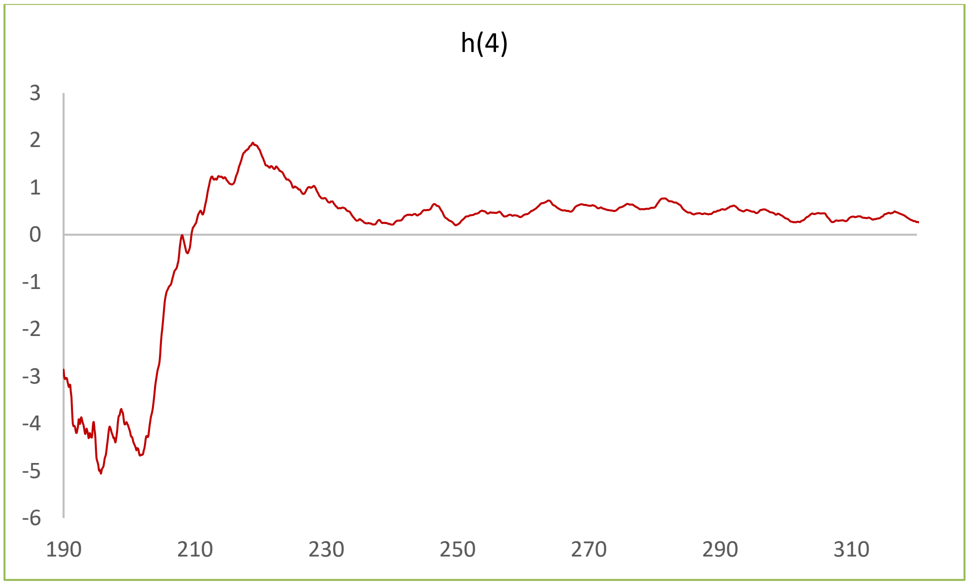

Figure S15. CD spectrum of α-hydrazino peptide (**h4**) (1×10-5 M solution in water at room temperature).

α-hydrazino peptide h(8):

Yield: 40 % (25 mg). t_R = 11.53 min. HRMS: calculated for $C_{51}H_{67}N_{11}O_{13}S$ $[M+H]^+$ 1074,4719; found 1074,4728.

Table S7. Amino acids, reagents and applied programs for synthesis on a solid support,– introduction of the first amino acid, 2. – introduction of others and 3. – removal of Fmoc group 4. – repeated introduction of the same amino acid

Program	Sample no.	Amino acids and reagents	Mass (mg)
1	1	**Fmoc-hLeu-OH**<br>HATU<br>HOBt	63.61<br>68.44<br>24.32
4	2	**Fmoc-hLeu-OH**<br>HATU<br>HOBt	63.61<br>68.44<br>24.32
2	3	Fmoc-Gly-OH<br>HATU<br>HOBt	53.52<br>68.44<br>24.32
2	4	Fmoc-Glu(OtBu)-OH<br>HBTU<br>HOBt	76.59<br>68.27<br>24.32
2	5	Fmoc-Trp(Boc)-OH<br>HBTU<br>HOBt	94.78<br>68.27<br>24.32
2	6	Fmoc-Tyr(tBu)-OH<br>HBTU<br>HOBt	82.72<br>68.27<br>24.32
2	7	Fmoc-Asp(OtBu)-OH<br>HBTU<br>HOBt	74.06<br>68.27<br>24.32
2	8	Fmoc-Met-OH<br>HBTU<br>HOBt	66.86<br>68.27<br>24.32
2	9	Fmoc-Phe-OH<br>HBTU<br>HOBt	69.73<br>68.27<br>24.32
3	10	-	-

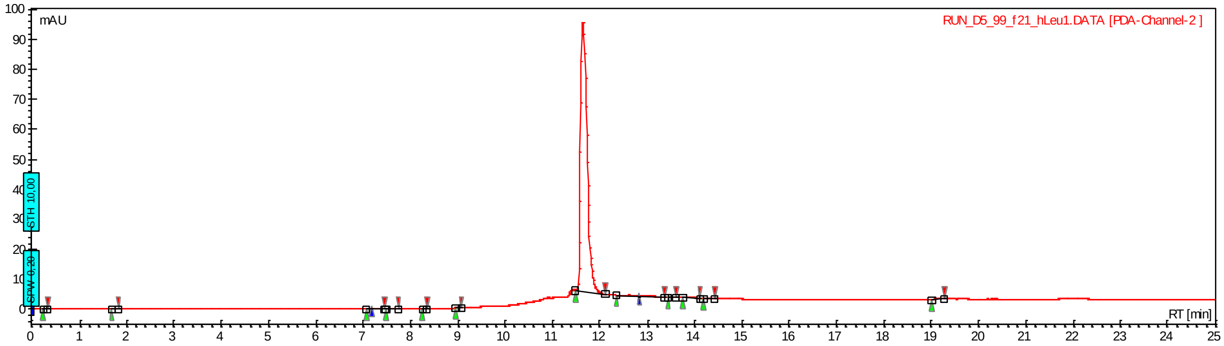

Figure S16. HPLC-DAD chromatogram of purified α-hydrazino peptide **h(8)**. HPLC conditions are given in General procedure.

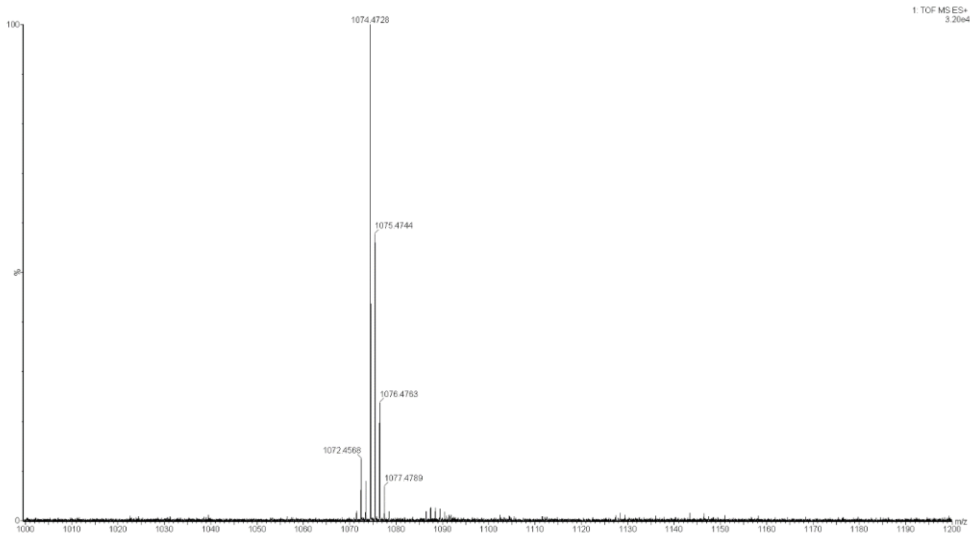

Figure S17. HRMS spectrum of α-hydrazino peptide **h(8)**.

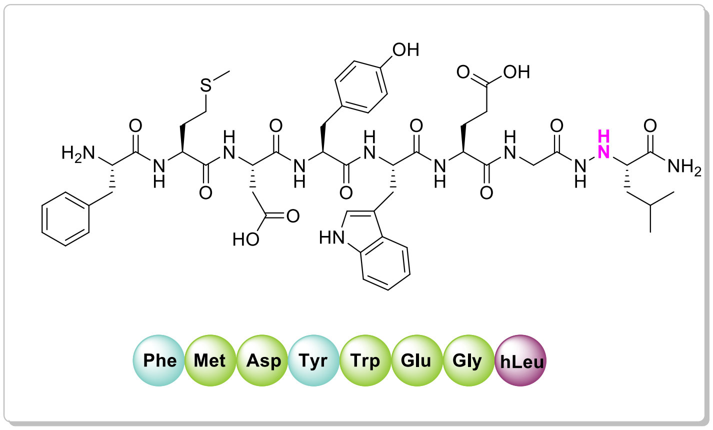

Figure S18. Chemical structure of α-hydrazino peptide **h(8)**.

Table S8. Chemical shift data of α-hydrazino peptide (h8)

Group	Atom	Nucleus	Chemical shift
	**major Z isomer**		
Nt	HN1	1H	7.37
Nt	HN2	1H	7.01
F1	HA	1H	4.05
F1	HB2	1H	3.06
F1	HB3	1H	2.89
F1	HD	1H	7.22
F1	HE	1H	7.32
F1	HN	1H	8.05
F1	HZ	1H	7.26
M2	HA	1H	4.42
M2	HB2	1H	1.89
M2	HB3	1H	1.73
M2	HE	1H	2.00
M2	HG	1H	2.42
M2	HG2	1H	2.44
M2	HG3	1H	2.40
M2	HN	1H	8.67
D3	HA	1H	4.54
D3	HB2	1H	2.66
D3	HB3	1H	2.47
D3	HN	1H	8.33
Y4	HA	1H	4.37
Y4	HB2	1H	2.83
Y4	HB3	1H	2.64
Y4	HD	1H	6.94
Y4	HE	1H	6.58
Y4	HH	1H	9.16
Y4	HN	1H	7.82
W5	HA	1H	4.54
W5	HB2	1H	3.14
W5	HB3	1H	2.95
W5	HD1	1H	7.13
W5	HE1	1H	10.76
W5	HE3	1H	7.57
W5	HH2	1H	7.03
W5	HN	1H	8.11
W5	HZ2	1H	7.30
W5	HZ3	1H	6.96
E6	HA	1H	4.26
E6	HB2	1H	1.90
E6	HB3	1H	1.75
E6	HG	1H	2.23
E6	HN	1H	8.06
G7	HA2	1H	3.58
G7	HA3	1H	3.66
G7	HN	1H	7.91
L8	HA	1H	3.23
L8	HB	1H	1.33
L8	HD	1H	0.86
L8	HD1	1H	0.87
L8	HD2	1H	0.86
L8	HG	1H	1.73
L8	HNβ	1H	9.23
L8	HNα	1H	4.93
F1	C	13C	167.8
F1	CA	13C	53.1
F1	CB	13C	36.8
M2	C	13C	170.3
M2	CA	13C	51.5
M2	CB	13C	32.3
M2	CE	13C	14.4
M2	CG	13C	29.0
D3	C	13C	170.3
D3	CA	13C	49.4
D3	CB	13C	35.9
D3	CO	13C	199.7
Y4	C	13C	170.9
Y4	CA	13C	54.0
Y4	CB	13C	36.3
Y4	CZ	13C	155.7
W5	C	13C	171.4
W5	CA	13C	53.2
W5	CB	13C	27.3
E6	C	13C	171.2
E6	CA	13C	51.9
E6	CB	13C	27.3
E6	CG	13C	29.7
E6	CO	13C	174.1
G7	C	13C	167.9

G7	CA	13C	40.2	M2	N	15N	121.7
L8	C	13C	175.4	D3	N	15N	120.0
L8	CA	13C	62.3	Y4	N	15N	117.6
L8	CB	13C	40.0	W5	N	15N	119.9
L8	CD1	13C	22.6	E6	N	15N	119.7
L8	CD2	13C	22.3	G7	N	15N	106.3
L8	CG	13C	24.2	L8	Nβ	15N	141.7
Nt	N	15N	105.7				

minor Z isomer

Nt	HN1	1H	7.38	L8	HA	1H	3.22
G7	HA	1H	3.60	L8	HNβ	1H	9.22
G7	HN	1H	7.88	E6	C	13C	171.3
				G7	N	15N	106.2

major E isomer

F1	HA	1H	4.07	E6	HN	1H	8.08
M2	HA	1H	4.47	G7	HA	1H	4.00
M2	HN	1H	8.53	G7	HN	1H	7.70
Nt	HN1	1H	7.44	L8	HA	1H	3.26
Nt	HN2	1H	7.04	L8	HNβ	1H	8.20
Y4	HN	1H	7.80	E6	CA	13C	51.7
W5	HD1	1H	7.13	G7	C	13C	171.8
W5	HE1	1H	10.75	G7	CA	13C	40.0
W5	HZ2	1H	7.31	M2	N	15N	121.1
E6	HA	1H	4.31	Nt	N	15N	109.0
E6	HB2	1H	1.92	Y4	N	15N	117.5
E6	HG	1H	2.26	G7	N	15N	106.1
				L8	Nβ	15N	140.9

minor E isomer and unassigned

G7	HA2	1H	4.08	?	?	1H	7.93
G7	HA3	1H	3.90	?	?	1H	7.84
G7	HN	1H	7.67	?	?	15N	107.3
?	?	1H	7.99	?	?	15N	105.5

* The chemical shifts were extracted from the NMR spectra recorded at a peptide concentration of 2 mM in DMSO-d_6 and 25 °C using an 800 MHz spectrometer.

Fluorescence spectroscopy:

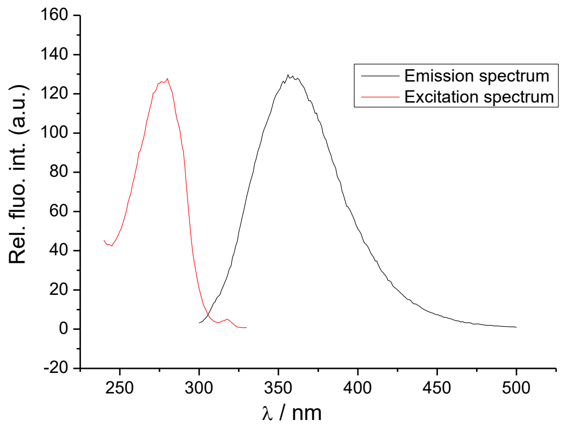

No	c (M)	Abs(max)
1	5×10^{-6}	128.0
2	1×10^{-5}	253.9
3	1.5×10^{-5}	370.5

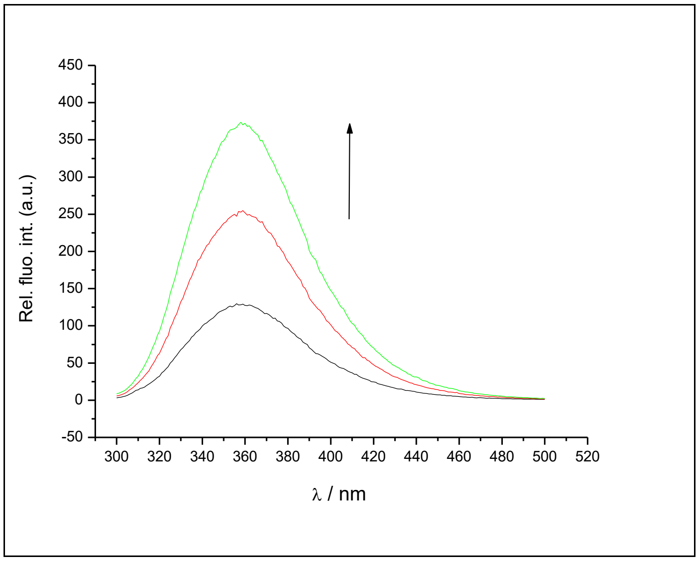

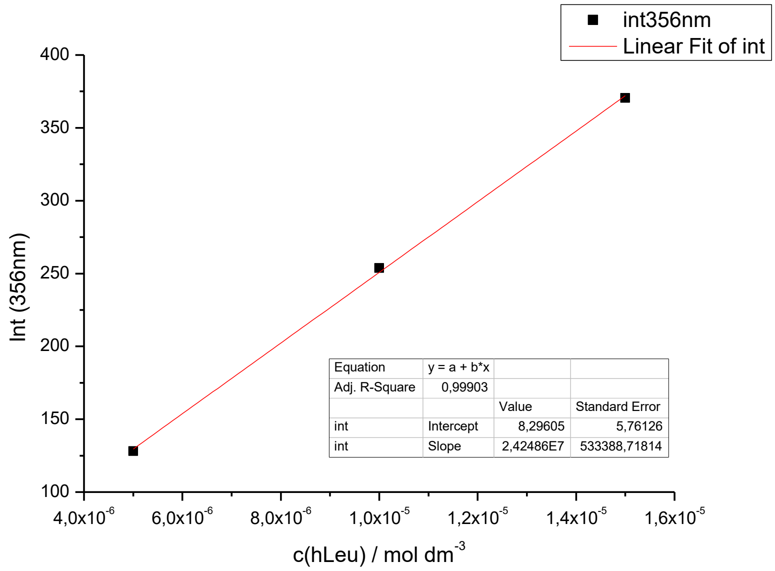

Figure S19. Fluorescence spectra of α-hydrazino peptide **h(8)**, λ_{exc} = 280 nm, (up); linear dependence (—) of the fluorescence intensity λ_{exc} = 280nm, λ_{em} = 356 nm (■) on the α-hydrazino peptide **h(8)** concentration (middle and down), (Na-cacodylate buffer, I=0.05 M, pH = 7.0, 20 °C).

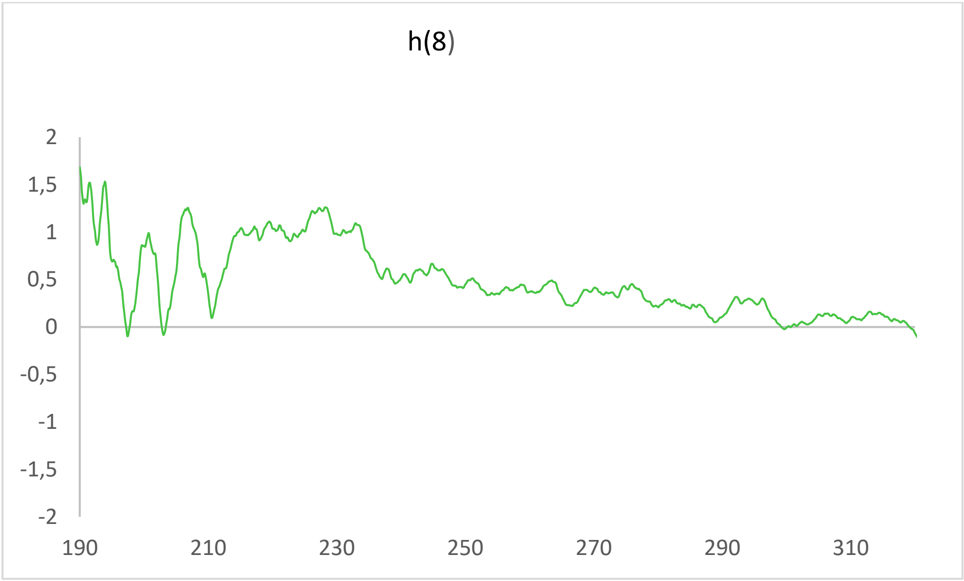

Figure S20. CD spectrum of α-hydrazino peptide **h(8)** (1×10-5 M solution in water at room temperature).

α-hydrazino peptide h(1,4,8)

Yield: 35 % (23 mg). t_R = 11.31 min. HRMS: calculated for $C_{51}H_{69}N_{13}O_{13}S$ $[M+2H]^{2+}$ 552.7508; found 552.7505.

Table S9. Amino acids, reagents and applied programs for synthesis on a solid support, 1. – introduction of the first amino acid, 2. – introduction of others, 3. – removal of Fmoc group and 4. – repeated introduction of the same amino acid

Program	Sample no.	Amino acids and reagents	Mass (mg)
1	1	**Fmoc-hLeu-OH**<br>HATU<br>HOBt	63.61<br>68.44<br>24.32
4	2	**Fmoc-hLeu-OH**<br>HATU<br>HOBt	63.61<br>68.44<br>24.32
2	3	Fmoc-Gly-OH<br>HATU<br>HOBt	53.52<br>68.44<br>24.32
2	4	Fmoc-Glu(OtBu)-OH<br>HBTU<br>HOBt	76.59<br>68.27<br>24.32
2	5	Fmoc-Trp(Boc)-OH<br>HBTU<br>HOBt	94.78<br>68.27<br>24.32
2	6	**Fmoc-hTyr(tBu)-OH**<br>HATU<br>HOBt	82.72<br>68.44<br>24.32
4	7	**Fmoc-hTyr(tBu)-OH**<br>HATU<br>HOBt	82.72<br>68.44<br>24.32
2	8	Fmoc-Asp(OtBu)-OH<br>HATU<br>HOBt	74.06<br>68.44<br>24.32
2	9	Fmoc-Met-OH<br>HBTU<br>HOBt	66.86<br>68.27<br>24.32
2	10	**Fmoc-hPhe-OH**<br>HBTU<br>HOBt	72.46<br>68.27<br>24.32
2	11	**Fmoc-hPhe-OH**<br>HBTU<br>HOBt	72.46<br>68.27<br>24.32
3	12	-	-

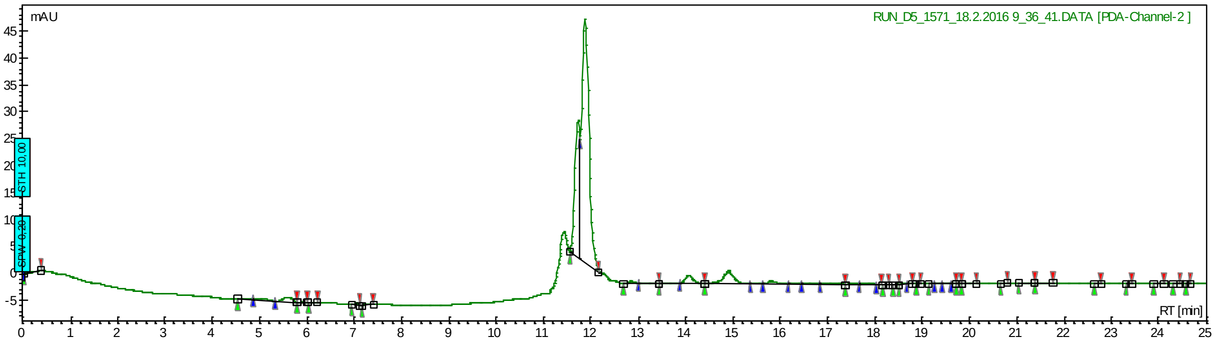

Figure S21. HPLC-DAD chromatogram of purified α-hydrazino peptide **h(1,4,8).** HPLC conditions are given in General procedure.

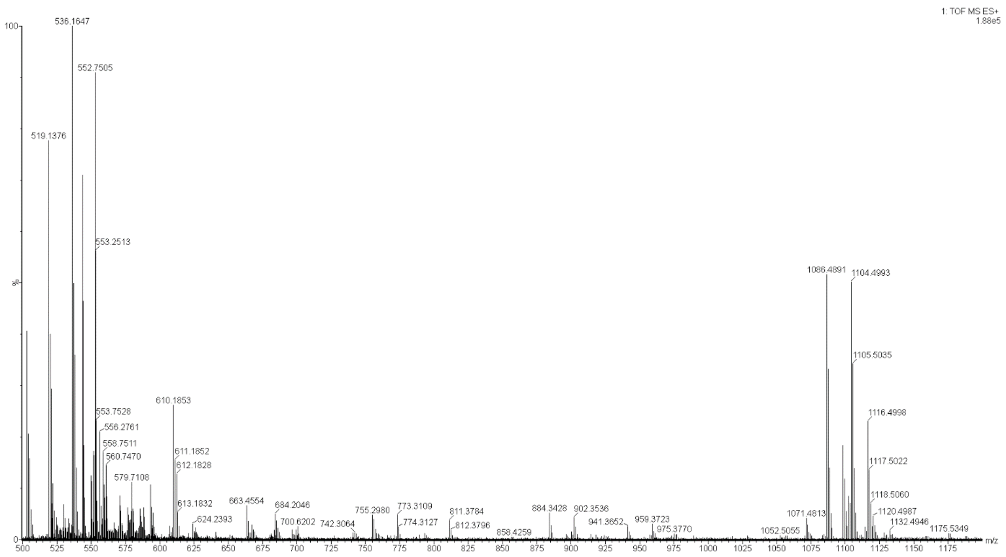

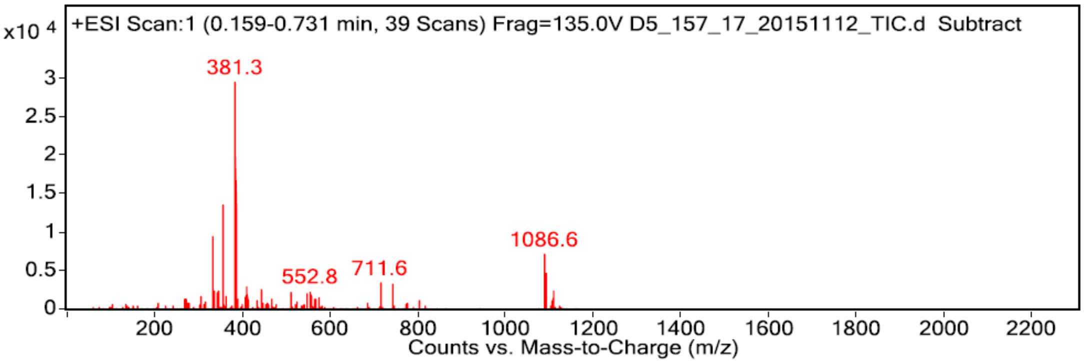

Figure S22. HRMS spectrum of α-hydrazino peptide **h(1,4,8)**.

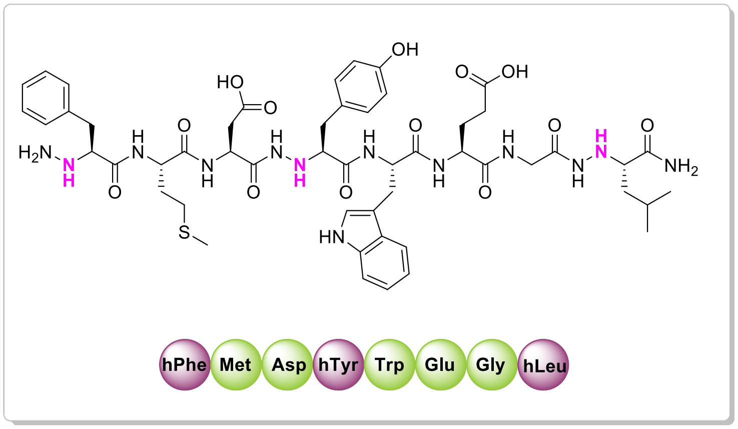

Figure S23. Chemical structure of α-hydrazino peptide **h(1,4,8)**.

Table S10. Chemical shift data of α-hydrazino peptide (h(1,4,8))

Group	Atom	Nucleus	Chemical shift
	major		
F1	HA	1H	3.80
F1	HB2	1H	3.02
F1	HB3	1H	2.72
F1	HD1	1H	7.25
F1	HE1	1H	7.28
F1	HNβ	1H	8.87
F1	HNα	1H	5.59
F1	HZ	1H	7.21
Nt	HN1	1H	7.37
Nt	HN2	1H	7.03
M2	HA	1H	4.39
M2	HB2	1H	1.93
M2	HB3	1H	1.84
M2	HE	1H	2.04
M2	HG	1H	2.46
M2	HN	1H	8.60
D3	HA	1H	4.37
D3	HB2	1H	2.91
D3	HB3	1H	2.44
D3	HN	1H	8.63
Y4	HA	1H	3.80
Y4	HB2	1H	2.64
Y4	HD1	1H	6.83
Y4	HE1	1H	6.54
Y4	HNβ	1H	9.13
Y4	HNα	1H	5.67
W5	HA	1H	4.48
W5	HB2	1H	3.10
W5	HB3	1H	2.90
W5	HD1	1H	7.09
W5	HE1	1H	10.81
W5	HE3	1H	7.61
W5	HH2	1H	7.07
W5	HN	1H	7.98
W5	HZ2	1H	7.33
W5	HZ3	1H	6.98
E6	HA	1H	4.24
E6	HB2	1H	1.90
E6	HB3	1H	1.73
E6	HG	1H	2.22
E6	HN	1H	8.00
G7	HA2	1H	3.65
G7	HA3	1H	3.57
G7	HN	1H	7.92
L8	HA	1H	3.24
L8	HB2	1H	1.36
L8	HD1	1H	0.88
L8	HG	1H	1.74
L8	HNβ	1H	9.26

L8	HNα	1H	4.96
F1	CA	13C	61.8
F1	CD1	13C	129.2
F1	CE1	13C	127.8
F1	CG	13C	137.2
F1	CZ	13C	126.2
M2	CA	13C	51.5
M2	CB	13C	31.6
M2	CE	13C	14.4
M2	CG	13C	29.1
D3	CA	13C	46.4
Y4	CA	13C	62.0
Y4	CB	13C	35.9
Y4	CD1	13C	129.6
Y4	CE1	13C	114.6
Y4	CG	13C	126.0
Y4	CZ	13C	155.8
W5	CA	13C	53.1
W5	CB	13C	27.3
W5	CD1	13C	123.3
W5	CD2	13C	126.3
W5	CE2	13C	135.2
W5	CE3	13C	118.3
W5	CG	13C	109.0
W5	CH2	13C	120.4
W5	CZ2	13C	110.9
W5	CZ3	13C	118.0
E6	CA	13C	51.7
E6	CB	13C	27.1
E6	CG	13C	29.7
G7	C	13C	174.6
G7	CA	13C	40.5
L8	C	13C	167.0
L8	CA	13C	62.0
L8	CB	13C	39.6
L8	CD1	13C	22.7
L8	CG	13C	24.0
D3	N	15N	112.3
W5	N	15N	118.0
W5	NE1	15N	130.0
E6	N	15N	116.8
G7	N	15N	103.5

minor

F1	HA	1H	3.81
M2	HA	1H	4.47
M2	HB2	1H	1.89
M2	HB3	1H	1.79
M2	HE	1H	1.99
M2	HG	1H	2.43
M2	HN	1H	8.59
Nt	HN1	1H	7.40
Nt	HN2	1H	7.04
D3	HA	1H	4.49
D3	HB2	1H	2.63
D3	HB3	1H	2.50
D3	HN	1H	8.39
Y4	HA	1H	3.42
Y4	HB2	1H	2.59
Y4	HB3	1H	2.52
Y4	HD1	1H	6.81
Y4	HE1	1H	6.55
Y4	HN	1H	9.47
W5	HA	1H	4.53
W5	HB2	1H	3.10
W5	HB3	1H	2.97
W5	HD1	1H	7.12
W5	HE1	1H	10.79
W5	HZ2	1H	7.33
E6	HA	1H	4.34
E6	HB2	1H	1.91
E6	HB3	1H	1.76
E6	HG	1H	2.25
E6	HN	1H	8.25
G7	HA	1H	3.61
G7	HN	1H	7.88
L8	HA	1H	3.26
L8	HN	1H	9.29
M2	CA	13C	45.1
M2	CB	13C	32.0
M2	CE	13C	14.4
M2	CG	13C	29.2
D3	CA	13C	48.0
Y4	CA	13C	65.0
W5	CA	13C	52.8
E6	CA	13C	51.9
E6	CB	13C	27.2

G7	CA	13C	40.3	E6	N	15N	117.5
D3	N	15N	116.7	G7	N	15N	103.3

**2nd minor**							
F1_2	HB2	1H	3.00	G7_2	HN	1H	7.67
F1_2	HB3	1H	2.73	Nt_2	HN1	1H	7.43
F1_2	HD1	1H	7.19	Nt_2	HN2	1H	7.06
F1_2	HE1	1H	7.28	W5_2	HE1	1H	10.74
F1_2	HZ	1H	7.15	G7_2	N	15N	112.2
Y4_2	HA	1H	3.89				
Y4_2	HB3	1H	2.67	**other form of 2nd minor isomer**			
F1_2	CD1	13C	127.8	G7_2	HA2	1H	4.08
F1_2	CZ	13C	126.1	G7_2	HA3	1H	3.92
G7_2	HA	1H	4.00	G7_2	HN	1H	7.64

**3rd minor**							
M2_3	HA	1H	4.45	W5_3	HD1	1H	7.12
M2_3	HB2	1H	1.98	W5_3	HE1	1H	10.79
M2_3	HN	1H	8.70	W5_3	HN	1H	7.96
D3_3	HA	1H	4.38	E6_3	HA	1H	4.30
D3_3	HB2	1H	2.92	E6_3	HB2	1H	1.92
D3_3	HB3	1H	2.47	E6_3	HB3	1H	1.73
D3_3	HN	1H	8.73	E6_3	HG	1H	2.26
Y4_3	HD1	1H	6.63	E6_3	HN	1H	8.07
Y4_3	HE1	1H	6.42	G7_3	HA	1H	4.03
W5_3	HA	1H	4.50	G7_3	HN	1H	7.69
W5_3	HB2	1H	3.10	Y4_3	CD1	13C	130.0
W5_3	HB3	1H	2.89	Y4_3	CE1	13C	114.5
				W5_3	CD1	13C	123.5

**4th minor**				G7_4	HN	1H	7.94
W5_4	HA	1H	4.55				
W5_4	HB2	1H	3.02				
W5_4	HB3	1H	2.86	**other form of 4th minor isomer**			
W5_4	HN	1H	8.17	G7_4	HA2	1H	3.71
E6_4	HA	1H	4.24	G7_4	HA3	1H	3.60
E6_4	HB2	1H	1.88	G7_4	HN	1H	7.95
E6_4	HB3	1H	1.73				
E6_4	HG	1H	2.22				
E6_4	HN	1H	8.11				
G7_4	HA	1H	3.65				

* The chemical shifts were extracted from the NMR spectra recorded at a peptide concentration of 2 mM in DMSO-d_6 and 25 °C using an 800 MHz spectrometer.

Fluorescence spectroscopy

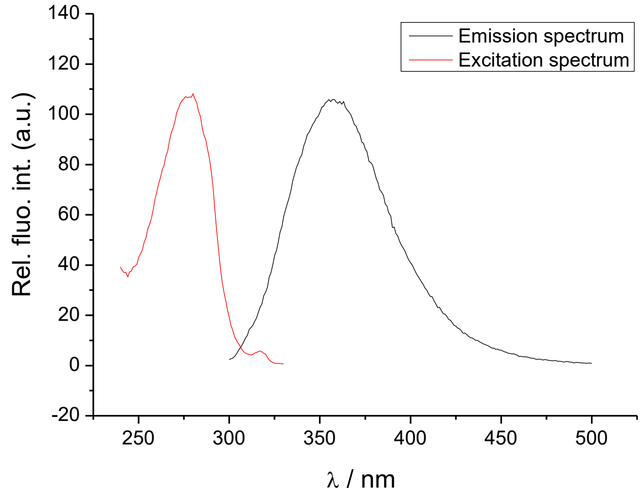

No	c (M)	Abs (max)
1	5×10^{-6}	105.0
2	1×10^{-5}	194.6
3	1.5×10^{-5}	280.7

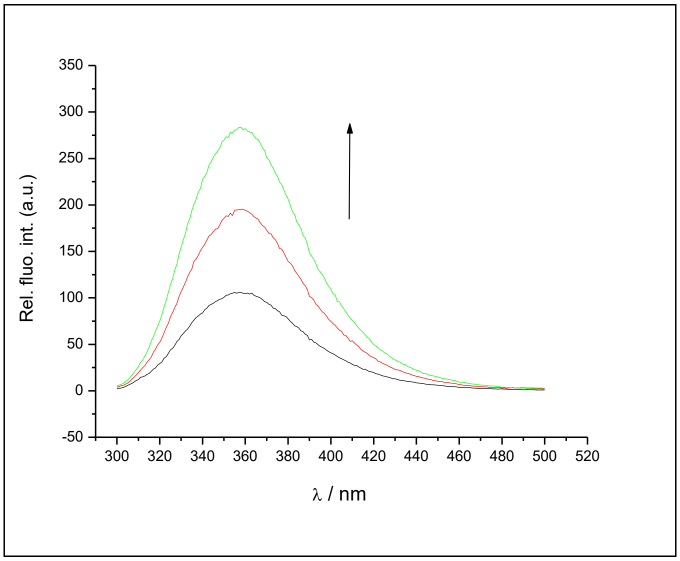

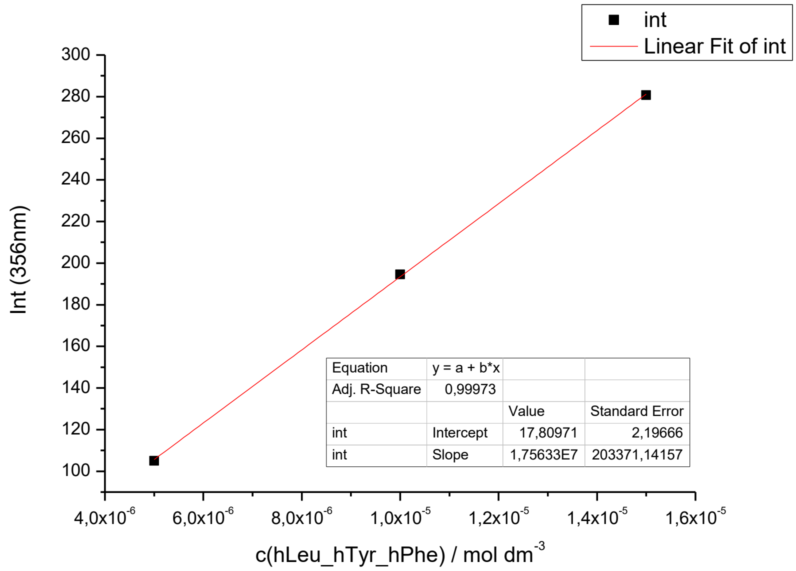

Figure S24. Fluorescence spectra of α-hydrazino peptide **h(1,4,8)**, λ_{exc} = 280 nm, (up); linear dependence (—) of the fluorescence intensity λ_{exc} = 280 nm, λ_{em} = 356 nm (■) on the α-hydrazino peptide **h(1,4,8)** concentration (down), (Na-cacodylate buffer, I=0.05 M, pH = 7.0, 20 °C).

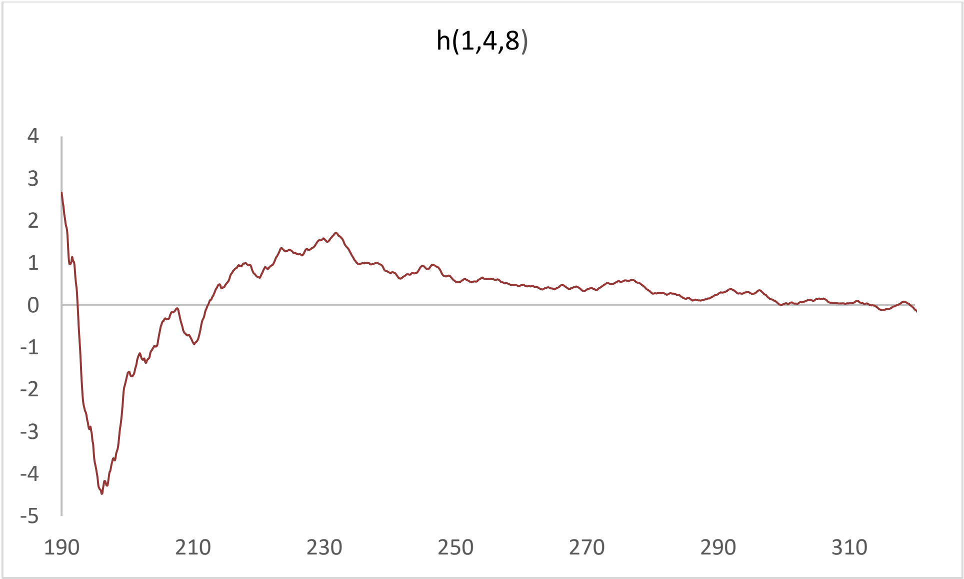

Figure S25. CD spectrum of α-hydrazino peptide **h(1,4,8)** (1×10-5 M solution in water at room temperature).

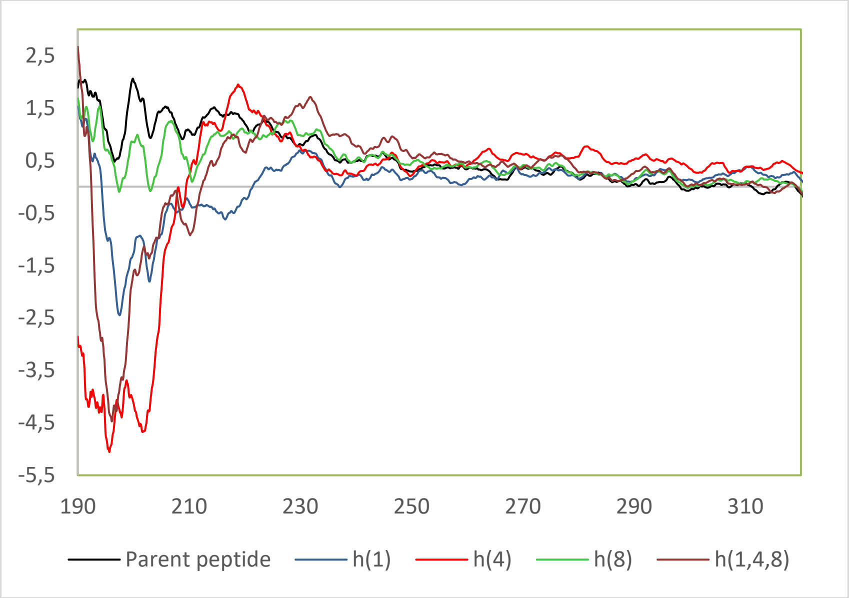

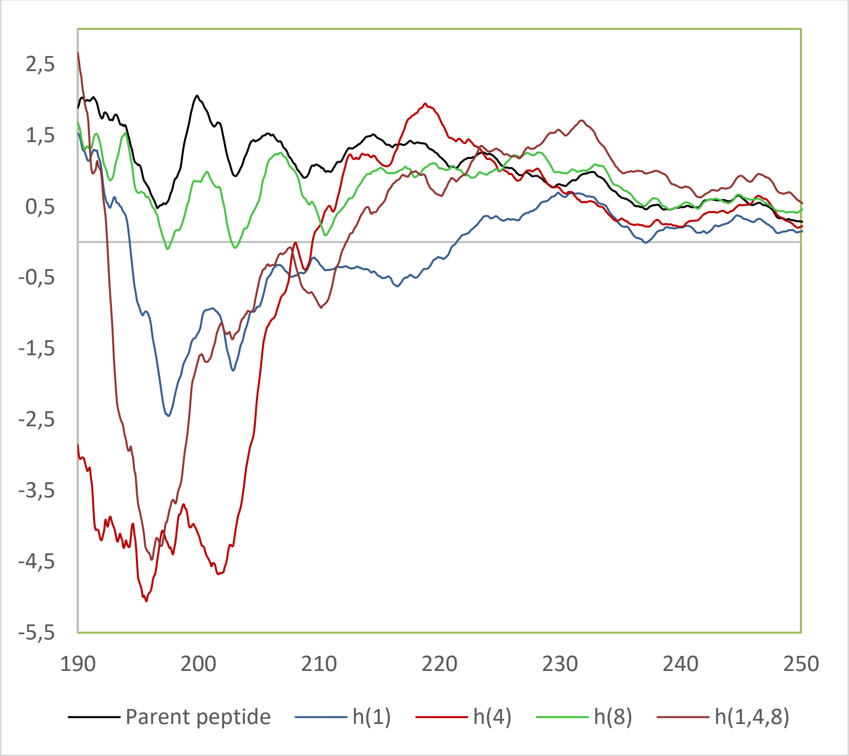

Figure S26. Superimposed CD spectra of parent peptides and α-hydrazino peptides **(h1)**, **(h4)**, **(h8)** and **h(1,4,8)**.

Table S11: NMR restraints and structural statistics for the 10 lowest energy peptide structures, calculated with AMBER.

	parent	α-hydrazino peptide **h(1)**		α-hydrazino peptide **h(4)**		α-hydrazino peptide **h(8)**	
		left	right	E isomer	Z isomer	γ-turn structure	β-turn structure
NOE-derived distance restraints	213	77	42	160	79	179	
Total	131	77	42	71	44	92	
intra-residue & sequential (I*i*-*j*I ≤ 1)	111	67	42	64	43	88	
medium-range (I*i*-*j*I ≤ 5)	20	10	0	4	1	4	
long-range (I*i*-*j*I ≤ 5)	0	0	0	0	0	0	
NOE constraints per restrained residue	16.4	9.6	5.3	89	5.5	11.5	
Dihedral-angle restraints	0	0	0	0	0	0	
Structural statistics							
NOE violations >0.3 Å	0	0	0	0	0	0	0
Number of structures in ensemble	10	10	10	10	10	10	10
Pairwise backbone or heavy atom r.m.s.d. (Å)							
backbone	1.30 ± 0.52	1.83 ± 0.76	2.11 ± 0.57	1.60 ± 0.53	0.83 ± 0.33	3.00 ± 0.75	2.84 ± 0.70
M2-G7 backbone	0.66 ± 0.30	0.75 ± 0.28	1.44 ± 0.54	0.88 ± 0.34	0.68 ± 0.34	2.27 ± 0.63	2.18 ± 0.56
heavy atom	2.33 ± 0.62	3.04 ± 1.00	3.95 ± 0.79	2.71 ± 0.74	2.21 ± 0.40	5.12 ± 0.95	5.02 ± 0.74
M2-G7 heavy atom	1.61 ± 0.33	1.53 ± 0.31	2.99 ± 0.88	1.96 ± 0.52	1.89 ± 0.42	4.53 ± 0.83	4.45 ± 0.81

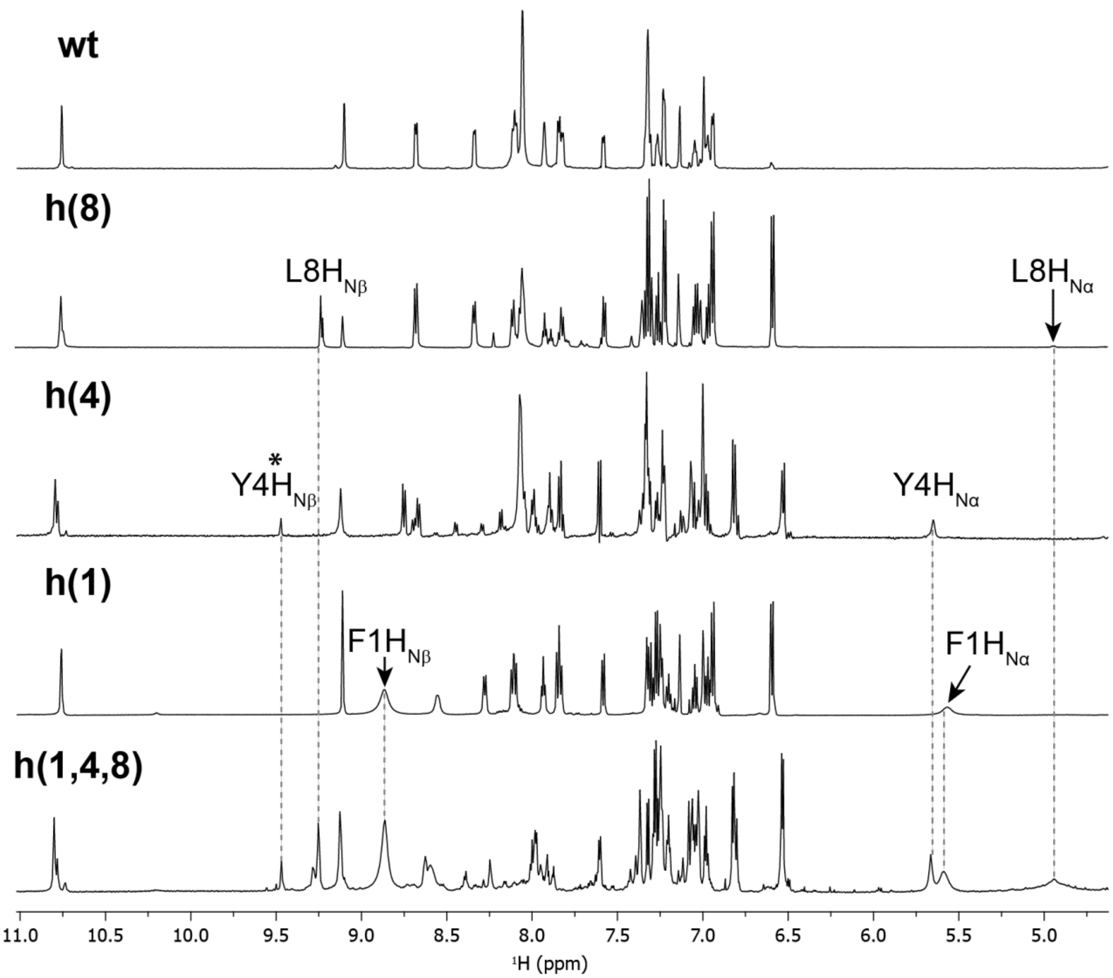

Figure S27: Comparison of 1D NMR spectra of all α-hydrazino peptides with respect to the parent peptide. In the 1D NMR spectrum of α-hydrazino peptide **h(4)** Y4H$_{N\beta}$* represents the signal for the minor form since we could not determine corresponding peak for the major form. The NMR spectra were acquired at 2 mM peptide concentration and 25 °C using a 600 and an 800 MHz NMR spectrometers (for the parent, wt, and α-hydrazino peptide **h(1,4,8)**) spectrometers.

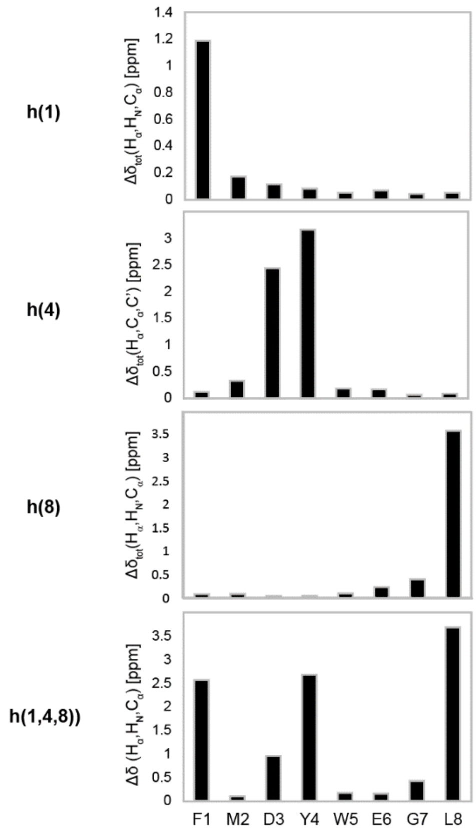

Figure S28: Chemical shift perturbation analysis for major forms of α-hydrazino peptidomimetics. The values were calculated from chemical shift values of H_α, C_α and H_N (or C’ in the case of α-hydrazino peptide **h(4)**) using equation 1.

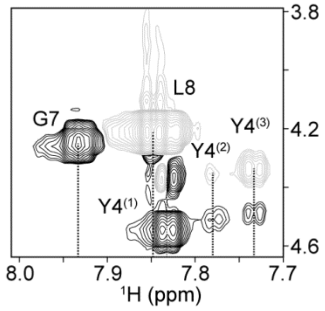

Figure S29: 2D ^{1}H-^{1}H ROESY spectrum of α-hydrazino peptide **h(1)**, which was recorded at 200 ms mixing time at 2 mM peptide concentration and 25 °C using a 600 MHz spectrometer. Antiphase cross-peaks (grey) indicate the presence of chemical exchange between different conformational forms in solution.

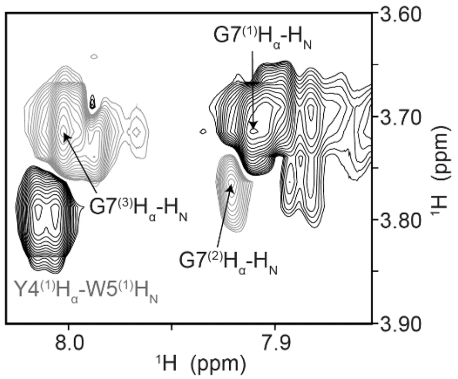

Figure S30: 2D ^{1}H-^{1}H ROESY spectrum of α-hydrazino peptide **h(4)**, which was recorded at 200 ms mixing time at 2 mM peptide concentration and 25 °C using a 600 MHz spectrometer. Antiphase cross-peaks (grey) indicate the presence of chemical exchange between different conformational forms in solution.

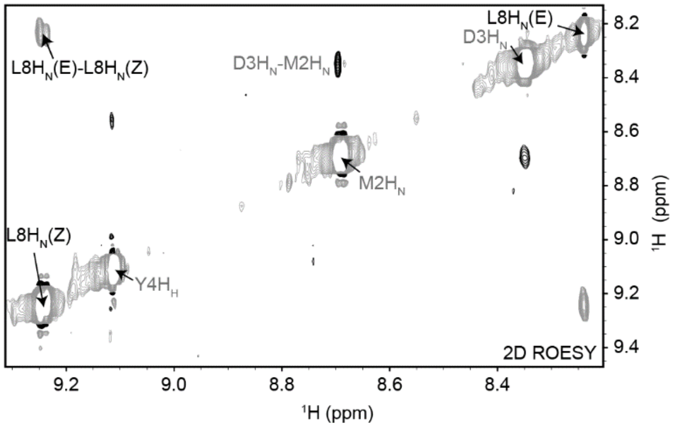

Figure S31: 2D ^{1}H-^{1}H ROESY spectrum of α-hydrazino peptide **h(8)**, which was recorded at 200 ms mixing time at 2 mM peptide concentration and 25 °C using a 600 MHz spectrometer. Antiphase cross-peaks (grey) indicate the presence of chemical exchange between different conformational forms in solution.

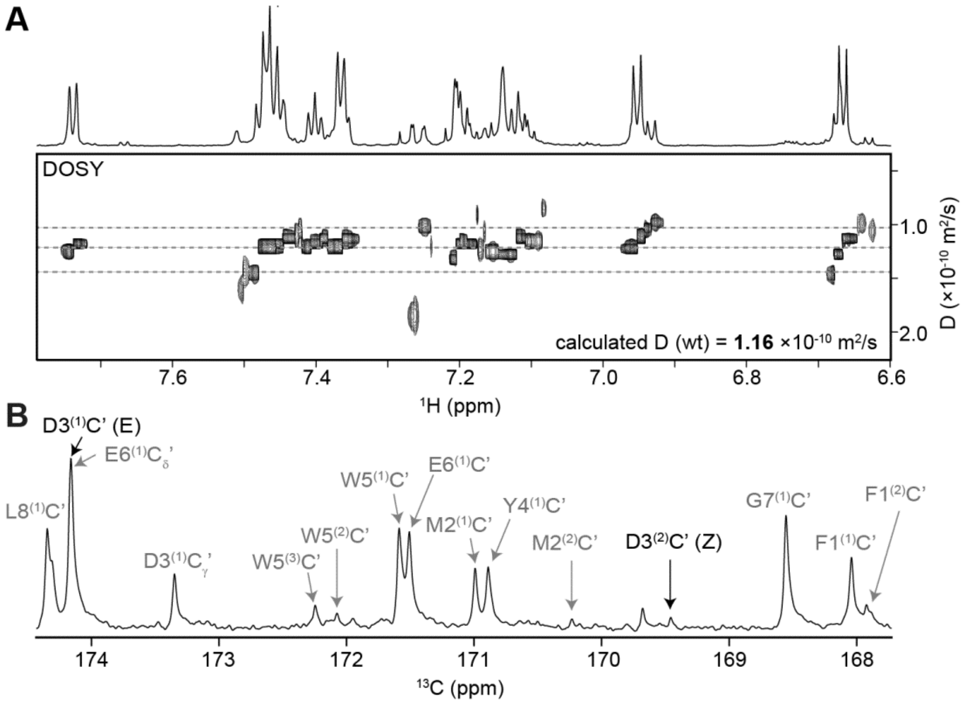

Figure S32: Conformational equilibrium of α-hydrazino peptide **h(4)** forms in DMSO-d$_6$. **A:** Aromatic region of 2D DOSY spectrum of α-hydrazino peptide **h(4)**. Diffusion coefficient standard error in the measurements was 0.05×10^{-10} m^2/s. The reference value D for the parent peptide was calculated with HYDROPRO[1]. **B:** Carbonyl (C') region of 1D ^{13}C NMR spectrum. C'$_\gamma$ and C'$_\delta$ indicate the chemical shift of the carbonyl group in the Asp and Glu side chain, respectively.

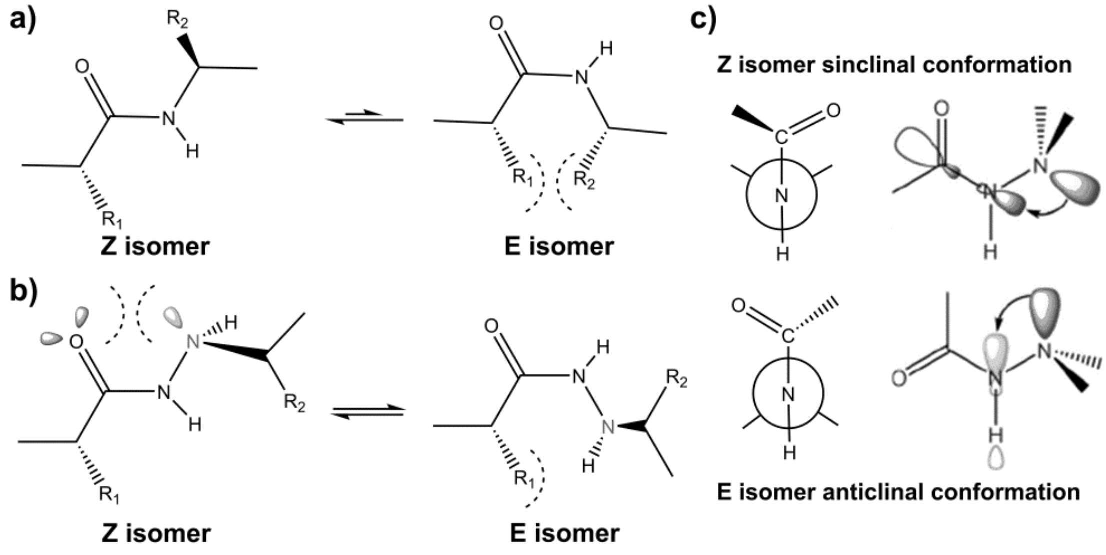

Figure S33: Factors affecting E-Z isomerism in α-hydrazino peptides. **a)** Steric clashes of side chains in E peptide bond isomers. **b)** Electronic repulsion between the carbonyl oxygen and the N$^\alpha$ lone pair in Z isomer with similar steric hindrance in E isomer. **c)** N$^\alpha$lp- σ_{NC}* or N$^\alpha$lp- σ_{NH}* hyperconjugation effects, reported by E. Gloaguen, *et al.*, *Angew. Chem. Int. Ed.* **2014**, *53 (50)*, 13756.

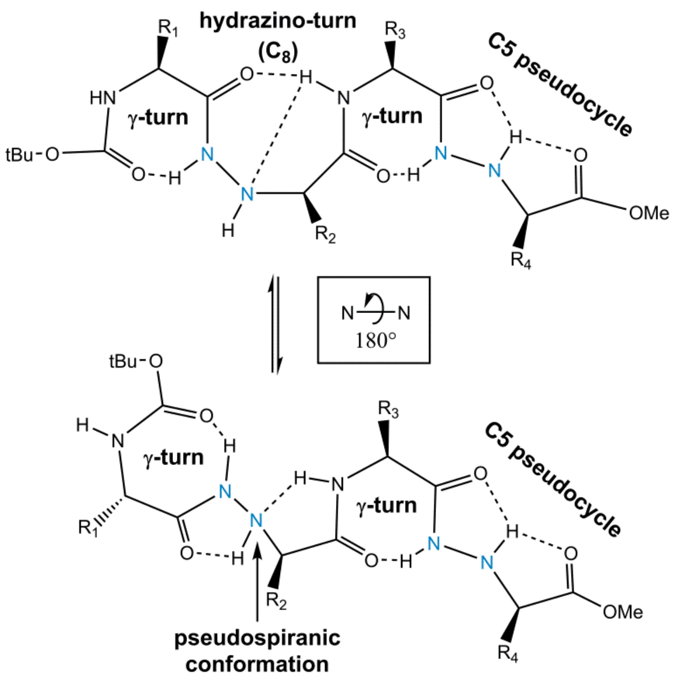

Figure S34: Conformational equilibrium for the major Z conformer of compound Boc-(Phe-α-hAla)$_2$-OMe in $CDCl_3$ related to rotation around N-N bond. Observed structural elements are assigned by the number of atoms, comprising the hydrogen bonded ring (C5, C8). Adopted from S. Acherar, *et al.*, *Eur. J. Org. Chem.* **2013**, (25), 5603.

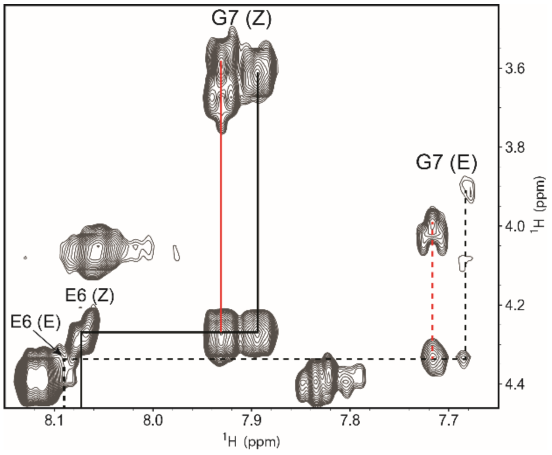

Figure S35: NOESY spectrum of α-hydrazino peptide **h(8)** recorded on 2 mM sample at 25 °C on an 800 MHz NMR spectrometer. Two sets of resonances for G7 H_N for each geometrical isomer (solid or dashed line) are presented in red or black.

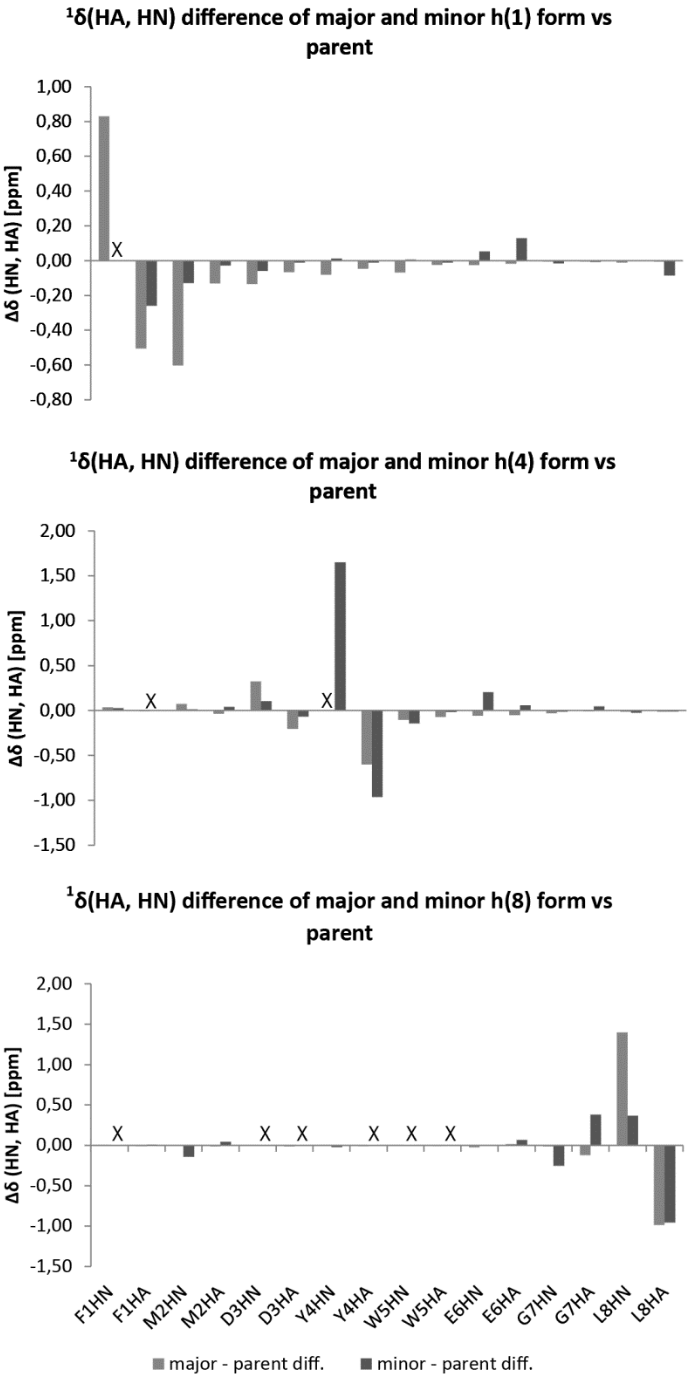

Figure S36: Chemical shift differences in H_N and Hα resonances of major and minor forms of α-hydrazino peptides compared to the parent peptide in DMSO-d_6 solution. X designates the absence of calculated value due to the absence of separate signal assignment for major and minor form in NMR spectra.

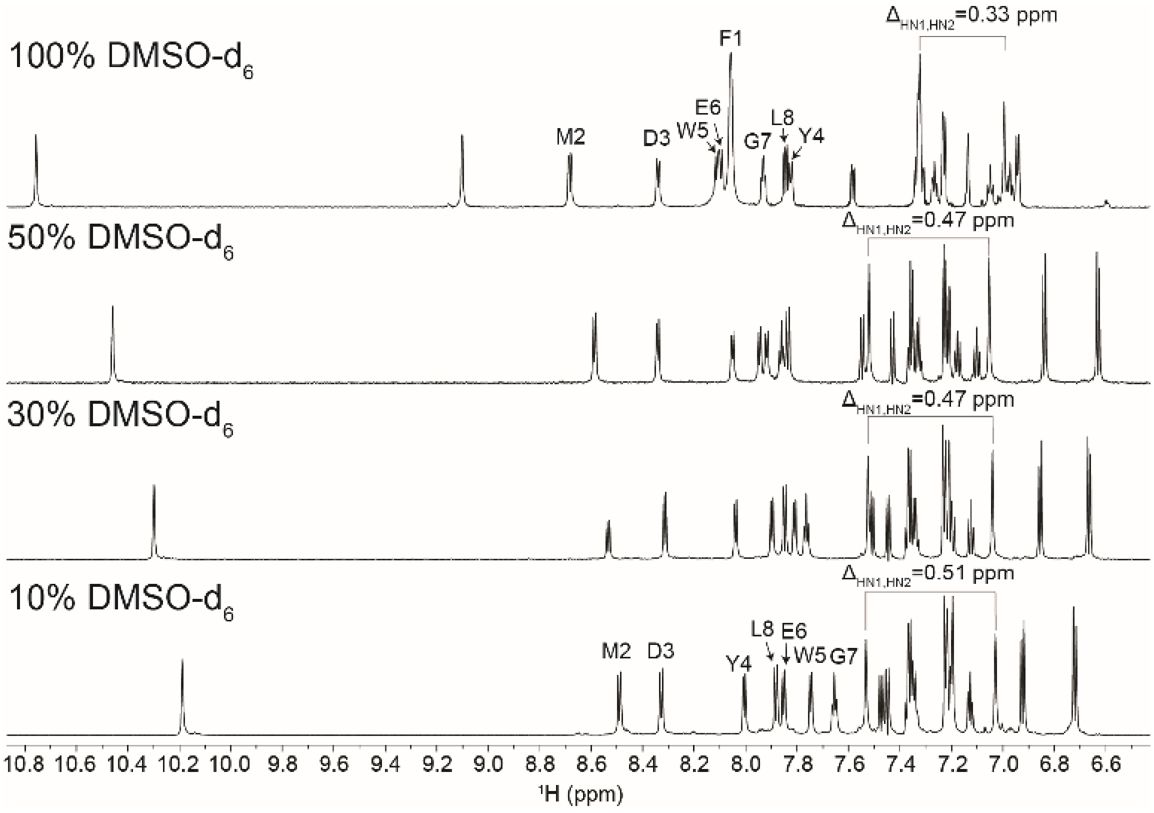

Figure S37: ^{1}H NMR spectra comparison of parent peptide in various DMSO-d_6/H_2O volume ratios with chemical shift differences ($\Delta\delta$) between C-terminal amide protecting group H_N protons. All spectra were recorded on 2 mM peptide samples on an 800 MHz spectrometer at 25 °C.

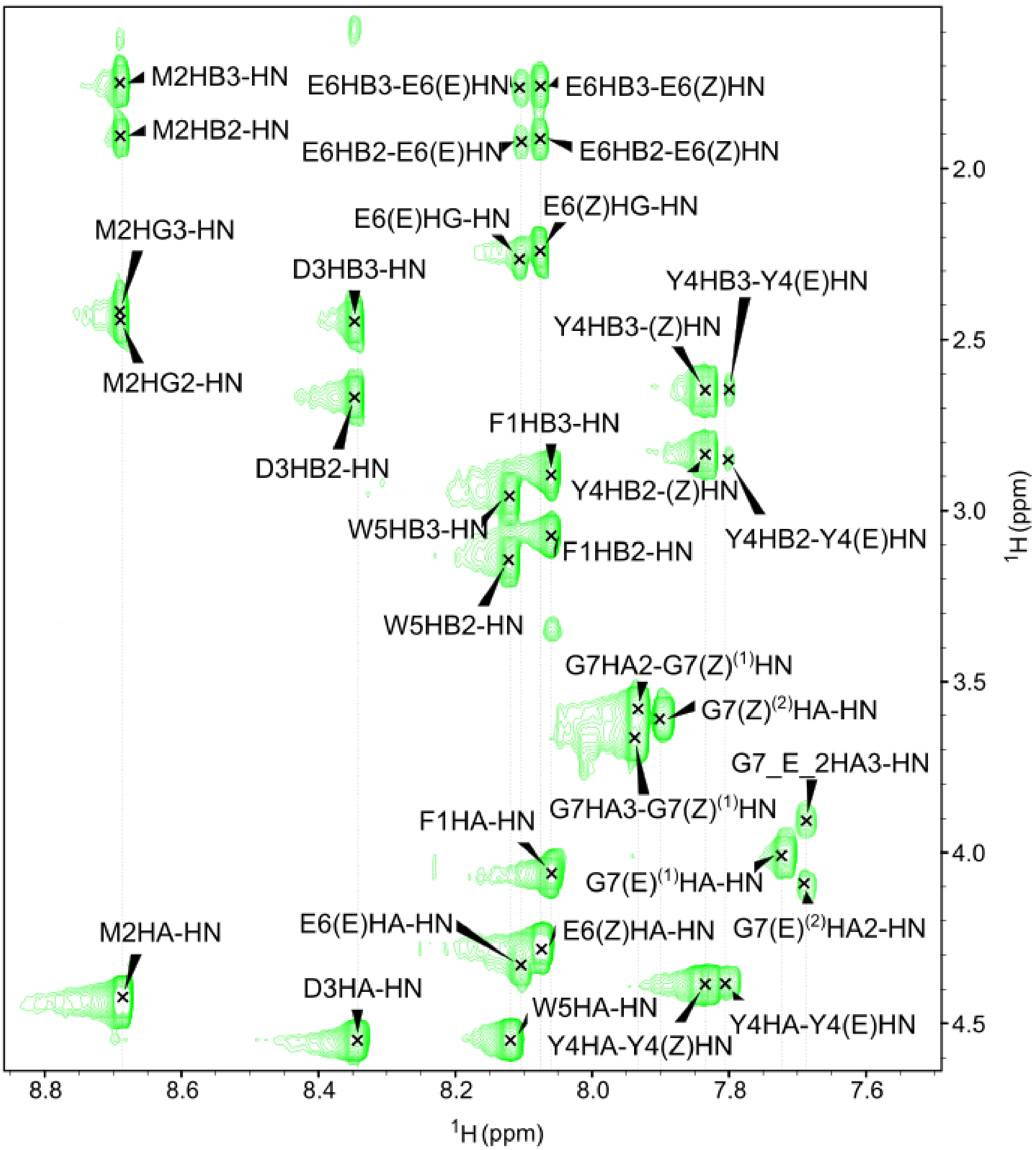

2D(1H,1H) TOCSY, 80 ms MLEV, 2 mM **h(8)**, 100% DMSO-d6, 25°C, 600MHz

Figure S38: 2D ^{1}H-^{1}H TOCSY spectrum of α-hydrazino peptide **h(8)**, revealing the identification of spin systems (HA, HB, HG) for amidic HN protons of different residues in the sequence. Unless stated otherwise, the assignment without additional numbers corresponds to the major form. Conditions of the measurement are provided below the spectrum.

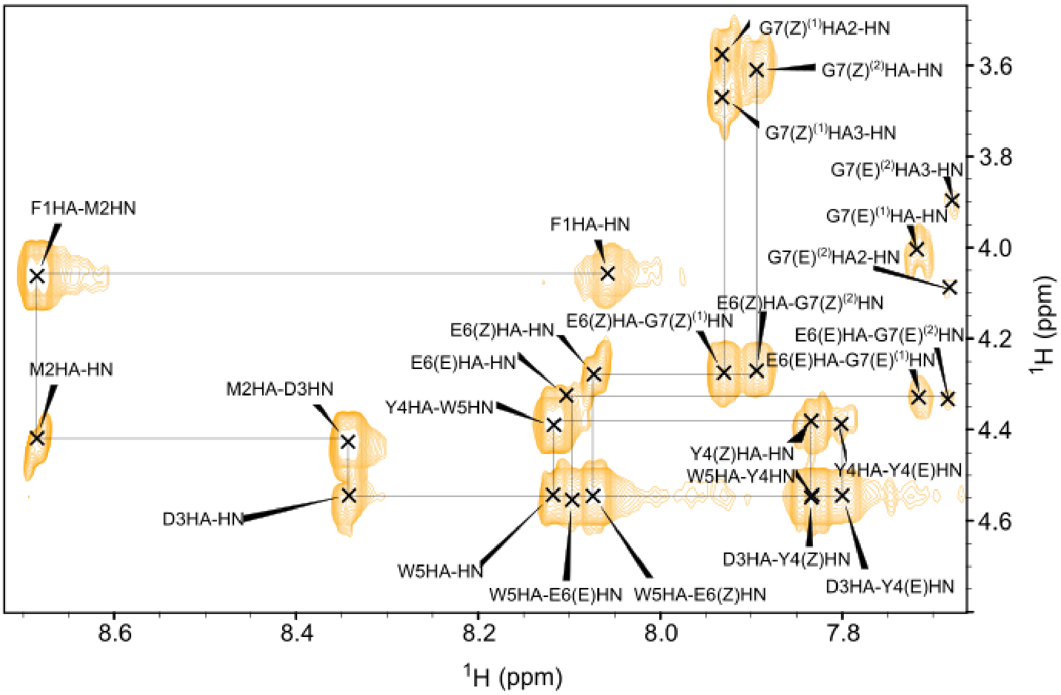

2D(1H,1H) NOESY,τm=150 ms, 2 mM **h(8)**, 100% DMSO-d6, 25°C, 600MHz

Figure S39: 2D ^{1}H-^{1}H NOESY spectrum of α-hydrazino peptide **h(8)**, revealing the sequential walk along the peptide main chain between amide HA protons and HN protons of the next residue. Unless stated otherwise, the assignment without additional numbers corresponds to the major form. Conditions of the measurement are provided below the spectrum.

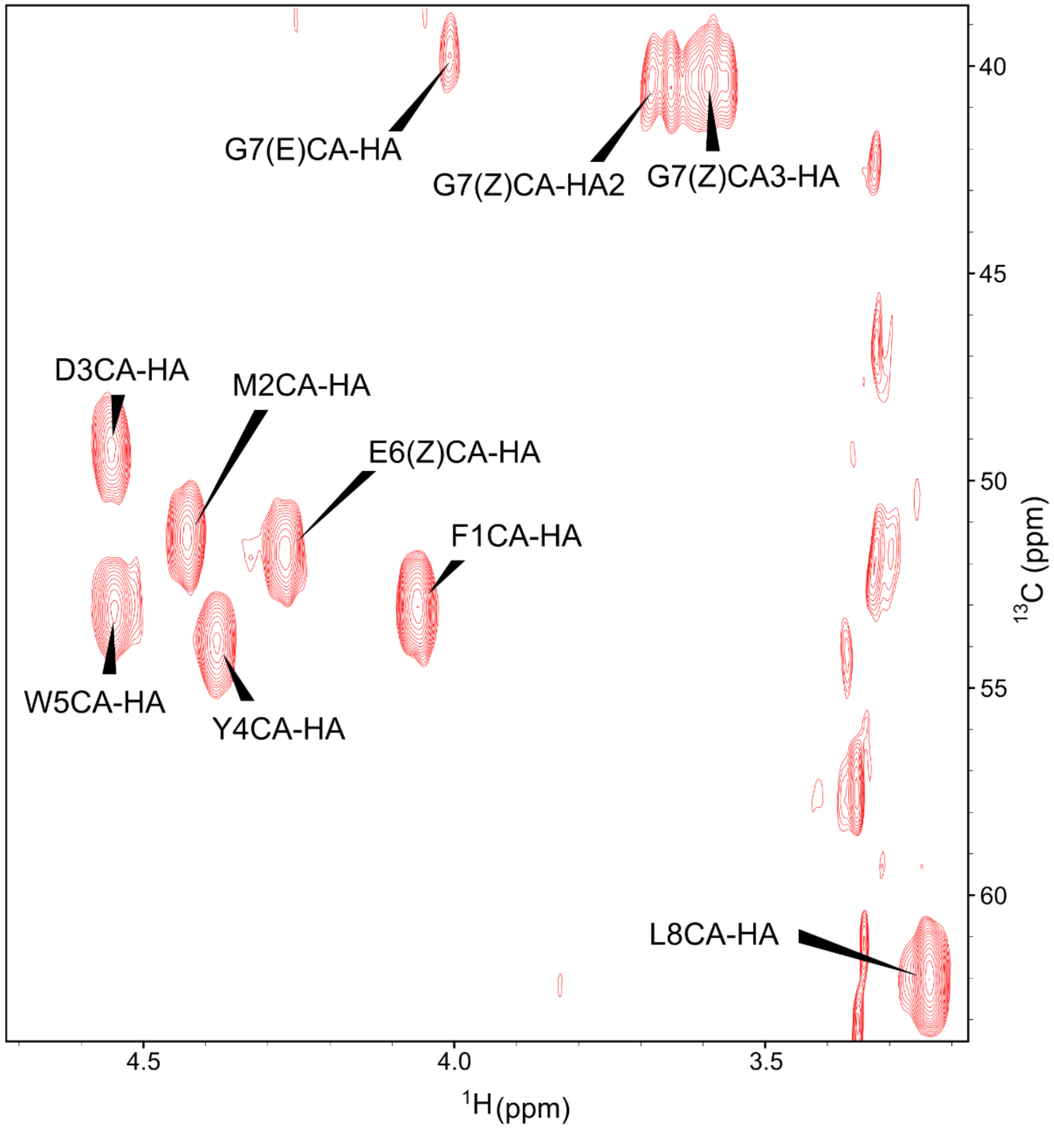

2D (13C,1H) HSQC, 2 mM **h(8)**, 100% DMSO-d6, 25°C, 600MHz

Figure S40: 2D ^{1}H-^{13}C HSQC spectrum of α-hydrazino peptide **h(8)**, providing information on the main chain Cα resonances based on CA-HA correlations in the 2D spectrum. Unless stated otherwise, the assignment without additional numbers corresponds to the major form. Conditions of the measurement are provided below the spectrum.

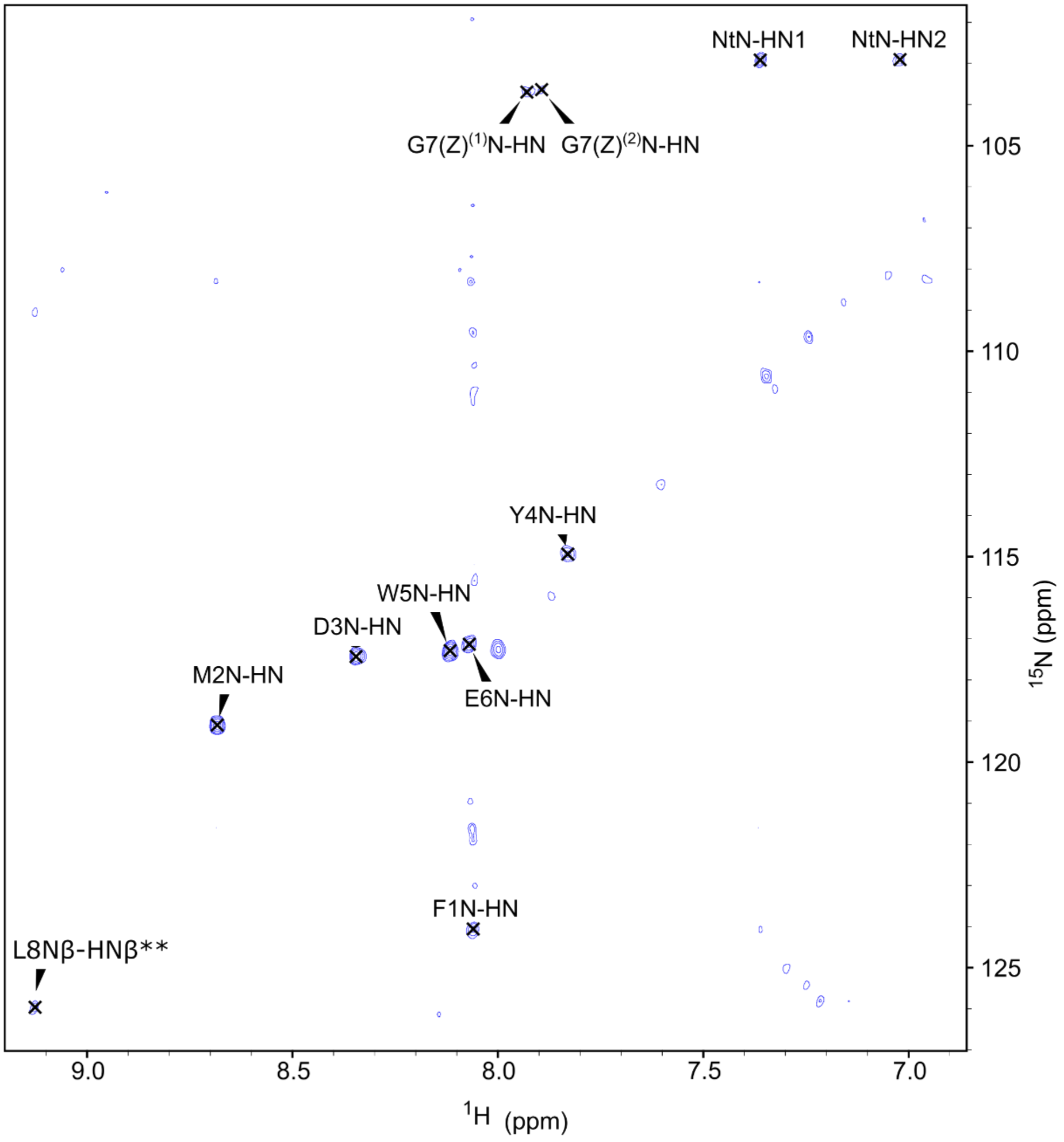

2D(15N,1H) HSQC, 2 mM **h(8)**, 100% DMSO-d6, 25°C, 600MHz

Figure S41: 2D ^{1}H-^{15}N HSQC spectrum of α-hydrazino peptide **h(8)**, providing information on the amide N resonances based on amide N-HN correlations in the main chain. Unless stated otherwise, the assignment without additional numbers corresponds to the major form. L8Nβ chemical shift was later corrected to 141.7 ppm, with the correlation observed at this value (126 ppm) due to too narrow region of NMR measurement. Conditions of the measurement are provided below the spectrum.

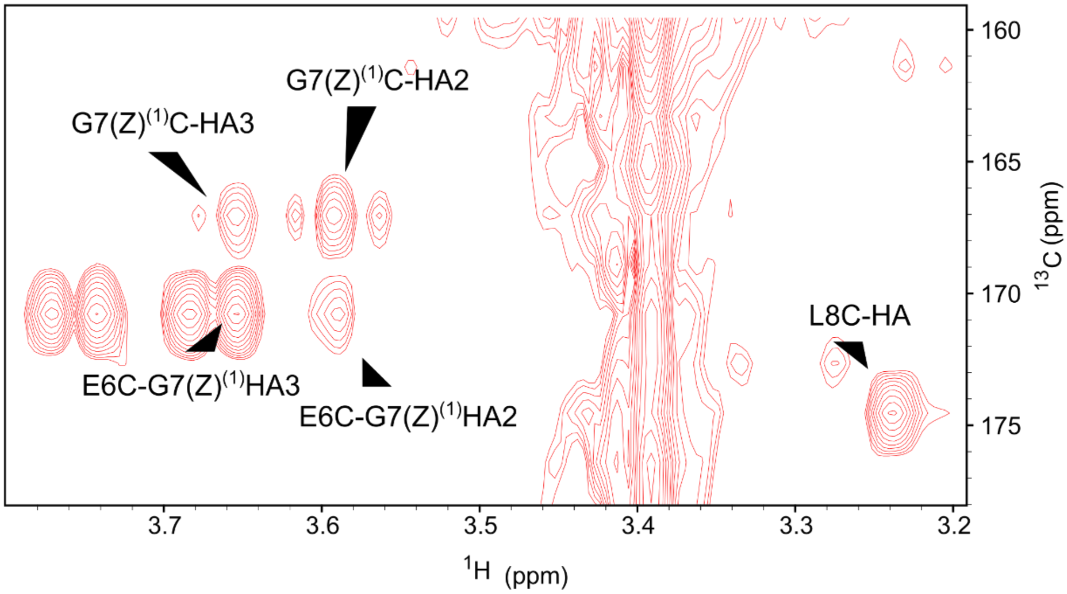

2D(13C,1H) HMBC, 2 mM **h(8)**, 100% DMSO-d6, 25°C, 600MHz

Figure S42: 2D ^{1}H-^{13}C HMBC spectrum of α-hydrazino peptide **h(8)**, providing information on the intra- and inter-residue connectivity via carbonyl C – HA correlations. Unless stated otherwise, the assignment without additional numbers corresponds to the major form. Conditions of the measurement are provided below the spectrum.

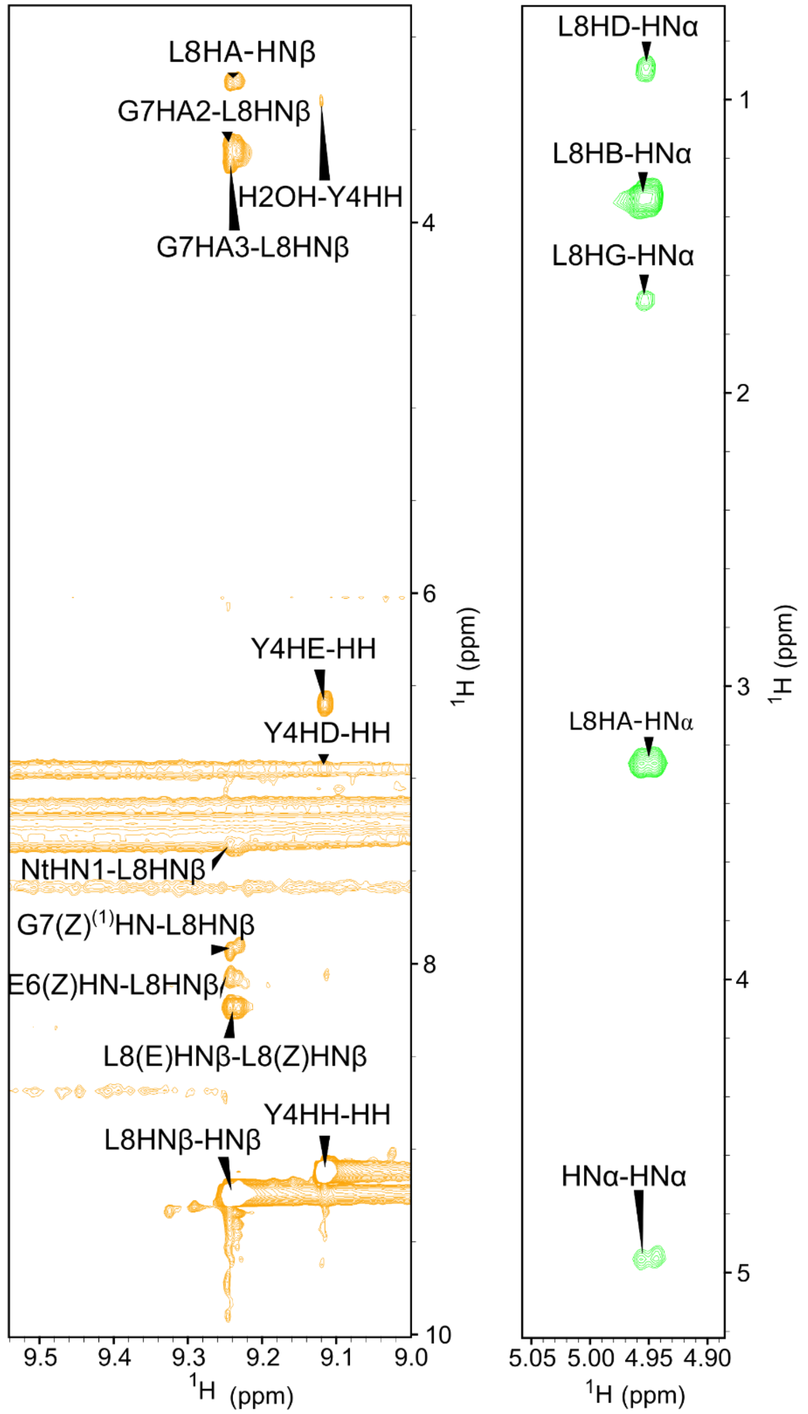

2D(1H,1H) NOESY,τm=150 ms, 2 mM **h(8)**, 100% DMSO-d6, 25°C, 600MHz

2D(1H,1H) TOCSY, 80 ms MLEV, 2 mM **h(8)**, 100% DMSO-d6, 25°C, 600MHz

Figure S43: Left: 2D ^{1}H-^{1}H NOESY spectrum of α-hydrazino peptide **h(8)**, providing NOE correlations between atypical HNβ proton and protons in close proximity (HN, HA). Right: 2D ^{1}H-^{1}H TOCSY spectrum of α-hydrazino peptide **h(8)**, providing correlations between atypical HNα proton and protons in in the Leu8 spin system. Unless stated otherwise, the assignment without additional numbers corresponds to the major form. Conditions of the measurements are provided below the spectra.

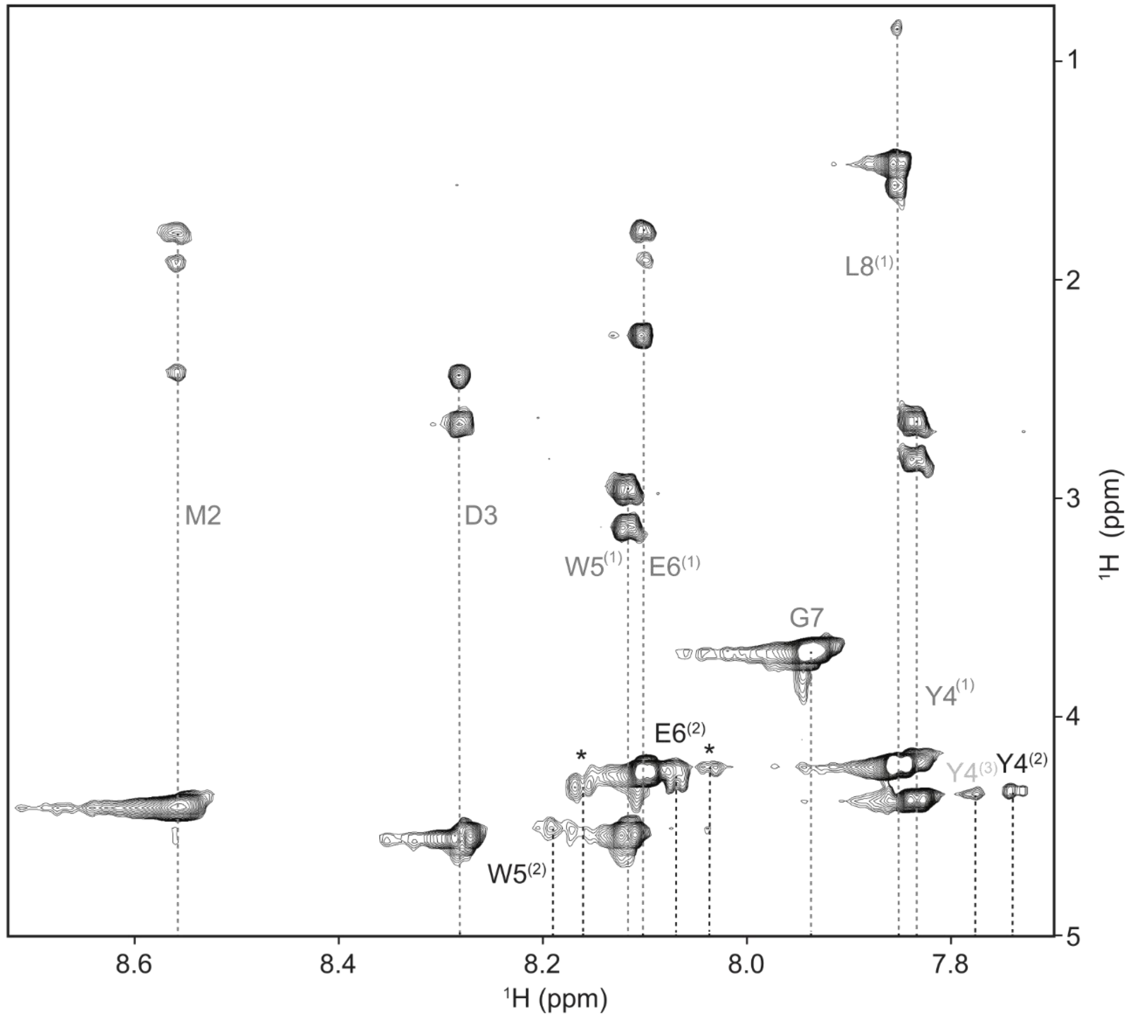

2D(1H,1H) TOCSY, 80 ms MLEV, 2 mM **h(1)**, 100% DMSO-d6, 25°C, 600MHz

Figure S44: 2D ^{1}H-^{1}H TOCSY spectrum of α-hydrazino peptide **h(1)**, revealing multiple sets of signals corresponding to multiple spin systems (Y4, W5 and E6) for amidic HN protons. Unless stated otherwise, the assignment without additional numbers corresponds to the major form. Conditions of the measurement are provided below the spectrum.

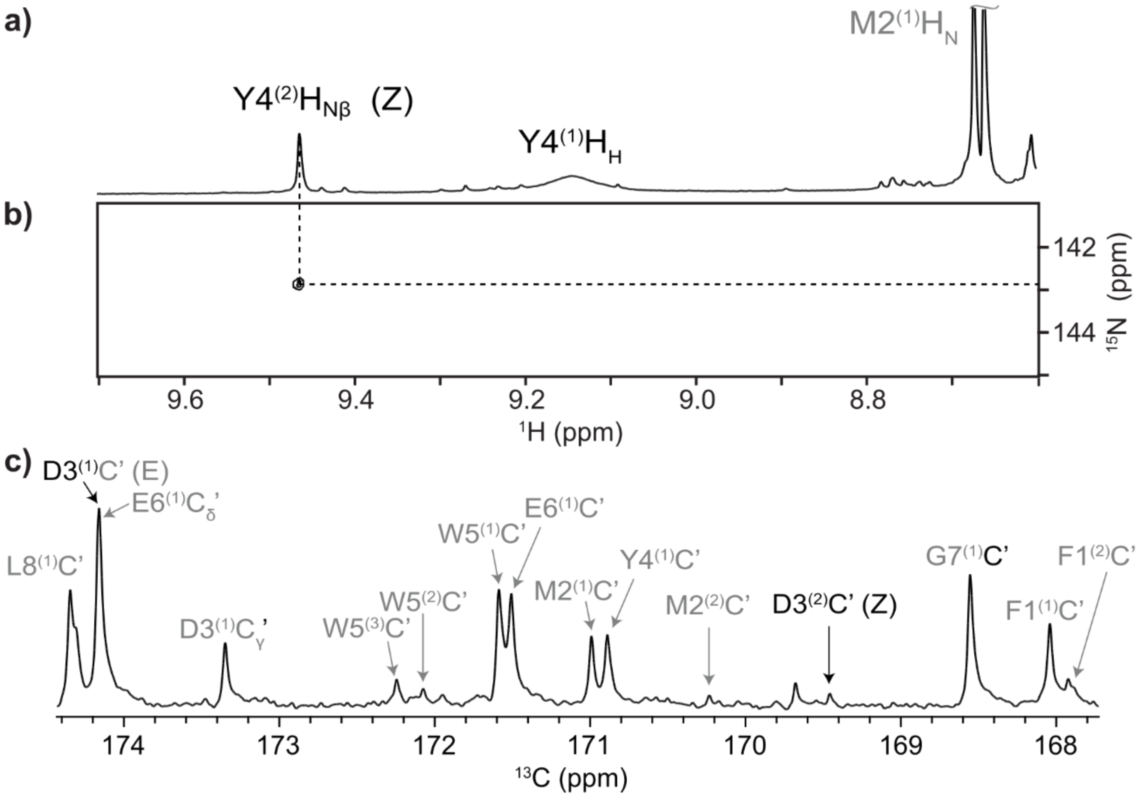

Figure S45: a) Hydrazidic region of 1D ^{1}H NMR of α-hydrazino peptide **h(4)**. b) ^{1}H-^{15}N HSQC spectrum region of amide N and proton, revealing a correlation of Y4(2)HN-N (minor form). Spectra were acquired at 2 mM peptide concentration. c) Carbonyl (C’) region of ^{13}C 1D spectrum of α-hydrazino peptide **h(4)**. Spectrum was acquired at 8 mM concentration of the peptide. Signal, corresponding to the carbonyl atom, involved in the E/Z isomerism, is highlighted in black. All spectra were acquired at 25 °C on a 600 MHz NMR spectrometer.

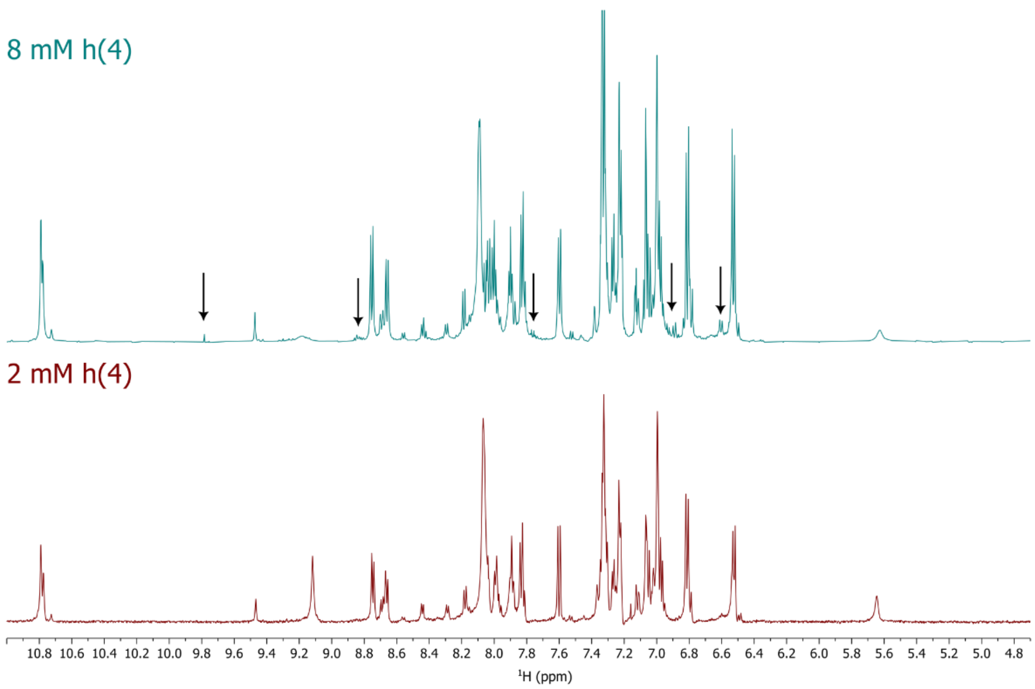

Figure S46: Comparison of ^{1}H NMR spectra of α-hydrazino peptide **h(4)** (amide and aromatic region) at 8 mM (above) and 2 mM (below) concentration. Arrows indicate additional signals, observed at higher concentrations, complicating the NMR assignment. Both spectra were acquired at 25 °C on 600 MHz NMR spectrometer.

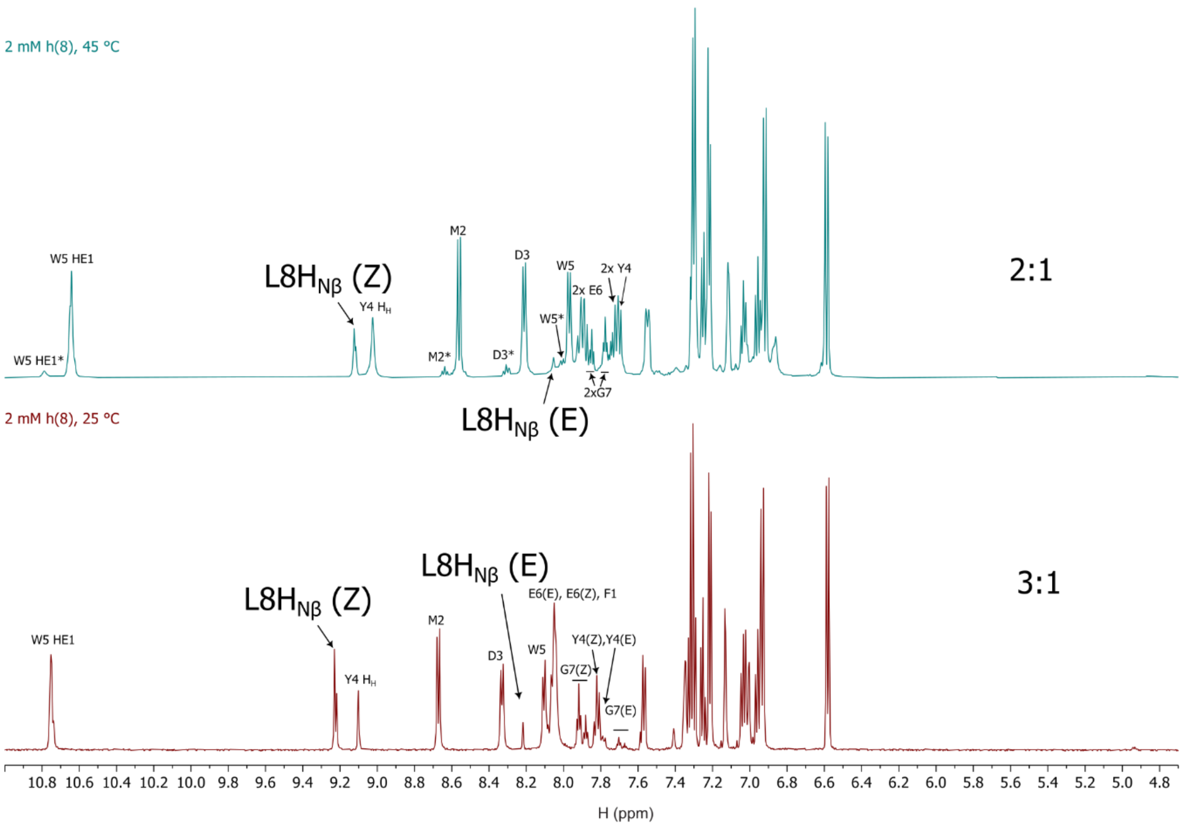

Figure S47: Comparison of ^{1}H NMR spectra of α-hydrazino peptide **h(8)** (amide and aromatic region) at 2 mM concentration, measured at 25 °C (below) and 45 °C (above). Assignment of the peaks was done according to the TOCSY spin systems, with additional signals corresponding to hydrazidic protons ($H_{N\beta}$) designated for L8, with Z/E annotation at 45 °C done based on the annotation at 25 °C, with ratios of both L8 HNβ signals provided on the right. Both spectra were acquired on a 600 MHz NMR spectrometer.
